# A Genome-Scale Vector Resource Enables High-Throughput Reverse Genetic Screening in a Malaria Parasite

**DOI:** 10.1016/j.chom.2015.01.014

**Published:** 2015-03-11

**Authors:** Ana Rita Gomes, Ellen Bushell, Frank Schwach, Gareth Girling, Burcu Anar, Michael A. Quail, Colin Herd, Claudia Pfander, Katarzyna Modrzynska, Julian C. Rayner, Oliver Billker

**Affiliations:** 1 Wellcome Trust Sanger Institute, Hinxton Cambridge CB10 1SA, UK

## Abstract

The genome-wide identification of gene functions in malaria parasites is hampered by a lack of reverse genetic screening methods. We present a large-scale resource of barcoded vectors with long homology arms for effective modification of the *Plasmodium berghei* genome. Cotransfecting dozens of vectors into the haploid blood stages creates complex pools of barcoded mutants, whose competitive fitness can be measured during infection of a single mouse using barcode sequencing (barseq). To validate the utility of this resource, we rescreen the *P. berghei* kinome, using published kinome screens for comparison. We find that several protein kinases function redundantly in asexual blood stages and confirm the targetability of kinases *cdpk1*, *gsk3*, *tkl3*, and PBANKA_082960 by genotyping cloned mutants. Thus, parallel phenotyping of barcoded mutants unlocks the power of reverse genetic screening for a malaria parasite and will enable the systematic identification of genes essential for in vivo parasite growth and transmission.

## Highlights

• Barcoded genetic modification vectors used to create a resource for *P. berghei*
• Parallel phenotyping of dozens of mutants can be performed using barcode sequencing
• A validation barseq screen of the kinome reveals false-positive and -negative rates
• Targetable kinase genes identified and verified with cloned mutants

## Graphical Abstract

**Figure:**
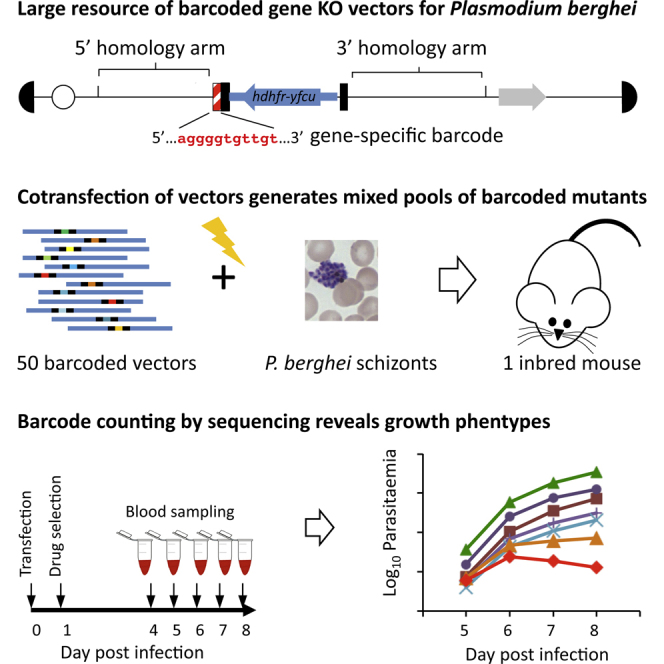

## Teaser

Gomes et al. present a large-scale resource of barcoded genetic modification vectors with high integration efficiency for *Plasmodium berghei*. The resource enables reverse genetic screening by allowing pools of barcoded mutants to be created in a single mouse and phenotyped in parallel by counting barcodes through next generation sequencing.

## Introduction

The rate at which the genomes of intracellular malaria parasites can be modified has remained largely unchanged since methods for gene targeting by homologous recombination were developed in *Plasmodium* (Crabb and Cowman, 1996; van Dijk et al., 1996; Wu et al., 1995). Some notable advances have recently improved transfection efficiency in *P. falciparum* through the application of zinc finger nucleases (Straimer et al., 2012) and CRISPR-Cas9 (Ghorbal et al., 2014; Wagner et al., 2014). However, no currently available method is efficient enough to enable reverse genetic screens, and transposon mutagenesis in *P. falciparum* is at present well short of genome saturation (Balu and Adams, 2006). As a result, more than half of the protein coding genes in *Plasmodium* genomes still lack functional annotation.

Genome-wide collections of mutants or genetic modification vectors have greatly facilitated the discovery of gene functions in model organisms (Ni et al., 2011; Sarov et al., 2006; Skarnes et al., 2011; Winzeler et al., 1999). In malaria parasites, in contrast, efforts to scale up reverse genetics have suffered from a combination of low rates of homologous recombination and a high content of adenine and thymine (A+T) nucleotides that renders *Plasmodium* DNA difficult to engineer in *E. coli*. A malaria parasite of rodents, *P. berghei*, offers the most robust system for genetic manipulation with relatively high transfection efficiency (Janse et al., 2006a). In this species homologous integration can be boosted further by transfecting linear vectors with long (4–8 kb) homology arms (Pfander et al., 2011). Despite its high A+T content (>77%), *P. berghei* genomic DNA (gDNA) can be propagated efficiently in *E. coli* as large genomic inserts of up to 20 kb using a low-copy bacteriophage N15-derived linear plasmid with covalently closed hairpin telomeres (Godiska et al., 2010). In contrast to high-copy circular plasmids, an N15-based arrayed gDNA library achieved nearly complete genome coverage with sufficient insert size to represent the majority of *P. berghei* genes in their entirety. Clones from this library can be converted into gene targeting and tagging vectors in 96 parallel liquid cultures using robust protocols (Pfander et al., 2011), which exploit highly efficient homologous recombination mediated by the Red/ET recombinase system of *lambda* phage in *E. coli* (Zhang et al., 2000).

To accelerate the functional analysis of all *P. berghei* genes we here present a genome-scale community resource of long-homology genetic modification vectors that are individually quality controlled by sequencing and carry gene-specific molecular barcodes. The availability of more than 2,000 genome modification vectors raises the possibility of generating a large library of cloned and genotyped *P. berghei* mutants of the type that has enabled global genetic screens in yeast (Giaever et al., 2002; Winzeler et al., 1999). However, in *P. berghei* the lack of continuous in vitro culture of blood stages would limit the utility of such a clone collection. Signature-tagged mutagenesis, whereby thousands of mutants are simultaneously screened in a pooled approach (Hensel et al., 1995; Langridge et al., 2009; Mazurkiewicz et al., 2006), therefore offers a more attractive strategy for scaling up reverse genetics in *P. berghei*.

We have used the modification vector resource to enable such systematic screens for a *Plasmodium* parasite. We demonstrate that cotransfecting multiple gene knockout vectors in the same electroporation reproducibly generates complex pools of barcoded *P. berghei* mutants, and develop a barcode sequencing (barseq) approach (Smith et al., 2009) to phenotype the growth rates of all mutants within the pool over the course of an infection. To validate the approach, we compared a barseq knockout screen of protein kinases with the conventional kinome screen by Tewari et al. (2010). This comparison showed high reproducibility with previous data, but the sensitivity and robustness of the barseq approach also identified additional targetable genes. Our analysis demonstrates the power of barseq screening to robustly provide growth-rate phenotypes for dozens of mutants in single mice, and opens up the possibility for large-scale reverse genetic screens for multiple areas of *Plasmodium* biology.

## Results

### A Resource of Efficient Gene Targeting Vectors for *P. berghei*

**Figure 1:**
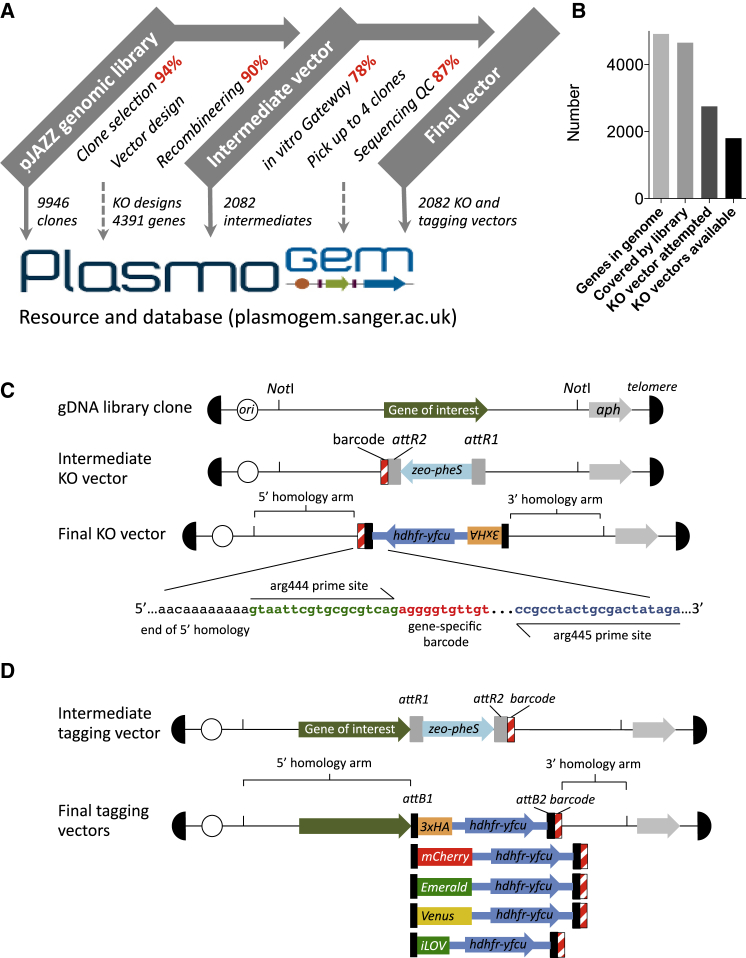
*Plasmo*GEM: A Genome Scale Free Resource of Genetic Modification Vectors for *P. berghei* Reverse Genetics (A) A diagram of the modular vector production process showing the efficiency at each step (red), as well as resources (gray boxes) and data (dashed lines) submitted to the database. (B) Genome coverage achieved to date. (C) Schematic showing knockout vector designs and locations of the gene-specific molecular barcode included in each vector. (D) Default C-terminal epitope-tagging vector and a panel of alternative fusion tags.

To generate a genome-scale resource of gene knockout vectors, we used a modular pipeline for recombinase mediated engineering in *E. coli* (Pfander et al., 2011). The parasite gene of interest was first replaced in appropriately chosen gDNA clones with a marker for positive and negative selection in *E. coli* using Red/ET recombinase-mediated engineering. The bacterial markers were then exchanged under negative selection for a drug resistance cassette for *P. berghei* in a single in vitro Gateway recombinase reaction. When applied to the 2,781 *P. berghei* genes that have any level of functional annotation (57% of the genome), a first pass of the production pipeline yielded gene deletion vectors for 1,868 different protein coding genes of the core nuclear genome (Figures 1A–1C). These vectors form the foundation of the Plasmo*dium ge*netic *m*odification resource, *Plasmo*GEM (Figure 1A), which can be viewed and requested through a searchable database at http://plasmogem.sanger.ac.uk (Schwach et al., 2015).

The vector production strategy can also be adapted to generate other modifications, including C-terminal tagging vectors, of which there are currently 278 in the resource. Tagging vectors are constructed with a C-terminal triple HA epitope tag by default. For each final vector we also make uncloned intermediate constructs available, which users can convert into targeting vectors with different functionality. A panel of Gateway entry clones are available which contain fusion tags for epitopes and different fluorescent proteins (Figure 1D). Each *Plasmo*GEM vector carries a gene-specific molecular barcode of 10–11 nucleotides that uniquely identifies genetically modified parasites derived from it (Figures 1C and 1D), and all final *Plasmo*GEM vectors contain the *hdhfr-yfcu* marker that enables positive and negative selection in vivo (Braks et al., 2006).

Manual construct design became rate limiting for vector production, and we therefore created a suite of computational tools to select gDNA library clones; to choose 50 bp homology regions for recombination in *E. coli*; to design oligonucleotides, generate sequence maps, assign barcodes, and track vectors through the production process; and to automate quality control (Schwach et al., 2015). The long homology arms of *Plasmo*GEM vectors (average sum of both arms 7.4 ± 2.9 kb SD) enhance recombination frequency in *P. berghei* compared to conventional designs (Pfander et al., 2011), but they also pose a risk that unintended mutations get inadvertently incorporated into the parasite genome. Homology regions and barcode modules of all targeting vectors are therefore confirmed by next-generation sequencing and pass stringent QC standards before they are used (for details, see https://plasmogem.sanger.ac.uk).

### Parallel Phenotyping of Dozens of *Plasmo*GEM Mutants by Barcode Counting

To examine whether the improved integration efficiency of *Plasmo*GEM vectors would permit the reproducible generation and phenotyping of mixed pools of barcoded mutants, we selected from the resource 46 vectors targeting protein kinases that had previously been part of a systematic deletion analysis using conventional vectors (Tewari et al., 2010). Four sexual and mosquito-expressed genes known to be dispensable for asexual growth were also included to serve as reference genes to which growth rates of all other mutants could be compared. This reference set was comprised of knockout vectors for the major surface proteins of the ookinetes, P25 and P28 (Tomas et al., 2001); a secreted ookinete adhesive protein, SOAP (Dessens et al., 2003); and a C-terminal tagging vector for the redundant *p230p* gene (van Dijk et al., 2010). In addition, three further control vectors were included to assess our ability to detect reduced growth rates in blood stages. One of these targeted plasmepsin IV, an aspartic protease involved in hemoglobin degradation whose deletion in *P. berghei* results in attenuated growth (Spaccapelo et al., 2010). We predicted parasite growth would be reduced by a deletion vector for PBANKA_110420, which encodes the E1β subunit of the mitochondrial branched chain α-ketoacid dehydrogenase (BCKDH), given that deleting the E1α subunit of the same complex has a clear growth phenotype (Oppenheim et al., 2014). A third attenuating knockout vector targeted PBANKA_140160, a putative methyl transferase of unknown function, which emerged as a slow-growing mutant from a preliminary screen of metabolic enzymes (data not shown).

**Figure 2:**
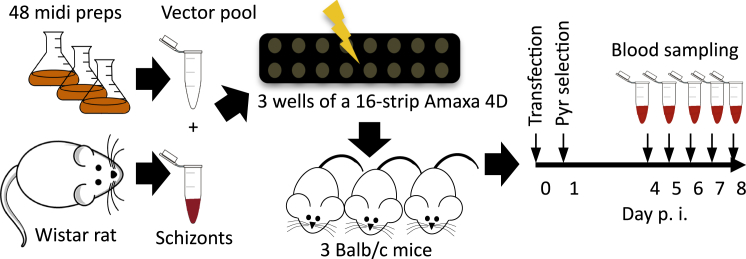
Schematic Representation of a Typical Barcode Sequencing Experiment For each experiment three inbred mice are infected from a separate transfection of the same vector pool.

**Figure 3:**
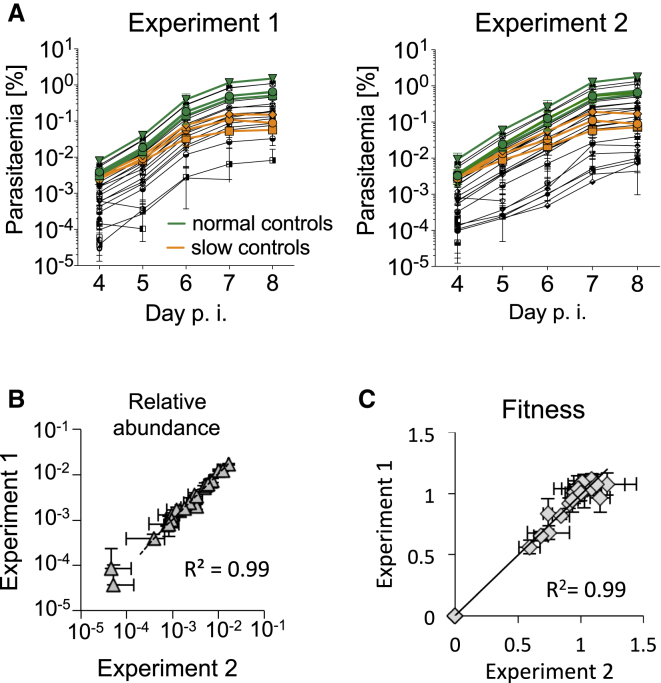
Reproducibility of Independent Barcode Counting Experiment with Respect to the Abundance and Relative Replication Rates of All Barcodes (A) Each experiment involved three replicate transfections of a different schizont culture performed on a different day and using independently prepared vector pools. Error bars show standard errors (n = 3 per experiment). Green lines, four sexual stage genes (*p25*, *p28*, *p230p* 3xHA tag, and *soap*). Orange lines, three attenuated mutants (plasmepsin IV, PBANKA_110420, PBANKA_140160). Twenty-two mutants are shown in total. See Figure S1 for genotyping data. (B) Linear regression analysis of mean abundance values for the two experiments shown in (B). All barcodes present until day 8 posttransfection were included. Error bars show standard errors of the mean (n = 3). (C) Regression analysis of average mean fitness for each barcode between days 5–8 posttransfection for the two biological replicates in (B). Fitness is calculated from the replication rate of the gene-specific barcode relative to the mean of the four sexual stage reference genes. Error bars show standard errors (n = 3). See Table S1 for fitness measurements for individual vectors, and Table S4, illustrating data analysis.

Schizonts cotransfected with a cocktail of 48 vectors and injected into mice (Figure 2) gave rise to drug resistant parasites 4 days later. This was indicative of an overall transfection efficiency of ∼10^−4^ and suggested that roughly 2,500 independent recombination events occurred in a transfection, enough to generate a complex mixture of mutants. Blood samples were subsequently collected exactly every 24 hr from day 4, gDNA was extracted, and vector-specific barcodes were amplified by a polymerase chain reaction (PCR) with a generic primer pair (see Figure S1A available online) and counted on a benchtop next-generation sequencer (Figure 2). In each of two replicate experiments, the same 22 barcodes from the vector pool were robustly detected and yielded nearly identical growth curves (Figure 3A). Southern hybridization of separated chromosomes showed evidence for vector integration events throughout the genome (Figure S1B). Long-range PCR products confirmed integration events for 17 of the 22 replicating barcodes (Figure S1C).

To assess the accuracy of barcode counting, we analyzed the same infected blood samples using two different methods for turning the initial PCR product after barcode amplification into sequencing libraries. The staged PCR strategy shown in Figure S1A and a conventional adaptor ligation protocol performed equally well, producing highly correlated barcode counts (Figure S1D). The PCR strategy proved faster and more economical and was therefore used in subsequent experiments. Figure S2A illustrates for a typical experiment the relative abundances of barcodes in a pool of transfected vectors and in infected mice 7 days after transfection, as determined by the PCR method.

**Figure 4:**
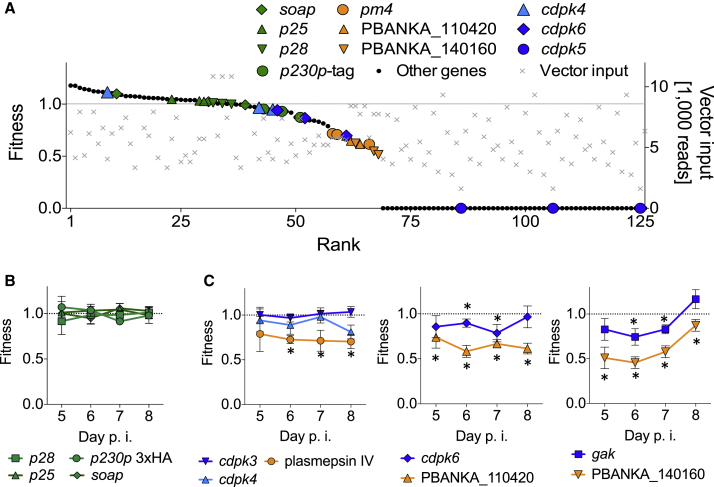
Fitness Measurements Obtained with *Plasmo*GEM Vectors Targeting Protein Kinases (A) Distribution plot generated from a ranked list of day 7 fitness values measured in triplicate for each of 42 genes in experiment 1 (left axis). The relative abundance of a targeting vector in the electroporation cuvette at the moment of transfection (gray crosses, right axis) did not predict whether a mutant could be obtained. See Figure S2 for relative abundance data of a representative replicate experiment. (B) Fitness of reference mutants averages 1 by definition. Error bars show standard errors (n = 6). (C) Fitness of selected mutants. Error bars as in (B). Asterisk, different from reference mutants as determined by a two-sided t test corrected for multiple testing (p < 0.01; n = 6).

To analyze growth curves derived from barcode counting we considered two parameters: (1) the relative abundance of each barcode within the pool, and (2) the relative fitness of each mutant, i.e., the rate at which its abundance changed each day. As expected, the four barcodes corresponding to control genes redundant for asexual development replicated rapidly. These were taken to represent wild-type growth (fitness w = 1). Relative abundance and growth rates were both highly reproducible for each barcode between technical and biological replicates (Figures 3B and 3C). We propose that the shape of a growth curve provides a quantitative measure for the fitness of a mutant. In contrast, the relative abundance of a mutant within a pool we consider less informative, since it may be influenced by a number of additional factors, such as the abundance of a vector in the transfection cocktail, the length of its homology arms, or any local variation in recombination rates. Plotting day 7 fitness from a ranked list (Figure 4A) showed that while the relative abundance of vectors in the transfection cocktail varied by up to one order of magnitude (Figure 4A, right axis), this was not a predictor of fitness of the resulting mutants. Fitness is therefore driven by growth rate, not by the amount of a given vector in the starting pool. The attenuating control vectors were associated with a measurable reduction in parasite fitness to between 0.60 and 0.73, as expected. The majority of protein kinase mutants either had wild-type fitness or were not detected (w = 0). While all reference barcodes robustly replicated close to the average fitness of 1.0 (Figure 4B), the attenuating vectors and some kinase mutants showed statistically significant reductions in fitness that were consistently measured across different days of the infection (Figure 4C). Taken together, these data strongly suggest that barcode counting can be used to phenotype large numbers of mutants in parallel.

We hypothesized that in pools some slow-growing mutants may be outcompeted by faster-growing mutants before their barcodes can be detected. To eliminate this potential source of error, we performed a second-pass screening strategy by pooling only the slow or nonreplicating vectors from the previous experiments and transfecting them together with the reference set. This allowed us to measure the fitness of four additional mutants, one whose vector had a very low integration efficiency (PBANKA_040940), and three that were characterized by low growth rates during part or all of the infection (Figure S2B). These data suggest that the size and complexity of a vector pool can be increased only at the expense of losing more slow growing mutants. However, this loss can be compensated in a second-pass screen of vectors that are not detected in the first experiment.

### Barcode Counting Reveals Protein Kinase Mutants

**Figure 5:**
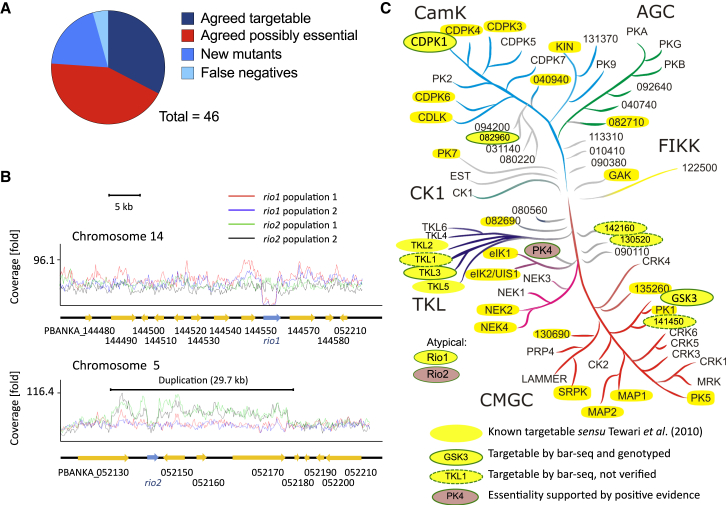
Barcode Sequencing Is Validated by a Comparison with Published Data and Genotyping of Mutants (A) Barseq screen of 46 *Plasmo*GEM vectors targeting protein kinases compared to the conventional kinome screen by Tewari et al. (2010). (B) Read coverage from whole-genome sequencing of highly enriched mutant populations showing deletion of *rio1* in a haploid genome (upper panel), and insertion of a *rio2* knockout vector associated with stabilization of a 29.7 kb duplication including *rio2*. See Figure S5 for additional genotyping data. (C) Updated tree showing targetable and essential *P. berghei* protein kinases. Targetability of *cdpk1* was independently shown by Jebiwott et al. (2013). A role for PK4 in blood stage growth was demonstrated by Zhang et al. (2012). See Figures S3 and S4 for genotype confirmation of cloned mutants for the knockouts.

To assess the accuracy of barseq screening, we compared the combined results from five barseq experiments with the data from our previous knockout screen, which used conventional vectors in a gene-by-gene approach with careful genotyping of cloned mutants (Tewari et al., 2010). For the majority of genes (76.1%) covered by both studies, the replication of barcodes in the mixed pool of mutants was predicted by previous data for their targetability (Table S1; Figure 5A). Two genes shown previously to be dispensable in blood stages (Tewari et al., 2010) could not be targeted in barseq screens. Failure of these vectors to integrate was not due to low amounts of vector DNA in the transfection cocktail and in the case of *pk7* was reproduced when vectors were transfected individually. Absence of integration may be the result of low recombinogenicity at the target locus, or individual vector designs may interfere with the expression of essential neighboring genes. Although the fraction of technical failures was small, these observations reaffirm the need to confirm genetic essentiality by conditional methods, just as in conventional gene-by-gene studies.

Although some false negatives can be tolerated, genetic screens rely critically on the rate of false positives being very low. In barcode sequencing experiments false positives may result from episomal replication of vector DNA or from nonhomologous integration of targeting vectors. Although the former is commonly observed in *P. berghei* after transfecting vectors that originate from circular plasmids, we have not yet encountered spontaneous circularization of *Plasmo*GEM vectors, which rely on the expression of a phage telomerase to replicate in a linear form in *E. coli* (data not shown). The strongest candidates for false positives in our pilot screen were ten protein kinase genes for which barcode counting revealed evidence for their disruption by *Plasmo*GEM vectors, while previous studies had failed to delete the same genes using conventional vectors (Sebastian et al., 2012; Tewari et al., 2010). We selected six of these genes for validation by targeting them individually.

Two genes, *cdpk1* and *gsk3*, have been considered potential drug targets in *P. falciparum* (Droucheau et al., 2004; Kato et al., 2008), but are shown here to be targetable in *P. berghei* (Figures S3A and S3B). In the case of *cdpk1* our data confirm another recent study (Jebiwott et al., 2013), which also showed that the unconditional disruption of *cdpk1* has no impact on blood stage growth but reproduces the ookinete phenotype we recently described for a stage-specific mutant in the same gene (Sebastian et al., 2012). Generating and genotyping individual knockout clones for *gsk3*, *tkl3*, and PBANKA_082960, a putative protein kinase of unknown function, confirmed the barseq phenotype and established that these genes are indeed dispensable for blood stage growth (Figure S4).

We next turned to the *rio* group of protein kinases. *rio1* and *rio2* encode ancient atypical protein kinases that are highly conserved in most archaea and all eukaryotes, where they perform essential functions in ribosome biogenesis (LaRonde, 2014). In pooled transfections, barcodes for both genes were close to the detection threshold, and only the *rio1* barcode was detected consistently at different time points. We therefore classed *rio1* as targetable and *rio2* as likely essential, although the latter produced a small number of replicating barcodes in some transfections. Targeted disruption of each *rio* gene individually yielded resistant parasites after a delayed prepatency period of 10–14 days. Resistant parasites grew very slowly under drug pressure and resisted dilution cloning.

Whole-genome sequencing of a putative *rio1*^−^ population confirmed the nearly complete absence of coverage of the target gene, consistent with the uncloned population being strongly enriched in parasites carrying a correctly integrated *rio1* deletion vector (Figure 5B). Targeting of *rio2*, in contrast, selected for a 29.7 kb duplication on chromosome 5 that included the *rio2* locus, together with three other genes (Figure 5B). Read coverage suggested these parasites carried one disrupted and one intact copy of the target gene, which was consistent with PCR evidence showing the presence of both a disrupted and a wild-type *rio2* locus in the mutant population (Figure S5A) and Southern hybridization of separated chromosomes showing integration of the *rio2* targeting vector into chromosome 5, as expected (Figure S5B). These data fit a model in which a pre-existing partial genome duplication can predispose a small proportion of the parasite population to survive integration of a deletion vector for an essential gene. Partial genome duplications of a similar size exist transiently in *P. falciparum*, where they serve as starting points for the evolution of drug resistance (Guler et al., 2013).

Of six kinase genes selected for follow-up as potential false positives, only the least robustly detectable one proved to be a false hit, while the other five revealed targetable genes missed by an earlier screen (Figure 5C). From these data it is highly plausible that replicating barcodes represent correctly integrated vectors in the vast majority of cases. False positives may be due to pre-existing short genome duplications, but such events are rare. In yeast rare events are filtered effectively by disregarding data from the 10% of least abundant mutants in barseq screens (Robinson et al., 2014). Such approaches can be applied to future *P. berghei* screens to reduce the false-positive rate even further.

**Figure 6:**
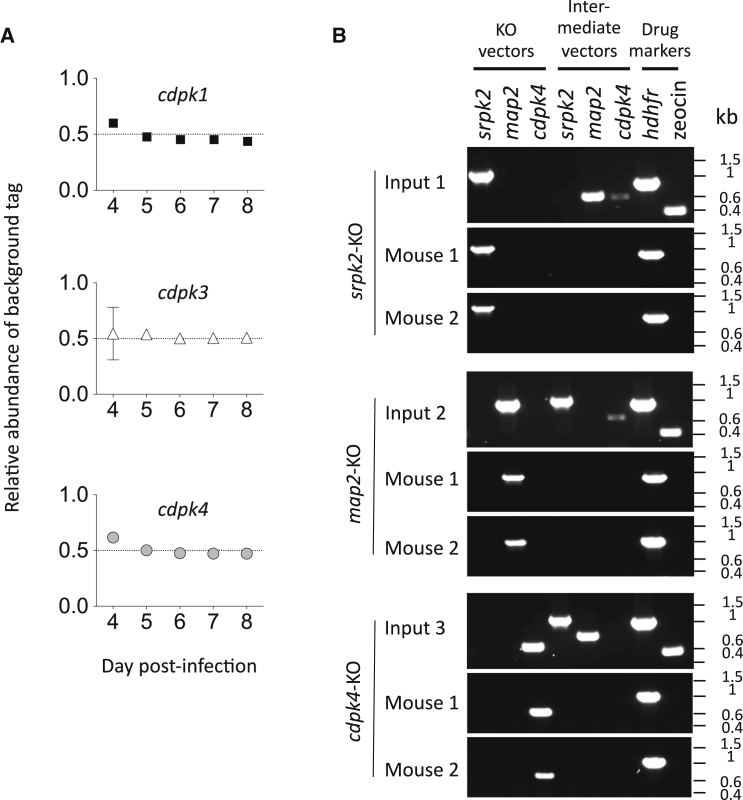
Absence of Evidence for Multiple Integration Events (A) Vector pools were transfected into marker-free lines with pre-existing barcodes in *cdpk1*, *cdpk3*, or *cdpk4*. New barcodes account for approximately half of the total, as would be expected if each parasite genome carried exactly one new barcode. The slight overrepresentation of background barcodes on day 4 probably comes from parasites that failed to integrate a vector and which were not yet completely eliminated after only 3 days of drug selection. All data points are supported by three experiments, and error bars show standard deviations. See Figure S3 for genotyping of marker-free lines. (B) PCR genotyping was performed on parasite gDNA from six infected mice, each transfected with one of three final targeting vectors in the presence of a 20-fold excess of intermediate vectors (10 μg total DNA per transfection), which have the same homology arms but a zeocin resistance cassette that cannot be selected in *P. berghei*. Presence of intermediate vectors in the input cocktail but absence in the resistant parasite populations suggests that multiple integration events are rare or absent, since hitchhiking of marker free insertions would otherwise be observed.

Finally we asked whether the interpretation of barcode counting data from parallel transfection experiments could be confounded by multiple vectors integrating into the same parasite genome. First we transfected pools of barcoded vectors into three parasite clones, each of which already carried a different barcode from the insertion of a targeting vector and subsequent recycling of the selection marker. We reasoned that if each mutant integrated one new vector, the pre-existing barcode should account for exactly half of all barcode counts after transfection and drug selection. The data were entirely consistent with this model (Figure 6A), suggesting that if multiple integration events existed, they would be too rare to be isolated by limiting dilution cloning. To detect rare double integration events, we chose three genes that are readily disrupted using *Plasmo*GEM vectors. For each we transfected the final knockout vector together with a 20-fold excess of the selection marker-free intermediate vectors for the other two genes. We expected to detect replication of the marker-free vectors by PCR, but only if their genomic integration coincided with integration of the selectable construct into the same genome. These experiments failed to generate evidence for multiple integration events into the same genome (Figure 6B). The data indicate that different homologous integration events in *P. berghei* are independent, and suggest that DNA uptake after electroporation is not the factor limiting transfection efficiency.

Taken together these data demonstrate that barcode counting provides a powerful tool to identify targetable *P. berghei* genes and to rapidly and reliably measure the competitive fitness of dozens of mutants during asexual erythrocytic growth in a way that is neither confounded by double integration events nor likely to generate an excessive number of false-positive or false-negative results.

## Discussion

The *Plasmo*GEM resource has begun to facilitate conventional gene knockout and tagging experiments in *P. berghei* by providing robust reagents for use in biological studies (see for instance Frénal et al., 2013). While our current emphasis is on generating and distributing the largest possible resource of knockout vectors, the same pipeline can be used to manufacture barcoded constructs that modify the 5′ or 3′ end of a gene. Due to the modular nature of the pipeline, the same intermediates can already be used to generate vectors for fluorescence and epitope tagging, and we anticipate that inducible systems that rely on regulatable promoters, degrons or aptamers (Armstrong and Goldberg, 2007; Goldfless et al., 2014; Pino et al., 2012) can be scaled up using the same approach. Recombinase-mediated engineering has already facilitated the generation of conditional alleles for inducible knockout approaches that rely on Flp recombinase (Suarez et al., 2013), and a similar strategy may be used for the insertion of loxP recognition sites for Cre-mediated recombination, which is becoming an important tool for generating inducible alleles in *Plasmodium* (Collins et al., 2013).

As well as being highly versatile, we demonstrate here that *Plasmo*GEM vectors can be used to perform functional screens. Three lines of evidence support our conclusion that fitness measurements based on barcode counting generally reflect homologous integration events at the target locus: (1) a barseq screen of protein kinases generated data that were in good agreement with the previous conventional knockout study by Tewari et al. (2010); (2) barcodes of essential kinase genes did not replicate in the parasite, showing that nonintegrated vectors are not maintained episomally but are effectively eliminated by day 4 of the infection; (3) unexpected mutants were in genes that we later verified as targetable by transfecting individual vectors and genotyping the resulting mutants. In one instance the inconsistent replication of a barcode raised the unexpected possibility of genetic redundancy of *rio2*, until whole-genome sequencing revealed a 29.7 kb duplication containing a second intact copy of the target gene. Whole-genome sequences of many rodent *Plasmodium* genomes are now available and contain no evidence that genome duplications commonly become fixed (Otto et al., 2014), possibly because of associated fitness costs. False positives of the *rio2* type will therefore always be rare events that can be filtered out, for instance by eliminating the least abundant 10% of mutants from the analysis, as has been recommended for barseq screens of yeast mutants (Robinson et al., 2014).

The use of vector pools to phenotype mixtures of mutants is particularly suited to *P. berghei*, where a library of cloned mutants would be of limited value, because the absence of an in vitro culture system for blood stages means that each cloned mutant has to be generated and propagated in mice. In marked contrast, the human malaria parasite *P. falciparum* has an efficient culture system for blood stages, but low recombination rates require transfection with circular vectors, and our preliminary data suggest that linear vectors of the type used here do not spontaneously integrate into the genome, even when their homology arms are each several kilobases long. The CRISPR-Cas9 system has recently been used successfully to transfect linear DNA in *P. falciparum* (Ghorbal et al., 2014). If this method increases recombination rates significantly it raises the exciting prospect of generating large numbers of barcoded *P. falciparum* mutants that could then be pooled for genetic screens using barseq screening.

The scale of barseq screens in *P. berghei* is currently constricted by the complexity of the parasite pools that can be generated, which in turn is limited both by transfection efficiency and by the relatively large differences in recombination rates of different *Plasmo*GEM vectors. We show that as a result of such heterogeneity, screening pools of mutants poses the risk of losing genotypes that are generated in small numbers due to inefficient recombination rates at a target locus, or because they have a reduced fitness. Such mutants get outcompeted by the faster-growing genotypes in the pool, but our pilot screen suggests that in many cases their presence and growth rates can be measured in a second-pass screen that excludes most of the faster-growing mutants. Once the vector-specific integration rates and fitness measurements are available for the entire vector resource, it will be possible to create more bespoke vector pools that are better matched for integration rates and fitness outcomes. This will enable the creation of larger pools of mutants, which can then be combined into even more complex superpools to empower truly genome-wide screens of parasite growth and differentiation using only a fraction of the number of rodents required today to study much smaller gene sets. We anticipate that these global genetic barseq screens will make it possible to identify systematically the parasite metabolic pathways required for replication of asexual blood stages. Dropout screens that ask which barcodes are lost from the sexual parasite stages may generate lists of candidate genes required for parasite transmission to the mosquito, and similar screens can be designed to get at genes required for virulence or for efficient replication in normocytes versus reticulocytes. In each case, vectors from the *Plasmo*GEM resource will be available for validation and follow-up investigations.

## Experimental Procedures

### Recombinase-Mediated Engineering Pipeline

The *Plasmo*GEM vector resource was created by recombinase-mediated engineering in continuous liquid culture on 96-well plates largely as described (Pfander et al., 2013), but with the following modifications. To improve genome coverage, the arrayed *P. berghei* genomic DNA library in the pJAZZ-OK linear plasmid (Lucigen) that provides the starting point for vector production was doubled in size to ∼10,000 clones. The PCR product consisting of a *zeo-pheS* marker and 50 bp primer extensions homologous to the gene of interest was purified using the High Pure 96 UF Cleanup Kit (Roche) to improve lambda Red recombination efficiency in *E. coli*. The resulting intermediate vectors were selected in liquid culture containing 30 μg/mL kanamycin and 50 μg/mL zeocin. Kanamycin selection improved the proportion of intact intermediate vectors by selecting for the short arm of the pJAZZ plasmid. After two rounds of antibiotic selection, cultures were reinoculated into fresh selective medium and incubated for no longer than 16 hr at 37°C to favor the correct recombination product. Intermediate vector DNA was obtained using a QIAGEN Plasmid Plus 96 Miniprep kit and eluted into 65 μl Tris-EDTA buffer. DNA purity proved critical for the Gateway reaction, which was set up in 20 μl using 2 μl LR clonase (Invitrogen), 100 ng Gateway entry plasmid (e.g., pR6K-attL1-3xHA-*hdhfr-yfcu*-attL2), LR clonase buffer, and 300 ng purified intermediate vector. Gateway reactions were purified using the High Pure 96 UF Cleanup Kit. Electrocompetent *E. coli* TSA were transformed and plated on YEG-Cl agar containing kanamycin, and four colonies were picked to verify the sequence of their homology arms.

### Computational Vector Design

A suite of software tools for automated vector design was created to work with the arrayed *E. coli* library of mapped *P. berghei* ANKA gDNA clones and has been described elsewhere (Schwach et al., 2015). In brief, the *Plasmo*GEM software tools select the most suitable library clone for each gene and type of modification and picks two unique 50 bp regions for lambda Red recombination, designated recUp and recDown, respectively. Together, these sites define the boundaries of the genomic region that is replaced by the selection marker cassette. A set of three PCR primers for quality-control purposes is also generated. For lambda Red recombination two highly unique 50 bp regions (*recUp* and *recDown*) were chosen on each selected library clone. In the case of knockout constructs these were designed to introduce the maximal deletion of the target gene compatible with a homology arm length of ≥1 kb, while leaving at least 1 kb upstream and 0.8 kb downstream of neighboring genes intact. For C-terminal tagging vectors *recUp* and *recDown* were chosen to delete only the stop codon from the gene of interest. *recUp* and *recDown* sequences were included in oligonucleotides for lambda Red recombination, one of which also carried a 10–11 bp gene-specific barcode assigned automatically from a list of optimized sequences, and an 18 bp constant primer annealing site to read out the barcode after integration into the *P. berghei* genome. The software also automatically designed oligonucleotides for quality control during vector production (QCR1 and QCR2) and a unique oligonucleotide annealing to the *P. berghei* genome at least 200 bp outside of the boundary of the shorter homology arm (GT) for PCR genotyping transgenic parasites. Vector designs, primer, and barcode sequences can be viewed in a searchable database at http://plasmogem.sanger.ac.uk (Schwach et al., 2015). For quality control, up to four colonies per final vector were batch sequenced on an Illumina MiSeq instrument. Sequencing libraries were made essentially as described (Quail et al., 2012).

### New Tagging and Selection Cassettes

Gateway entry sclones were created for C-terminal tagging with fluorescent proteins by replacing the default 3xHA sequence in plasmid pR6K-attL1-3xHA-hdhfr-yfcu-attL2 (Pfander et al., 2013) with GFP-mu3 (Addgene plasmid 20410) (Franke-Fayard et al., 2004), iLov (Addgene plasmid 26769) (Chapman et al., 2008), mEmerald (a kind gift from J. Liu), mCherry (from RMgmDB plasmid pL0046), and mVenus. Entry clones with R6K origins were maintained in *E. coli* PIR2 (Invitrogen).

### Parasites, Animals, Vector Preparation, and Transfection

Transgenic *P. berghei* were generated either in wild-type strain ANKA 2.34 or in a selectable marker-free reporter strain of *P. berghei* ANKA cl15cy1 expressing the mu3 variant of green fluorescent protein clone RMgm-7 (Franke-Fayard et al., 2004). Parasites were routinely propagated in 6- to 8-week-old Theiler’s original (TO) outbred mice. Schizonts for barcode counting experiments were produced in female Wistar rats (200–250 g) to achieve maximal transfection efficiency. To generate pools of mutants for phenotyping by barcode counting, equal amounts of each vector were combined and the mixture digested with NotI to release the targeting vectors from the bacterial vector arms. A total of 5-8 μl of the digested vector mix, typically containing 100 ng of DNA for each vector, was used per transfection. Experiments with single vectors used 2 μg of *Not*I-restricted DNA per transfection. *Plasmo*GEM identification numbers for vectors used in this study are listed in Table S2. Transfections were done by electroporation of purified schizonts as described (Janse et al., 2006b), with the following modifications. Parasites for the schizont culture were from female Wistar rats (200–250 g) to achieve maximal transfection efficiency and were cultured for 21 hr before schizonts were isolated on a 55% Nycodenz/PBS cushion. Isolated schizonts were washed in complete media and electroporated using the 4D Nucleofector System (Lonza) in 16-well strips according to the pulse program FI-115 (see Supplemental Information, Protocol 1, for more detail). Growth rate phenotyping of transfected parasites was done in 6- to 8-week-old Balb/c inbred mice. Rodents were from Harlan, UK. All animal research was conducted under licenses from the UK Home Office and used protocols approved by the ethics committee of the Wellcome Trust Sanger Institute.

### Growth Rate Phenotyping by Barcode Counting

Three batches of schizonts were transfected with the same vector pool and each injected intravenously into a different Balb/c mouse. Resistant parasites were selected by pyrimethamine (70 mg/L in the drinking water). Infections were monitored daily using Giemsa-stained thin blood films. A total of 30 μl of infected blood was collected from the tail at exactly the same time on days 4–8 posttransfection and diluted in 200 μl of phosphate-buffered saline. Total DNA was extracted from each sample and resuspended in water (50 μl on days 4–6 posttransfection, 100 μl for later days) using Supplemental Protocol 2. To sequence the vector-specific barcodes, 1 μl of each DNA sample served as template for a PCR reaction using Advantage 2 Taq polymerase (Clontech) with primers arg444 and arg445 (1× 95°C/5 min denaturation, 35 × 95°C/30 s, 55°C/20 s, 68°C/8 s, 1 × 10 min at 68°C), which bind to constant annealing sites flanking each barcode. The 167 bp amplicon was further extended in a second PCR reaction using oligonucleotides that in their 5′ extensions introduce Illumina adaptors and sample-specific barcodes (Table S3) for multiplexing up to 32 samples in one run of a MiSeq instrument. For sample-specific indexing, 5 μl of the first amplicon served as template for a further ten amplification cycles (1 × 95°C/2 min, 10 × 95°C/30 s, 68°C/15 s, 1 × 5 min, 68°C) using one generic oligonucleotide (PE1.0) and one of a set of 32 sample-specific indexing oligonucleotide. A total of 100 ng of each sequencing library was pooled and quality controlled by quantitative PCR for the presence of sequencing adaptors. Libraries were sequenced using MiSeq Reagent Kit v2 (300 cycle) from Illumina (MS-102-2002). In some experiments we compared PCR-mediated indexing to adaptor ligation libraries.

Due to their low complexity, PCR amplicon libraries had to be diluted to 4 nM before loading the flow cell of a MiSeq instrument (Illumina) at low cluster density (4 × 10^5^ clusters/mm^2^) with 30%–50% of PhiX spike-in. Sequencing of 150 bp paired-ends yielded 1.0–1.5 × 10^5^ reads on average for each of the 32 samples. Using a Perl script, barcode sequences were extracted from sequencer output, counted, and the relative abundance of each barcode within the pool determined. The quantitation was considered reliable for barcodes accounting for at least 0.1% of all counts. Parasitaemia curves for these mutants were inferred from the relative abundance of each barcode and from the observed total parasitaemia as determined using a Giemsa stained thin blood film. The relative fitness (*w*) of a mutant represented by a barcode on a given day (*d*) was calculated according to Mani et al. (2008) by comparing the daily change in its relative abundance (*A*) to that of the reference genes (*A*_*Ri …*_ *A*_*Rn*_) with normal growth.

$$w_{gene\;d}=\frac{A_{d}}{A_{d-1}}\times \frac{\sum_{i=1}^{n}\frac{A_{Ri\;d-1}}{A_{Ri\;d}}}{n}$$

Statistical analyses compared each barcode against the normal-growth reference vectors using a two-tailed t test (unequal variance, p values adjusted according to the false discovery rate method). A given mutant was considered viable when consistent growth of its barcode was observed for all time points in at least two of three replicates.

### Genotyping

To verify vector integration by diagnostic PCR on parasite genomic DNA, we designed a target gene-specific oligonucleotide to anneals to the chromosome just outside of the vector’s homology arm (Table S2) and paired it with a primer annealing to the *hdhfr* cassette within the targeting vector (either arg216 or arg218, depending on the orientation of the selection cassette relative to the first oligonucleotide). Integration of the targeting construct into the correct chromosome was further investigated by Southern hybridization of chromosomes separated by pulsed-field gel electrophoresis (PFGE) as described previously (Pfander et al., 2011).

## Author Contributions

O.B. and J.C.R. directed the research. E.B. led the *Plasmo*GEM resource team with additional support from K.M. The development of barcode sequencing was led by A.R.G., who also carried out the validation experiments. F.S. constructed the *Plasmo*GEM software tools and database and provided computational support for the project. G.G., B.A., C.H., and A.R.G. produced *Plasmo*GEM vectors and provided technology development. O.B., J.C.R., E.B., F.S., A.R.G., M.A.Q., and C.P. designed and analyzed experiments. O.B. and A.R.G. wrote the paper with critical input from all authors.

## Supplemental Information

Document S1. Tables S1–S3, Figures S1–S5, and Supplemental Protocols 1 and 2

Table S4. Raw Read Count Data and Analysis for a Typical Barseq Experiment; Related to Figure 3

Document S2. Article plus Supplemental Information
