# Supplementary material for: A Genome-Scale Vector Resource Enables High-Throughput Reverse Genetic Screening in a Malaria Parasite
Source: Cell Host Microbe. 2015 Mar 11;17(3):404–13. doi: 10.1016/j.chom.2015.01.014 (PMC4362957; doi:10.1016/j.chom.2015.01.014)
Supplement: Document S1. Tables S1–S3, Figures S1–S5, and Supplemental Protocols 1 and 2 [file mmc1.pdf]

Cell Host & Microbe, *17*

Supplemental Information

A Genome Scale Vector Resource Enables High-Throughput Reverse Genetic Screening in a Malaria Parasite

Ana Rita Gomes, Ellen Bushell, Frank Schwach, Gareth Girling, Burcu Anar, Michael A. Quail, Colin Herd, Claudia Pfander, Katarzyna Modrzynska, Julian C. Rayner, and Oliver Billker

Table S1. Targetability and fitness measurements for 40 eukaryotic protein kinase genes as determined by barcode sequencing, compared to data from a previous study by Tewari *et al.* (2010), which used conventional gene targeting. p values are adjusted for multiple testing. The table combines data from 5 different barseq screens. n = 6 for most fitness measurements, except for PBANKA_082960 (n = 3), *gsk3*, *kin* and PBANKA_082710 (n = 4). p. t. = post transfection. Related to Figs. 3 and 5.

				Day 5 p. t.			Day 6 p. t.			Day 7 p. t.			Day 8 p. t.				
***P. berghei* gene ID**	**Gene name**	**Tewari *et al.* 2010**	**This study**	**Fitness**	**SD**	**p**	**Fitness**	**SD**	**p**	**Fitness**	**SD**	**p**	**Fitness**	**SD**	**p**	**Average fitness, days 5-8**	**Assessment**
PBANKA_135150	*cdpk5*	Possibly essential	Possibly essential	**NA**	NA	NA	**NA**	NA	NA	**NA**	NA	NA	**NA**	NA	NA	NA	Possibly essential
PBANKA_092520	*cdpk7*	Possibly essential	Possibly essential	**NA**	NA	NA	**NA**	NA	NA	**NA**	NA	NA	**NA**	NA	NA	NA	Possibly essential
PBANKA_031140		Possibly essential	Possibly essential	**NA**	NA	NA	**NA**	NA	NA	**NA**	NA	NA	**NA**	NA	NA	NA	Possibly essential
PBANKA_083560	*pka*	Possibly essential	Possibly essential	**NA**	NA	NA	**NA**	NA	NA	**NA**	NA	NA	**NA**	NA	NA	NA	Possibly essential
PBANKA_090380		Possibly essential	Possibly essential	**NA**	NA	NA	**NA**	NA	NA	**NA**	NA	NA	**NA**	NA	NA	NA	Possibly essential
PBANKA_091210	*ck1*	Possibly essential	Possibly essential	**NA**	NA	NA	**NA**	NA	NA	**NA**	NA	NA	**NA**	NA	NA	NA	Possibly essential
PBANKA_071730	*crk-3*	Possibly essential	Possibly essential	**NA**	NA	NA	**NA**	NA	NA	**NA**	NA	NA	**NA**	NA	NA	NA	Possibly essential
PBANKA_130920	*lammer/(CLK1)*	Possibly essential	Possibly essential	**NA**	NA	NA	**NA**	NA	NA	**NA**	NA	NA	**NA**	NA	NA	NA	Possibly essential
PBANKA_093300	*prk4*	Possibly essential	Possibly essential	**NA**	NA	NA	**NA**	NA	NA	**NA**	NA	NA	**NA**	NA	NA	NA	Possibly essential
PBANKA_080800	*crk-4*	Possibly essential	Possibly essential	**NA**	NA	NA	**NA**	NA	NA	**NA**	NA	NA	**NA**	NA	NA	NA	Possibly essential
PBANKA_090110		Possibly essential	Possibly essential	**NA**	NA	NA	**NA**	NA	NA	**NA**	NA	NA	**NA**	NA	NA	NA	Possibly essential
PBANKA_093860	*ck2*	Possibly essential	Possibly essential	**NA**	NA	NA	**NA**	NA	NA	**NA**	NA	NA	**NA**	NA	NA	NA	Possibly essential
PBANKA_101090	*tkl5*	Possibly essential	Possibly essential	**NA**	NA	NA	**NA**	NA	NA	**NA**	NA	NA	**NA**	NA	NA	NA	Possibly essential
PBANKA_144300	*nek-1*	Possibly essential	Possibly essential	**NA**	NA	NA	**NA**	NA	NA	**NA**	NA	NA	**NA**	NA	NA	NA	Possibly essential
PBANKA_135090	*pk6*	Possibly essential	Possibly essential	**NA**	NA	NA	**NA**	NA	NA	**NA**	NA	NA	**NA**	NA	NA	NA	Possibly essential
PBANKA_080560		Possibly essential	Possibly essential	**NA**	NA	NA	**NA**	NA	NA	**NA**	NA	NA	**NA**	NA	NA	NA	Possibly essential
PBANKA_122500	*fikk*	Possibly essential	Possibly essential	**NA**	NA	NA	**NA**	NA	NA	**NA**	NA	NA	**NA**	NA	NA	NA	Possibly essential
PBANKA_100820	*pkg*	Possibly essential	Possibly essential	**NA**	NA	NA	**NA**	NA	NA	**NA**	NA	NA	**NA**	NA	NA	NA	Possibly essential
PBANKA_112690	*pk4*	Possibly essential	Possibly essential	**NA**	NA	NA	**NA**	NA	NA	**NA**	NA	NA	**NA**	NA	NA	NA	Possibly essential
PBANKA_052140	*rio2*	Possibly essential	Possibly essential	**NA**	NA	NA	**NA**	NA	NA	**NA**	NA	NA	**NA**	NA	NA	NA	Possibly essential†□
PBANKA_040110	*srpk*	KO confirmed	Targetable	**0.59**	*0.18*	2.5E-02	**0.65**	*0.24*	1.3E-01	**0.80**	*0.08*	7.8E-02	**0.83**	*0.15*	2.1E-01	0.72	Confirmed KO
PBANKA_040820	*cdpk3*	KO confirmed	Targetable	**1.00**	*0.09*	1.0E+00	**0.97**	*0.05*	2.2E-01	**1.01**	*0.07*	7.6E-01	**1.04**	*0.06*	2.9E-01	1.01	Confirmed KO
PBANKA_040940	*pkrp*	KO confirmed	Targetable	**0.98**	*0.21*	9.2E-01	**1.03**	*0.09*	7.4E-01	**0.98**	*0.09*	9.0E-01	**1.02**	*0.05*	8.5E-01	1.00	Confirmed KO
PBANKA_061520	*cdpk4*	KO confirmed	Targetable	**0.94**	*0.13*	5.8E-01	**0.89**	*0.10*	4.1E-02	**0.98**	*0.07*	7.4E-01	**0.81**	*0.08*	1.4E-02	0.90	Confirmed KO
PBANKA_061670	*nek-4*	KO confirmed	Targetable	**0.87**	*0.19*	2.0E-01	**1.00**	*0.16*	9.7E-01	**1.02**	*0.11*	7.4E-01	**1.02**	*0.16*	8.5E-01	0.98	Confirmed KO
PBANKA_082710		KO confirmed	Targetable	**0.38**	*0.22*	2.5E-03	**0.65**	*0.06*	1.7E-04	**0.91**	*0.08*	2.9E-02	**1.03**	*0.38*	8.4E-01	0.74	Confirmed KO
PBANKA_092550	*cdpk6*	KO confirmed	Targetable	**0.86**	*0.12*	8.9E-02	**0.90**	*0.05*	8.7E-03	**0.79**	*0.10*	8.1E-03	**0.97**	*0.12*	6.8E-01	0.88	Confirmed KO
PBANKA_093370	*map-2*	KO confirmed	Targetable	**0.99**	*0.11*	9.5E-01	**0.95**	*0.06*	1.8E-01	**1.05**	*0.06*	2.1E-01	**1.03**	*0.05*	4.2E-01	1.00	Confirmed KO
PBANKA_101330	*map-1*	KO confirmed	Targetable	**1.04**	*0.11*	5.8E-01	**0.92**	*0.04*	4.1E-03	**1.06**	*0.04*	4.1E-02	**1.04**	*0.04*	8.5E-02	1.02	Confirmed KO
PBANKA_101980	*cdlk*	KO confirmed	Targetable	**0.20**	*0.02*	5.0E-06	**0.53**	*0.13*	1.7E-02	**0.62**	*0.42*	4.2E-01	**1.13**	*0.19*	8.0E-01	0.62	Confirmed KO
PBANKA_112270	*tkl4*	KO confirmed	Targetable	**0.46**	*0.33*	7.8E-02	**0.28**	*0.11*	2.9E-03	**0.55**	*0.16*	3.0E-02	**0.69**	*0.11*	6.2E-02	0.49	Confirmed KO
PBANKA_130690	*srpk2*	KO confirmed	Targetable	**0.83**	*0.32*	5.3E-01	**1.00**	*0.09*	9.7E-01	**1.01**	*0.14*	9.5E-01	**1.05**	*0.09*	3.9E-01	0.97	Confirmed KO
PBANKA_131800	*kin*	KO confirmed	Targetable	**1.08**	*0.29*	6.9E-01	**0.99**	*0.10*	1.9E-01	**0.97**	*0.07*	2.0E-01	**1.00**	*0.11*	8.4E-01	1.01	Confirmed KO
PBANKA_135260		KO confirmed	Targetable	**0.88**	*0.27*	5.8E-01	**0.98**	*0.07*	5.6E-01	**1.00**	*0.09*	9.5E-01	**1.03**	*0.04*	3.9E-01	0.97	Confirmed KO
PBANKA_146050	*gak*	KO confirmed	Targetable	**0.83**	*0.12*	8.9E-02	**0.74**	*0.09*	2.6E-03	**0.83**	*0.05*	1.4E-04	**1.17**	*0.11*	8.5E-02	0.89	Confirmed KO
PBANKA_030850	*tkl1*	Possibly essential	Targetable	**0.86**	*0.28*	5.3E-01	**0.96**	*0.21*	8.4E-01	**1.14**	*0.18*	1.7E-01	**1.04**	*0.06*	3.9E-01	1.00	New KO
PBANKA_031420	*cdpk1*	Possibly essential	Targetable §	**0.95**	*0.14*	5.5E-01	**0.89**	*0.05*	1.4E-03	**1.10**	*0.05*	2.7E-03	**1.00**	*0.06*	9.5E-01	0.98	New KO Ω
PBANKA_041040	*gsk-3*	Possibly essential	Targetable	**0.99**	*0.03*	8.2E-01	**1.00**	*0.04*	9.3E-01	**1.05**	*0.05*	5.6E-01	**1.01**	*0.03*	8.5E-01	1.01	New KO Ω
PBANKA_082960		Possibly essential	Targetable	**1.03**	*0.04*	7.5E-01	**1.02**	*0.08*	7.5E-01	**1.01**	*0.06*	7.5E-01	**1.05**	*0.02*	2.5E-02	1.03	New KO Ω
PBANKA_130520		Possibly essential	Targetable	**1.02**	*0.12*	9.5E-01	**0.98**	*0.13*	8.4E-01	**1.01**	*0.10*	8.3E-01	**0.99**	*0.08*	9.5E-01	1.00	New KO *
PBANKA_136210	*tkl3*	Possibly essential	Targetable	**1.05**	*0.22*	7.7E-01	**0.99**	*0.09*	9.6E-01	**1.06**	*0.14*	4.6E-01	**0.92**	*0.09*	2.0E-01	1.01	New KO Ω
PBANKA_141450		Possibly essential	Targetable	**0.91**	*0.33*	7.3E-01	**1.00**	*0.16*	9.7E-01	**0.94**	*0.07*	1.7E-01	**0.99**	*0.10*	8.5E-01	0.96	New KO *
PBANKA_142160		Possibly essential	Targetable	**1.01**	*0.13*	1.0E+00	**0.93**	*0.07*	3.4E-02	**1.10**	*0.16*	2.4E-01	**1.01**	*0.12*	9.5E-01	1.01	New KO *
PBANKA_144560	*rio1*	Possibly essential	Targetable	**0.23**	*0.00*	2.0E-03	**0.67**	*0.04*	1.1E-02	**0.91**	*0.08*	2.7E-01	**0.94**	*0.01*	5.9E-02	0.69	New KO ◊†
PBANKA_020580	*eik2; uis1*	KO confirmed	No integration	**NA**	*NA*	NA	**NA**	*NA*	NA	**NA**	*NA*	NA	**NA**	*NA*	NA	NA	False negative
PBANKA_031030	*pk7*	KO confirmed	No integration	**NA**	*NA*	NA	**NA**	*NA*	NA	**NA**	*NA*	NA	**NA**	*NA*	NA	NA	False negative
PBANKA_051490	*p28*		Targetable	**0.92**	*0.15*	5.3E-01	**0.98**	*0.10*	9.2E-01	**0.98**	*0.05*	7.4E-01	**1.01**	*0.06*	8.5E-01	0.97	Normal growth reference
PBANKA_051500	*p25*		Targetable	**1.01**	*0.12*	9.8E-01	**1.03**	*0.02*	1.0E-01	**1.04**	*0.07*	2.4E-01	**0.98**	*0.04*	3.9E-01	1.02	Normal growth reference
PBANKA_103780	*soap*		Targetable	**1.00**	*0.08*	1.0E+00	**0.95**	*0.07*	1.1E-01	**1.06**	*0.05*	1.1E-01	**1.03**	*0.05*	3.8E-01	1.01	Normal growth reference
PBANKA_030600	*p230p*		Taggable	**1.07**	*0.12*	5.8E-01	**1.04**	*0.07*	4.7E-01	**0.91**	*0.03*	8.1E-03	**0.98**	*0.09*	8.5E-01	1.00	Normal growth reference
PBANKA_140160			Targetable	**0.51**	*0.12*	1.1E-04	**0.46**	*0.06*	1.3E-05	**0.58**	*0.07*	2.1E-05	**0.87**	*0.07*	1.4E-02	0.60	Attenuated reference
PBANKA_110420	*bckdh e1b*		Targetable	**0.74**	*0.12*	6.9E-03	**0.58**	*0.07*	1.3E-05	**0.67**	*0.05*	2.8E-06	**0.61**	*0.06*	6.6E-05	0.65	Attenuated reference
PBANKA_103440	*pm4*		Targetable	**0.79**	*0.20*	8.9E-02	**0.73**	*0.05*	1.3E-05	**0.71**	*0.12*	4.3E-03	**0.70**	*0.08*	2.2E-03	0.73	Attenuated reference

* PCR genotyping evidence for targetability but no independent clone generated.
§ This study and Jebiwott *et al.*, 2013
† Confirmed by WGS of uncloned population
□ Confirmed by selection for target duplication
Ω Confirmed by genotyped clone
◊ Confirmed by PFGE analysis of uncloned population

Table S2. Gene identification numbers (ID), *Plasmo*GEM vector IDs and gene specific genotyping primers. Related to Figs. 3-4 and Fig. S1.

Gene ID	*Plasmo*GEM ID	Gene name	Gene specific primer	Sequence	Pairs with
p230p-tag	PbGEM-226060	p230p-tag	arg00448	GGAACAATATGGCTGTTCAATG	arg00218
PBANKA_051490	PbGEM-015545	28 kDa ookinete surface protein (P28)	arg00447	GGATTCCGTGAATGATCCCC	arg00216
PBANKA_051500	PbGEM-015561	25 kDa ookinete surface antigen precursor (P25)	arg00449	TGTTCCCGTTGTAACAGTGCA	arg00216
PBANKA_103780	PbGEM-097822	secreted ookinete adhesive protein (SOAP)	arg00446	TTTCCCACTGCGTACCCTTT	arg00218
PBANKA_103440	PbGEM-039254	plasmepsin IV (PM4)	arg00452	AGACAAACTTTGCCCACAACA	arg00216
PBANKA_110420	PbGEM-122074	3-methyl-2-oxobutanoate dehydrogenase (lipoamide), putative	arg00451	AAAGCCAGAAACGACATGAA	arg00218
PBANKA_140160	PbGEM-062476	methyl transferase-like protein, putative	arg00450	CATGGCTATGACCGACAGAG	arg00218
PBANKA_020580	PbGEM-082161	serine/threonine protein kinase, putative (IK2)	arg00471	CGAAGCGCTTTACCATGTGGG	arg00218
PBANKA_030850	PbGEM-009884	protein kinase, putative (TKL1)	arg00472	AGTGCATACGCTTCATGACGCT	arg00216
PBANKA_031030	PbGEM-072470	protein kinase 7 (PK7)	arg00473	AACCGAAGTGCTCTTTGCGA	arg00216
PBANKA_031140	PbGEM-111762	serine/threonine protein kinase, putative	arg00474	TGCTACCTTACGCATTGGACA	arg00218
PBANKA_031420	PbGEM-010677	calcium dependent protein kinase 1 (CDPK1)	arg00371	CCGGTATTTATCATCCAAGAG	arg00216
PBANKA_040110	PbGEM-084034	serine/threonine protein kinase, putative (SRPK1)	arg00475	TGATGCGGATTTGTGTGTGT	arg00218
PBANKA_040820	PbGEM-111826	calcium dependent protein kinase 3 (CDPK3)	arg00386	GGTTATCTATACATTTATTTGTG	arg00218
PBANKA_040940	PbGEM-111754	protein kinase, putative (PKRP)	arg00476	AGCAATGATGTAGGATGTGCA	arg00216
PBANKA_052140	PbGEM-072474	RIO-like serine/threonine kinase, putative	arg00477	TCCAAAGCGTTATGCCAAGTGT	arg00218
PBANKA_061520	PbGEM-087803	calcium dependent protein kinase 4 (CDPK4)	arg00255	GGGGGTTTGTGTGGAGGCG	arg00216
PBANKA_061670	PbGEM-111690	NIMA related kinase 4 (NEK4)	arg00234	GCACACTCACCTGAAATGTCG	arg00216
PBANKA_071730	PbGEM-072538	cdc2-related protein kinase 3 (CRK3)	arg00478	TGGTTCAATTGTTGAGCAAAGT	arg00218
PBANKA_080560	PbGEM-072522	O-sialoglycoprotein endopeptidase, putative	arg00479	TCGGAAAAACCTTGAAAGCGC	arg00218
PBANKA_080800	PbGEM-111786	cdc2-related protein kinase 4 (CRK4)	arg00480	TCGCTAGTTATATATGCTCACGC	arg00216
PBANKA_083560	PbGEM-028140	cAMP-dependent protein kinase catalytic subunit (PKAc)	arg00482	TCAAGTGAAACGGAATAGAAG	arg00218
PBANKA_090110	PbGEM-111746	protein kinase, putative	arg00483	TCAGAAAGGTATACGTCAACG	arg00216
PBANKA_090380	PbGEM-111794	serine/threonine protein kinase, putative	arg00484	AGCTTGTATGTCGATTCGAGA	arg00218
PBANKA_091210	PbGEM-111738	casein kinase 1 (CK1)	arg00485	ACGATGTGTGCAGCAGGTCT	arg00216
PBANKA_092520	PbGEM-093973	calcium-dependent protein kinase 7 (CDPK7)	arg00486	TGTCTCCCTAAAAGGCATGTGC	arg00218
PBANKA_092550	PbGEM-111850	calcium dependent protein kinase 6 (CDPK6)	arg00381	TGCACTTCACCAAAAGCGCCA	arg00218
PBANKA_093300	PbGEM-072518	serine/threonine protein kinase, puative	arg00487	AGCAGTGCACACAAAAGAAGA	arg00218
PBANKA_093370	PbGEM-111778	mitogen-activated protein kinase 2 (MAP2)	arg00256	ACCATGAGTGCATGCATAGGA	arg00216
PBANKA_101330	PbGEM-036210	mitogen-activated protein kinase 1 (MAP1)	arg00453	CGCGTGGAAAACTGTGGGC	arg00216
PBANKA_101980	PbGEM-111858	serine/threonine protein kinase, putative	arg00488	TGCCCGGAATGCACATATGTTG	arg00216
PBANKA_112270	PbGEM-111714	protein kinase, putative (TKL4)	arg00489	TGGGGAGTACCTTGCCCATGCA	arg00216
PBANKA_112690	PbGEM-099789	protein kinase PK4 (PK4)	arg00490	AGTATTGCCCCATCCATTGCT	arg00218
PBANKA_122500	PbGEM-111674	serine/threonine protein kinase, FIKK family	arg00491	TGTCTGACTCTCCATGGTGTCC	arg00218
PBANKA_130520	PbGEM-053796	serine/threonine protein kinase, putative	arg00456	GAGTACCTGTTGGTCACGC	arg00216
PBANKA_130690	PbGEM-104812	serine/threonine protein kinase, putative (SRPK2)	arg00225	TGCCCTTTTGATGCCAAGACG	arg00216
PBANKA_130920	PbGEM-104970	serine/threonine kinase-1, putative	arg00492	TCACGCATCGGGGATTTGTCA	arg00216
PBANKA_131800	PbGEM-105530	serine/threonine protein kinase, putative	arg00493	ACGGAGCACAATGTATGCCATG	arg00218
PBANKA_135150	PbGEM-111682	calcium dependent protein kinase 5 (CDPK5)	arg00494	TCGACGGTACTGTCTGACTGGT	arg00216
PBANKA_135260	PbGEM-111802	serine/threonine protein kinase, putative	arg00259	CTGGCGCACGGCAAAACCC	arg00218
PBANKA_136210	PbGEM-108848	protein kinase, putative	arg00262	ACGACAATGTGCATGCCTCA	arg00218
PBANKA_141450	PbGEM-111842	protein kinase, putative	arg00272	CCACAAAGCAATTCCGGTGC	arg00218
PBANKA_142160	PbGEM-065291	calcium/calmodulin-dependent protein kinase, putative	arg00454	TCTAAATCGCGGCTTTCACA	arg00218
PBANKA_144560	PbGEM-111706	protein kinase, putative	arg00495	TGCTCAAGCAACAGCAGGACA	arg00216
PBANKA_146050	PbGEM-072542	serine/threonine protein kinase, putative	arg00173	CCTGGAATTGTTTCCCCACAC	arg00218

Table S3. Oligonucleotide used for barcode sequencing. Related to Fig. 3 and S1.

Name	Sequence
arg00444	TCGGCATTCCTGCTGAACCGCTCTTCCGATCTGTAATTCGTGCGCGTCAG
arg00445	ACACTCTTTCCCTACACGACGCTCTTCCGATCTCCTTCAATTTCGATGGGTAC
PE1.0	AATGATACGGCGACCACCGAGATCTACACTCTTTCCCTACACGACGCTCTTCCGATC*T 1)
iPCRindex1	CAAGCAGAAGACGGCATACGAGATTGCTAATCACTGAGATCGGTCTCGGCATTCCTGCTGAACCGCTCTTCCGATC*T
iPCRindex2	CAAGCAGAAGACGGCATACGAGATTAGGGGGATTCGAGATCGGTCTCGGCATTCCTGCTGAACCGCTCTTCCGATC*T
iPCRindex3	CAAGCAGAAGACGGCATACGAGATAGTTTCCCAGGGAGATCGGTCTCGGCATTCCTGCTGAACCGCTCTTCCGATC*T
iPCRindex4	CAAGCAGAAGACGGCATACGAGATCCTGGGAGGTAGAGATCGGTCTCGGCATTCCTGCTGAACCGCTCTTCCGATC*T
iPCRindex5	CAAGCAGAAGACGGCATACGAGATATACCACAAATGAGATCGGTCTCGGCATTCCTGCTGAACCGCTCTTCCGATC*T
iPCRindex6	CAAGCAGAAGACGGCATACGAGATGATCTCTCGGGGAGATCGGTCTCGGCATTCCTGCTGAACCGCTCTTCCGATC*T
iPCRindex7	CAAGCAGAAGACGGCATACGAGATACCCTATACTCGAGATCGGTCTCGGCATTCCTGCTGAACCGCTCTTCCGATC*T
iPCRindex8	CAAGCAGAAGACGGCATACGAGATCTCAATTAAGAGAGATCGGTCTCGGCATTCCTGCTGAACCGCTCTTCCGATC*T
iPCRindex9	CAAGCAGAAGACGGCATACGAGATCGACAGAACGTGAGATCGGTCTCGGCATTCCTGCTGAACCGCTCTTCCGATC*T
iPCRindex10	CAAGCAGAAGACGGCATACGAGATTCGCCATTATGGAGATCGGTCTCGGCATTCCTGCTGAACCGCTCTTCCGATC*T
iPCRindex11	CAAGCAGAAGACGGCATACGAGATATGTTCCGGCCGAGATCGGTCTCGGCATTCCTGCTGAACCGCTCTTCCGATC*T
iPCRindex12	CAAGCAGAAGACGGCATACGAGATTCTTGAAGTGAGAGATCGGTCTCGGCATTCCTGCTGAACCGCTCTTCCGATC*T
iPCRindex13	CAAGCAGAAGACGGCATACGAGATGAAGGCCAGCTGAGATCGGTCTCGGCATTCCTGCTGAACCGCTCTTCCGATC*T
iPCRindex14	CAAGCAGAAGACGGCATACGAGATCCAATGTGCAGGAGATCGGTCTCGGCATTCCTGCTGAACCGCTCTTCCGATC*T
iPCRindex15	CAAGCAGAAGACGGCATACGAGATATCGAAGGACCGAGATCGGTCTCGGCATTCCTGCTGAACCGCTCTTCCGATC*T
iPCRindex16	CAAGCAGAAGACGGCATACGAGATTCGGGTGCGAAGAGATCGGTCTCGGCATTCCTGCTGAACCGCTCTTCCGATC*T
iPCRindex17	CAAGCAGAAGACGGCATACGAGATGTAATTTACGGGAGATCGGTCTCGGCATTCCTGCTGAACCGCTCTTCCGATC*T
iPCRindex18	CAAGCAGAAGACGGCATACGAGATATATCGACTACGAGATCGGTCTCGGCATTCCTGCTGAACCGCTCTTCCGATC*T
iPCRindex19	CAAGCAGAAGACGGCATACGAGATTGATTCTTACAGAGATCGGTCTCGGCATTCCTGCTGAACCGCTCTTCCGATC*T
iPCRindex20	CAAGCAGAAGACGGCATACGAGATACGGCGGGCCTGAGATCGGTCTCGGCATTCCTGCTGAACCGCTCTTCCGATC*T
iPCRindex21	CAAGCAGAAGACGGCATACGAGATCTTGCGTGGAGGAGATCGGTCTCGGCATTCCTGCTGAACCGCTCTTCCGATC*T
iPCRindex22	CAAGCAGAAGACGGCATACGAGATTAATCAAAGACGAGATCGGTCTCGGCATTCCTGCTGAACCGCTCTTCCGATC*T
iPCRindex23	CAAGCAGAAGACGGCATACGAGATGGCGGGCTCTAGAGATCGGTCTCGGCATTCCTGCTGAACCGCTCTTCCGATC*T
iPCRindex24	CAAGCAGAAGACGGCATACGAGATCCTCCATTTCTGAGATCGGTCTCGGCATTCCTGCTGAACCGCTCTTCCGATC*T
iPCRindex25	CAAGCAGAAGACGGCATACGAGATAACCAGCGCTGGAGATCGGTCTCGGCATTCCTGCTGAACCGCTCTTCCGATC*T
iPCRindex26	CAAGCAGAAGACGGCATACGAGATTATTCGTCAACGAGATCGGTCTCGGCATTCCTGCTGAACCGCTCTTCCGATC*T
iPCRindex27	CAAGCAGAAGACGGCATACGAGATGCGCTGATGCAGAGATCGGTCTCGGCATTCCTGCTGAACCGCTCTTCCGATC*T
iPCRindex28	CAAGCAGAAGACGGCATACGAGATCTCATATGGCTGAGATCGGTCTCGGCATTCCTGCTGAACCGCTCTTCCGATC*T
iPCRindex29	CAAGCAGAAGACGGCATACGAGATACAGGGGCAGGGAGATCGGTCTCGGCATTCCTGCTGAACCGCTCTTCCGATC*T
iPCRindex30	CAAGCAGAAGACGGCATACGAGATGGTTTTATACCGAGATCGGTCTCGGCATTCCTGCTGAACCGCTCTTCCGATC*T
iPCRindex31	CAAGCAGAAGACGGCATACGAGATGCATGACTTTAGAGATCGGTCTCGGCATTCCTGCTGAACCGCTCTTCCGATC*T
iPCRindex32	CAAGCAGAAGACGGCATACGAGATTTCTGAGTTCTGAGATCGGTCTCGGCATTCCTGCTGAACCGCTCTTCCGATC*T

1) © 2006-2008 Illumina, Inc. All rights reserved

Figure S1

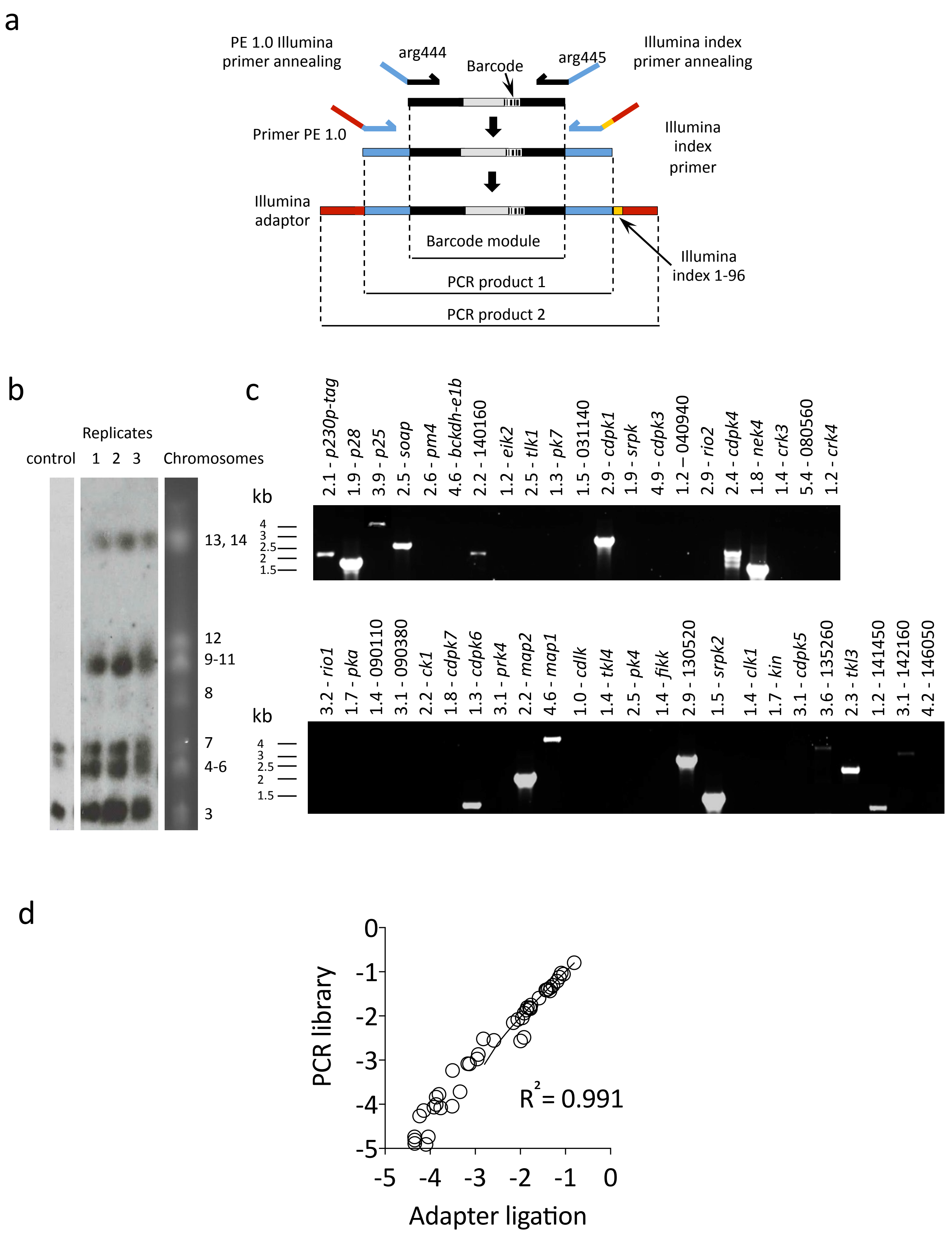

a
PE 1.0 Illumina primer annealing
arg444
Barcode
arg445
Illumina index primer annealing
Primer PE 1.0
Illumina index primer
Illumina adaptor
Barcode module
PCR product 1
PCR product 2
Illumina index 1-96
b
Replicates
control 1 2 3
Chromosomes
13, 14
12
9-11
8
7
4-6
3
c
kb
4
3
2.5
2
1.5
2.1 - p230p-tag
1.9 - p28
3.9 - p25
2.5 - soap
2.6 - pm4
4.6 - bckdh-e1b
2.2 - 140160
1.2 - eik2
2.5 - tlk1
1.3 - pk7
1.5 - 031140
2.9 - cdpk1
1.9 - srpk
4.9 - cdpk3
1.2 – 040940
2.9 - rio2
2.4 - cdpk4
1.8 - nek4
1.4 - crk3
5.4 - 080560
1.2 - crk4
3.2 - rio1
1.7 - pka
1.4 - 090110
3.1 - 090380
2.2 - ck1
1.8 - cdpk7
1.3 - cdpk6
3.1 - prk4
2.2 - map2
4.6 - map1
1.0 - cdlk
1.4 - tkl4
2.5 - pk4
1.4 - fikk
2.9 - 130520
1.5 - srpk2
1.4 - clk1
1.7 - kin
3.1 - cdpk5
3.6 - 135260
2.3 - tkl3
1.2 - 141450
3.1 - 142160
4.2 - 146050
d
PCR library
Adapter ligation
R2 = 0.991
0
-1
-2
-3
-4
-5

Figure S2

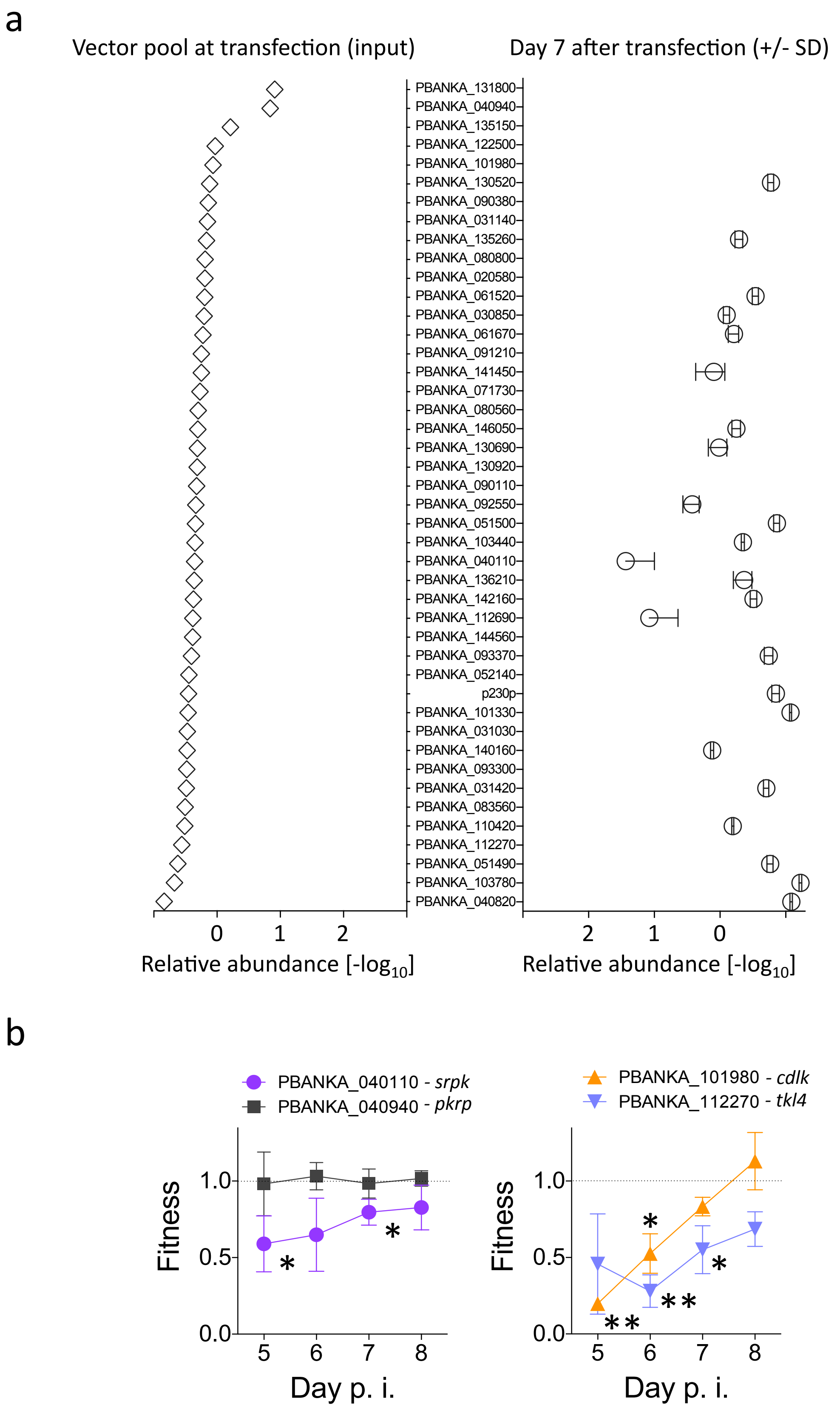
a
Vector pool at transfection (input)
Day 7 after transfection (+/- SD)
PBANKA_131800
PBANKA_040940
PBANKA_135150
PBANKA_122500
PBANKA_101980
PBANKA_130520
PBANKA_090380
PBANKA_031140
PBANKA_135260
PBANKA_080800
PBANKA_020580
PBANKA_061520
PBANKA_030850
PBANKA_061670
PBANKA_091210
PBANKA_141450
PBANKA_071730
PBANKA_080560
PBANKA_146050
PBANKA_130690
PBANKA_130920
PBANKA_090110
PBANKA_092550
PBANKA_051500
PBANKA_103440
PBANKA_040110
PBANKA_136210
PBANKA_142160
PBANKA_112690
PBANKA_144560
PBANKA_093370
PBANKA_052140
p230p
PBANKA_101330
PBANKA_031030
PBANKA_140160
PBANKA_093300
PBANKA_031420
PBANKA_083560
PBANKA_110420
PBANKA_112270
PBANKA_051490
PBANKA_103780
PBANKA_040820
0 1 2
2 1 0
Relative abundance [-log10]
Relative abundance [-log10]
b
PBANKA_040110 - srpk
PBANKA_040940 - pkrp
PBANKA_101980 - cdlk
PBANKA_112270 - tkl4
Fitness
1.0
0.5
0.0
5 6 7 8
Day p. i.

Figure S3

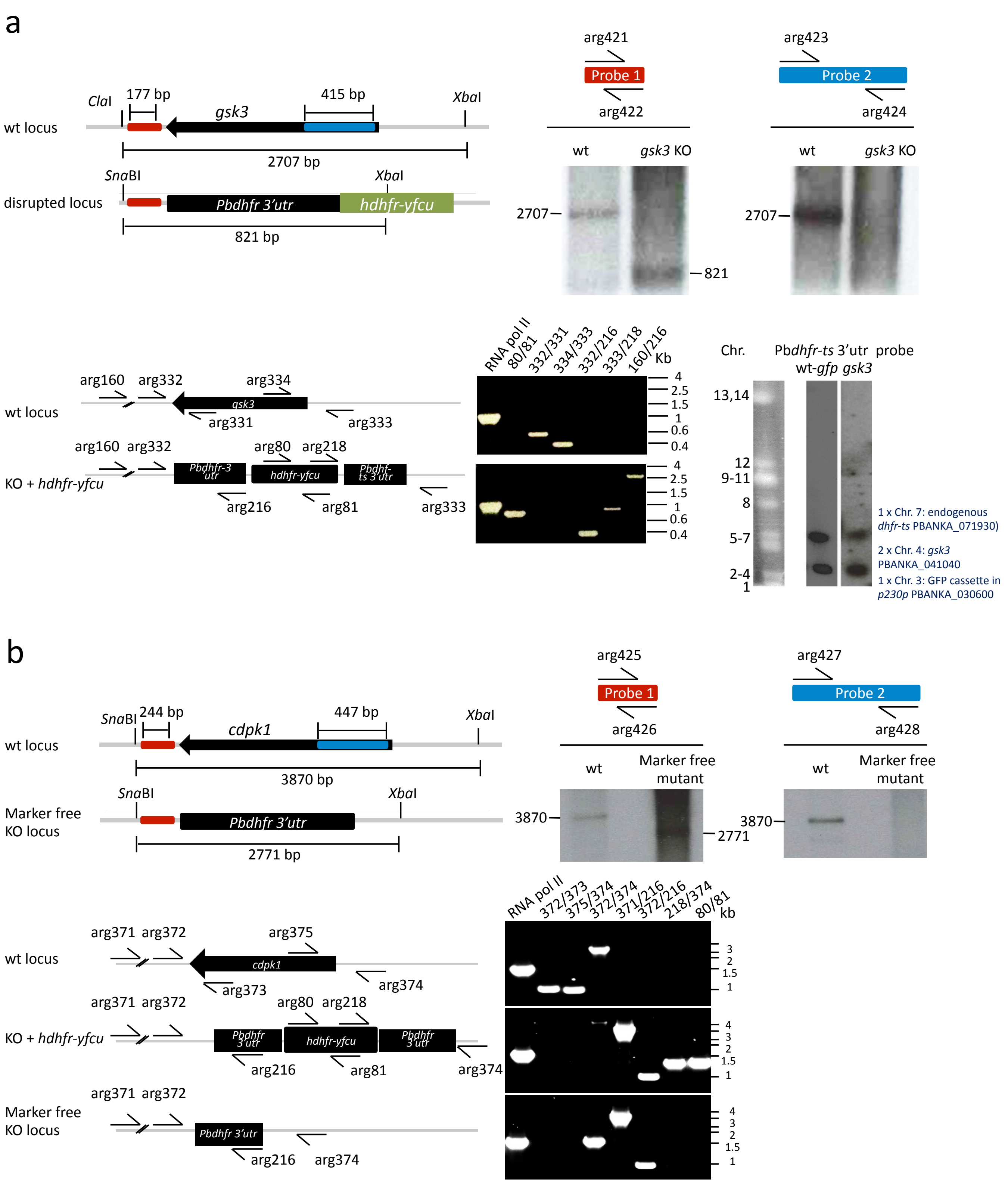
a
ClaI
177 bp
415 bp
XbaI
gsk3
wt locus
2707 bp
SnaBI
XbaI
disrupted locus
Pbdhfr 3'utr
hdhfr-yfcu
821 bp
arg421
Probe 1
arg422
wt
gsk3 KO
2707
821
arg423
Probe 2
arg424
wt
gsk3 KO
2707
arg160
arg332
arg334
wt locus
gsk3
arg331
arg333
arg160
arg332
arg80
arg218
KO + hdhfr-yfcu
Pbdhfr-3 'utr
hdhfr-yfcu
Pbdhf-ts 3'utr
arg216
arg81
arg333
RNA pol II
80/81
332/331
334/333
332/216
333/218
160/216
Kb
4
2.5
1.5
1
0.6
0.4
Chr.
Pbdhfr-ts 3'utr probe
wt-gfp gsk3
13,14
12
9-11
8
5-7
2-4
1
1 x Chr. 7: endogenous dhfr-ts PBANKA_071930)
2 x Chr. 4: gsk3 PBANKA_041040
1 x Chr. 3: GFP cassette in p230p PBANKA_030600
b
SnaBI
244 bp
447 bp
XbaI
cdpk1
wt locus
3870 bp
SnaBI
XbaI
Marker free KO locus
Pbdhfr 3'utr
2771 bp
arg425
Probe 1
arg426
wt
Marker free mutant
3870
2771
arg427
Probe 2
arg428
wt
Marker free mutant
3870
arg371
arg372
arg375
wt locus
cdpk1
arg373
arg374
arg371
arg372
arg80
arg218
KO + hdhfr-yfcu
Pbdhfr 3'utr
hdhfr-yfcu
Pbdhfr 3'utr
arg216
arg81
arg374
arg371
arg372
Marker free KO locus
Pbdhfr 3'utr
arg216
arg374
RNA pol II
372/373
375/374
372/374
371/216
372/216
218/374
80/81
kb
3
2
1.5
1
4

Figure S4

a

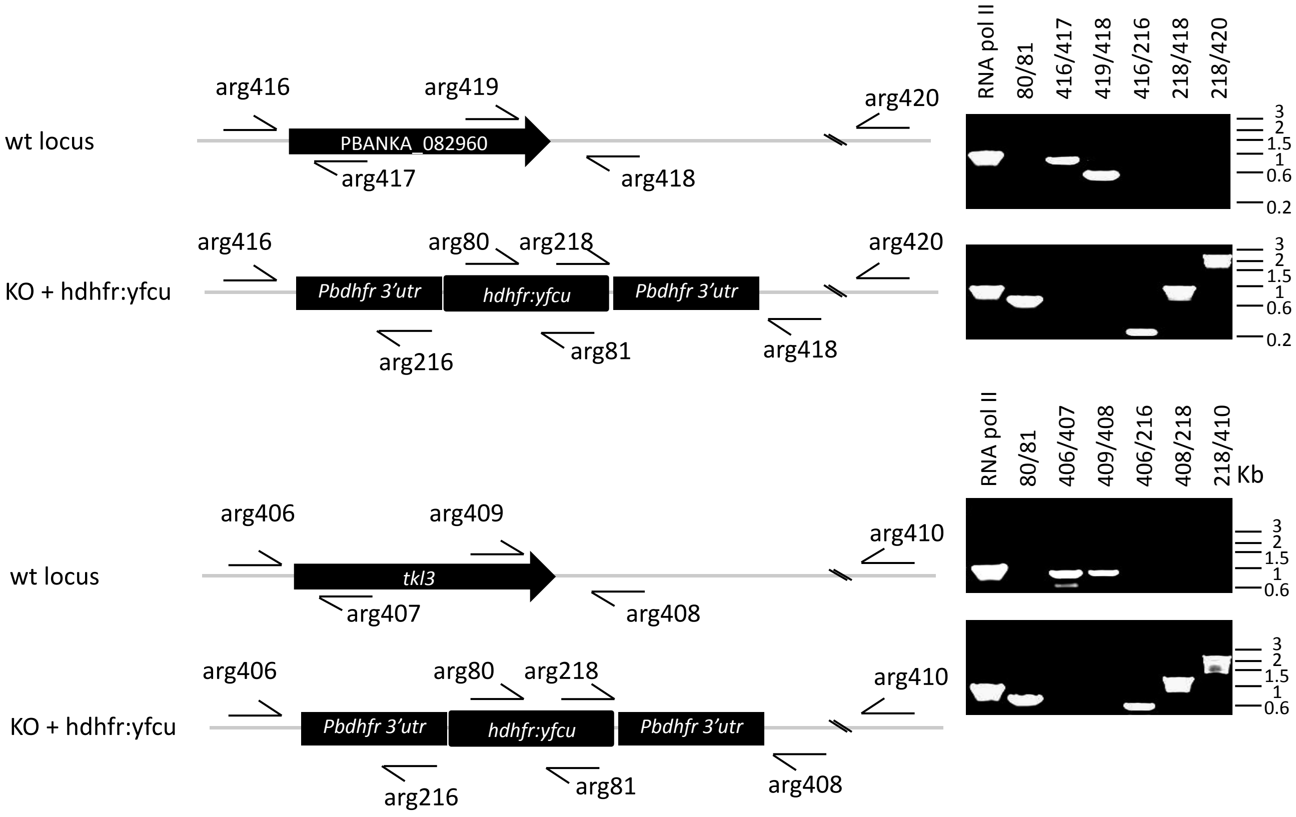
arg416
arg419
arg420
wt locus
PBANKA_082960
arg417
arg418
arg416
arg80
arg218
arg420
KO + hdhfr:yfcu
Pbdhfr 3'utr
hdhfr:yfcu
Pbdhfr 3'utr
arg216
arg81
arg418
RNA pol II
80/81
416/417
419/418
416/216
218/418
218/420
3
2
1.5
1
0.6
0.2
arg406
arg409
arg410
wt locus
tkl3
arg407
arg408
arg406
arg80
arg218
arg410
KO + hdhfr:yfcu
Pbdhfr 3'utr
hdhfr:yfcu
Pbdhfr 3'utr
arg216
arg81
arg408
RNA pol II
80/81
406/407
409/408
406/216
408/218
218/410
Kb

b

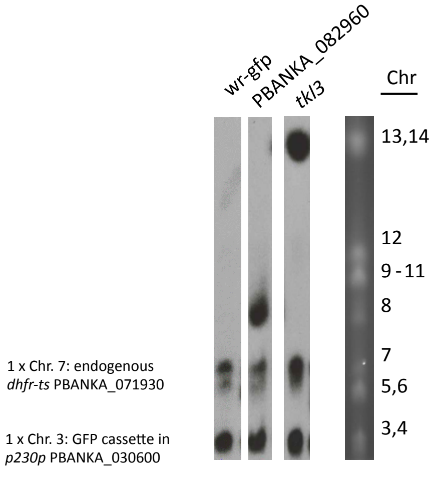
wr-gfp
PBANKA_082960
tkl3
Chr
13,14
12
9 - 11
8
7
5,6
3,4
1 x Chr. 7: endogenous
dhfr-ts PBANKA_071930
1 x Chr. 3: GFP cassette in
p230p PBANKA_030600

Figure S5

a

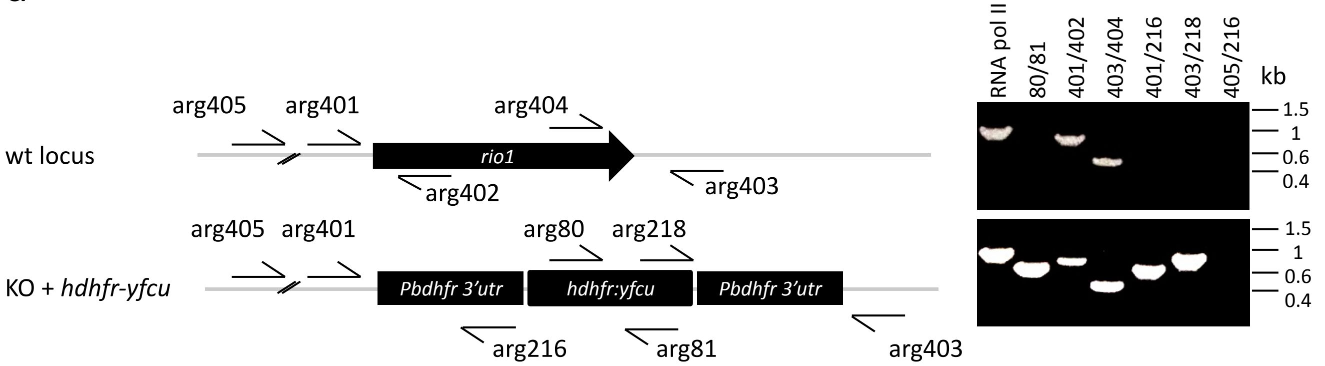

arg405
arg401
arg404
wt locus
rio1
arg402
arg403
arg405
arg401
arg80
arg218
KO + hdhfr-yfcu
Pbdhfr 3'utr
hdhfr:yfcu
Pbdhfr 3'utr
arg216
arg81
arg403
RNA pol II
80/81
401/402
403/404
401/216
403/218
405/216
kb
1.5
1
0.6
0.4
1.5
1
0.6
0.4

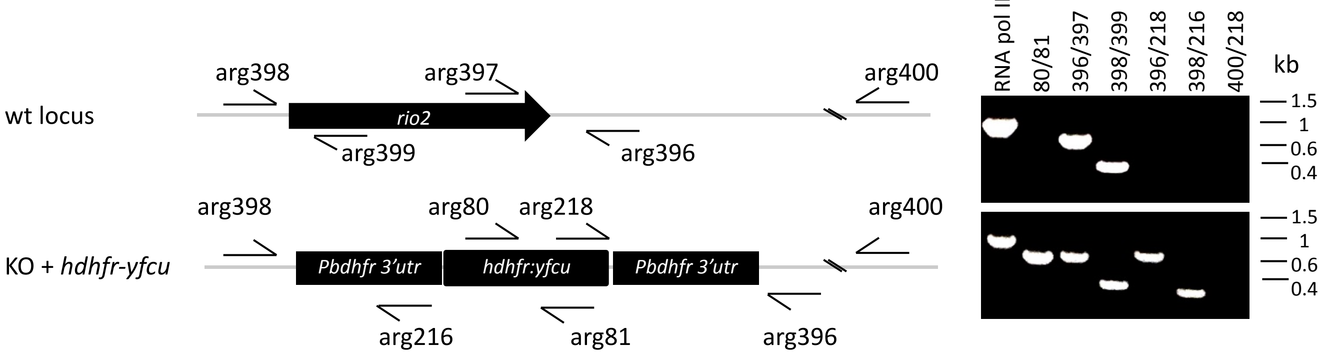

arg398
arg397
arg400
wt locus
rio2
arg399
arg396
arg398
arg80
arg218
arg400
KO + hdhfr-yfcu
Pbdhfr 3'utr
hdhfr:yfcu
Pbdhfr 3'utr
arg216
arg81
arg396
RNA pol II
80/81
396/397
398/399
396/218
398/216
400/218
kb
1.5
1
0.6
0.4
1.5
1
0.6
0.4

b

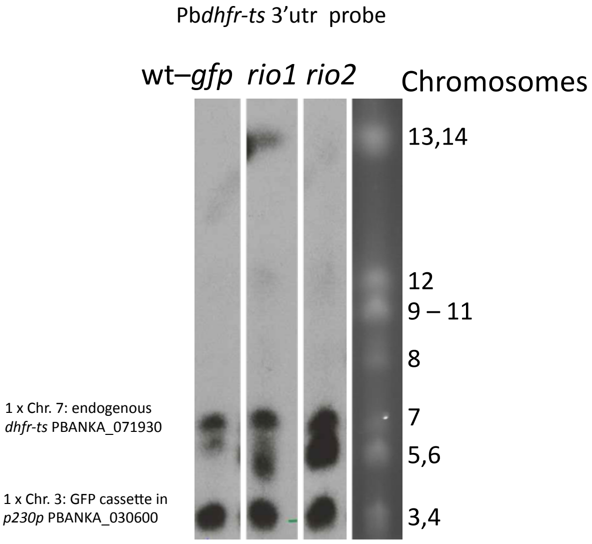

Pbdhfr-ts 3'utr probe
wt–gfp rio1 rio2
Chromosomes
13,14
12
9 – 11
8
7
5,6
3,4
1 x Chr. 7: endogenous dhfr-ts PBANKA_071930
1 x Chr. 3: GFP cassette in p230p PBANKA_030600

SUPPLEMENTAL FIGURE TITLES AND LEGENDS

Figure S1. Validation of barcode sequencing to phenotype multiple *P. berghei* mutants in the same mouse. Related to Fig. 2 and Fig. 3.

a. Schematic of the barcode module illustrating primer annealing sites and amplification steps. See supplemental information for oligonucleotide sequences.

b. Southern hybridization of chromosomes separated by pulsed-field gel electrophoresis with a ~500 bp probe corresponding to the *dhfr-ts* 3' UTR that is present as two copies in each *Plasmo*GEM vector. Samples were from day 8 post transfection of the experiment shown in Fig. 2b (left panel). In the background strain the probe hybridizes to the *dhfr-ts* locus in chromosome 7, which appears to be polymorphic and to a GFP cassette integrated in the p230p locus of chromosome 3.

c. Agarose gel electrophoresis of PCR products across the predicted integration sites from the same experiment as **b**, supporting genomic integration for 17 of the 22 vectors whose barcodes are detected in Fig. 2b. Oligonucleotides were designed to amplify from the selection cassette (arg218 or arg216) across the shorter homology arm into the flanking genomic sequence. For each gene the predicted product size is stated in kb before the gene name. Note that the long homology arms of *Plasmo*GEM vectors can give rise to false negative results from PCR genotyping for some genes.

d. Comparison of sequencing libraries prepared from the same sample either by nested PCR (as shown in panel a), or by conventional adapter ligation. Barcode counts are expressed as log_{10} of relative abundance. The dynamic range of the method spans five orders of magnitude, but correlation between methods was highest during the upper two decades. The latter therefore defines the range in which we consider barseq counts reliable. The data shown are representative of six samples analysed in this way.

Figure S2. Additional data illustrating the output of barseq screens. Related to Fig. 3.

a. Relative abundance of barcodes in the input vector pools of a typical experiment (left bar chart) is compared to barcodes counted after transfecting the same vector pool and applying drug selection for 7 days. Error bars (right chart only) give standard deviations from three transfections with the same vector pool. Read counts were normalized to the total number of relevant barcode counts for a given sample. Raw data for this experiment are shown in the Supplemental Spreadsheet.

b. Fitness of four protein kinase mutants that were detected only in a second pass barseq experiment from which fast growing mutants except the reference genes had been excluded. Error bars show standard deviations from four independent transfections. Asterisks indicate significant differences determined by a two sided T-test corrected for multiple testing ($p < 0.01$**; $p < 0.05$*).

Figure S3. Genotype confirmation for *gsk3* and *cdpk1* knock out clones. Related to Figs. 5 and 6.

a. Southern blot of restricted gDNA, PCR genotyping and hybridization of separated chromosomes showing disruptions of *gsk3*.

b. Genotyping by PCR and Southern blot of a *cdpk1* deletion mutant after removal of the resistance cassette under negative selection.

Figure S4. Genotype confirmation for PBANKA_082960 and *tkl3* knock out clones. Related to Fig. 5.

a. PCR genotyping strategy and PCR products analysed by agarose gel electrophoresis.

b. Southern hybridization of separated chromosomes before cloning showing integration of a targeting vector for PBANKA_082960 and *tkl3* into the target chromosomes 8 and 13, respectively.

Figure S5. Additional genotype confirmation of *rio* kinase mutants. Related to Fig. 5.

a. Genotyping by PCR of *rio1* and *rio2* mutants after population enrichment by drug selection and two consecutive cloning attempts..

b. Southern hybridization showing integration of the targeting vector for *rio1* and *rio2* into the target chromosomes 14 and 5, respectively.

Supplemental Protocol 1 – Transfection of *P. berghei* parasites

Day 1:
Inject a naïve mouse intraperitoneally with 10^6 *P. berghei* parasites and monitor parasitaemia on Giemsa stained thin blood smears.

Day 3 (in the evening):

Infect a young (200-250 g) Wistar rat by intraperitoneal injection of mouse blood containing 10^6 infected red blood cells diluted to a final volume of 500 µL in phosphate buffered saline (PBS).

Usually day 8 (before 12 pm):

1. When parasitaemia has reached 1-3 %, bleed the rat under isofluorane anaesthesia using a 10 mL syringe loaded with 100 µL of heparin (300 U mL^{-1}) with a 20 G needle.
2. Set up a schizont culture by adding 7 mL of infected blood to 200 mL of schizont culture medium in a sterile 500 mL round bottom culture flask. Gas the culture with malaria gas (5 % CO_2, 5 % O_2, and 90 % N_2) for one minute before closing it tightly. Incubate the culture at 36.5 °C with gentle rotary shaking (just enough to prevent blood from settling).

Schizont culture medium (200 mL): To 144 mL RPMI1640 containing L-glutamine and 25 mM HEPES, without $NaHCO_3$ (Sigma-Aldrich) add 4 mL of 0.5 M $NaHCO_3$ (filter sterilized), 2 mL penicillin/streptomycin (10,000 U mL^{-1}) and 50 mL fetal calf serum (Gibco).

Day 9:

1. At 10 am check schizont maturation on a Giemsa stained thin blood smear. Once schizonts contain individualized merozoites proceed to purification.
2. In each of four 15 mL Falcon tubes prepare a 55 % Nycodenz/PBS cushions (v/v) by mixing 2.75 mL Nycodenz stock-solution (Janse et al., 2006) with 2.25 mL PBS.
3. Pellet the blood containing the schizonts in 50 mL Falcon tubes for 14 min at 300 g with slow acceleration/deceleration. It is important that the centrifuge is at room temperature at all times.
4. Keep 30 mL of supernatant to wash/dilute purified schizonts later on. Remove the supernatant apart from 3-4 mL at the bottom of the tube which will be used to resuspend the cells.
5. Using a plastic Pasteur pipette, gently layer the cells on top of the Nycodenz cushion prepared in step 3.
6. Centrifuge 20 min at 300 g in a swing out rotor again at room temperature (slow acceleration and break, so as not to disturb the gradient).
7. Collect the brown schizont layer at the interface from all gradients into a 15 mL Falcon tube (1 mL from each gradient) and fill it up to the top with the supernatant kept in step 5.

8. Pellet the schizonts by centrifugation (450 g, 3 min, medium acceleration/ deceleration) and discard supernatant.
9. To use schizonts from one rat for 3 replicate transfections of a barcode sequencing experiment add 54 µL (18 µL/transfection) of P3 Primary Cell 4D-Nucleofector (Lonza) to the pelleted schizonts and resuspend them gently.
10. Mix the schizont suspension with 5 µL of digested DNA and transfer 25 µL of the mix into each of three individual wells of a 16-well Nucleocuvette Strip and pulse using programme FI-115 of the 4D-Nucleofector device.
11. Aspirate schizonts using 1 mL insulin syringe, previously loaded with 100 µL of pre-warmed incomplete RPMI.
12. Inject immediately intra-venously into BALB/c mice.

Day 10:
Early in the morning take thin blood smears to calculate transfection efficiency before starting drug selection with drinking water containing pyrimethamine (0.07 mg/mL).

Supplemental Protocol 2 – Genomic DNA isolation (phenol-chloroform method) for barcode sequencing

1. Lyse the red blood cells by adding 1 mL of ammonium chloride solution (0.15 M NH_4Cl, 0.01 M $KHCO_3$, 0.001 M EDTA) to the collected blood. Incubate for 2 min on ice.
2. Pellet the parasites (3000 g) and resuspend them in 500 µL of TNE buffer (10 mM Tris pH 8.0, 5 mM EDTA pH 8.0, 100 mM NaCl).
Add 2 µL of RNase A (20 mg/mL), 55 µL of 10 % SDS and incubate for 10 min at 37 ℃.
3. Add 10 µL of proteinase K (20 mg/mL), vortex and incubate at 37 ℃ for 30 min.
Add 500 µL of buffered phenol:chloroform:isoamylalcohol (25:24:1), invert several times and centrifuge for 5 min at 10,000 g.
4. Transfer the aqueous upper phase into a new tube. Add 500 µL of chloroform:isoamylalcohol (24:1), invert samples several times and centrifuge for 5 min at 10,000 g.
5. Transfer the new aqueous upper phase to a new tube, and precipitate DNA overnight at -20 ℃ with 500 µL of isopropanol.

OLIGONUCLEOTIDE SEQUENCES

Primer name	Sequence	Target/Comment
arg00080	GACTTCTGTAGCCATGATAGC	hdhfr::yfcu
arg00081	CGCCACACTACATGGTGAG	hdhfr::yfcu
arg00084	AAAGAATTCTGATGGTTTACAATCACC	RNApol II
arg00085	AAAGCGGCCGCTTTCTTCCTGCATCTCCTC	RNApol II
arg00160	AGCGAGTTCCCGTGCACTCA	
arg00331	TTGAACATTTGCGCATATATTGG	
arg00332	GTGCCAAATTATTATGGTATACC	
arg00333	TGATCTAGAGATAAAAGAGGAC	
arg00334	CTTCATATTTTACCTGTATGCC	
arg00421	GTGCCAAATTATTATGGTATAC	
arg00422	CCAGAATAATTTTGTTAGAATATAG	
arg00423	GTACAGTTTGTGGTATATATTCC	
arg00424	GGTGATAATAATGCATGCCAAG	
arg00371	CCGGTATTTATCATCCAAGAG	
arg00372	CATTTGTTCATGCAATCATTCG	
arg00373	CCCACTACCTAATTTCCGAAC	
arg00374	GCATCATTGACACGAACTCG	
arg00375	GTGAAGAGAGGCTAAGGAGG	
arg00425	GGTTCTATCTGTTTCATGTAC	
arg00426	CCTCAGAAAATGAATGGCAG	
arg00427	GGTGTAATCAAAGTAAAAGTGC	
arg00428	CTCCACCTTCATAAAATTCGG	
arg00396	GGCATGCCGTATTTTCCATG	
arg00397	ATGCATATTCTTTTGTTGCAGC	
arg00398	CACCCATCCAAACATATAAAAG	
arg00399	CTAGTAATAGTCAGTCTGGGG	
arg00400	TCCAAAGCGTTATGCCAAGTGT	
arg00401	GCAACTCGTTATATATTTCCG	
arg00402	CTTTTCCAGAACTAACTACTCC	
arg00403	CTTTTCTTGTAACACCCTCAG	
arg00404	TGGAACTACAAAATAGATTTCG	
arg00405	GCTCAAGCAACAGCAGGAC	
arg00496	CGTTTTTCTTACTTATATATTTATACCAATTGATTGT	PFGE probe
arg00497	TCGAAATTGAAGGAAAAAACATCATTTG	PFGE probe
arg00216	CGGGGCCCTTATGCATAATC	
arg00218	CTTTGGTGACAGATACTACTG	
arg00406	ATCCAAATATGGTATTTTGAGC	
arg00407	CTGGAGATTCGTTTTGTTCAC	
arg00408	TTGTGTTTGCCGCATGTTGC	
arg00409	GATATTCCTAAAGATCTATCTG	
arg00410	ACGACAATGTGCATGCCTCA	
arg00416	CGTACTTGAATAGCTGTCTAC	
arg00417	CCCCAATATAATGAATATTCTG	
arg00418	GAGCATTCCGCAAAGTATGTC	
arg00419	GGGAACACATCCTTTTAGTTC	
arg00420	AGCCATTACCCGTTGTTTCG	
arg00215	TCATTCTTCGAAAACGATCT	zeocin
arg00083	CCGCCTACTGCGACTATAGA	zeocin
arg00218	CTTTGGTGACAGATACTACTG	
arg00057	GAAACACAATGTTGAAATTC	*map2*
arg00102	GTGTAGAAGTAAATTCATACCC	*srpk2*
arg00370	GGTTATGAGAAGTTAAACTACG	*cdpk4*

SUPPLEMENTAL REFERENCE

Janse, C.J., Ramesar, J., and Waters, A.P. (2006). High-efficiency transfection and drug selection of genetically transformed blood stages of the rodent malaria parasite Plasmodium berghei. Nature protocols *1*, 346-356.
