# Supplementary material for: A Genome-Scale Vector Resource Enables High-Throughput Reverse Genetic Screening in a Malaria Parasite
Source: Cell Host Microbe. 2015 Mar 11;17(3):404–13. doi: 10.1016/j.chom.2015.01.014 (PMC4362957; doi:10.1016/j.chom.2015.01.014)
Supplement: Document S2. Article plus Supplemental Information [file mmc3.pdf]

Resource

Cell Host & Microbe

A Genome-Scale Vector Resource Enables High-Throughput Reverse Genetic Screening in a Malaria Parasite

Graphical Abstract

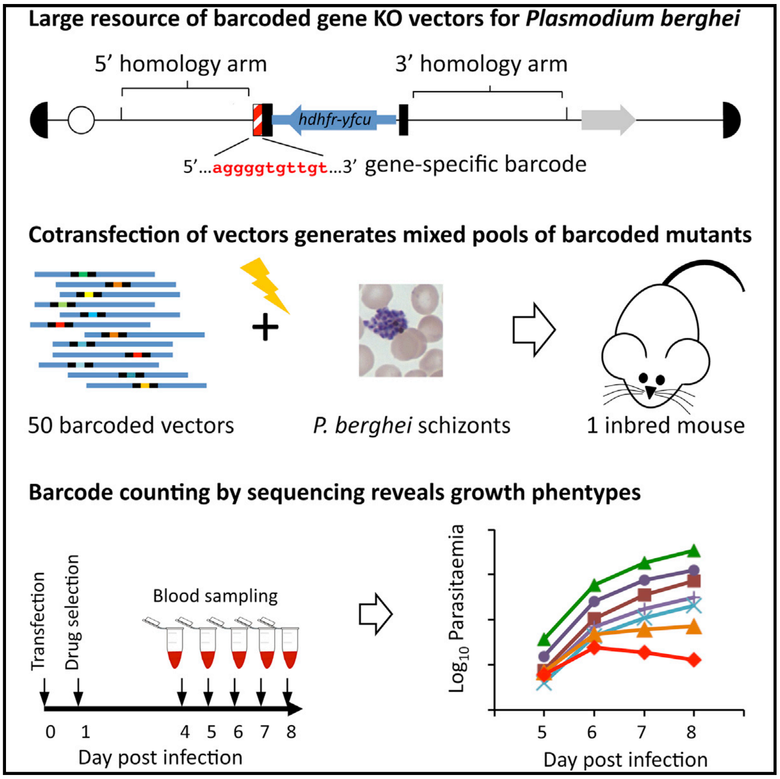

Highlights

- Barcoded genetic modification vectors used to create a resource for *P. berghei*
- Parallel phenotyping of dozens of mutants can be performed using barcode sequencing
- A validation barseq screen of the kinome reveals false-positive and -negative rates
- Targetable kinase genes identified and verified with cloned mutants

Authors

Ana Rita Gomes, Ellen Bushell, ..., Julian C. Rayner, Oliver Billker

Correspondence

julian.rayner@sanger.ac.uk (J.C.R.),
oliver.billker@sanger.ac.uk (O.B.)

In Brief

Gomes et al. present a large-scale resource of barcoded genetic modification vectors with high integration efficiency for *Plasmodium berghei*. The resource enables reverse genetic screening by allowing pools of barcoded mutants to be created in a single mouse and phenotyped in parallel by counting barcodes through next generation sequencing.

Gomes et al., 2015, Cell Host & Microbe *17*, 404–413
March 11, 2015 ©2015 The Authors
http://dx.doi.org/10.1016/j.chom.2015.01.014

Cell Host & Microbe
Resource

A Genome-Scale Vector Resource Enables High-Throughput Reverse Genetic Screening in a Malaria Parasite

Ana Rita Gomes,[1,2] Ellen Bushell,[1,2] Frank Schwach,[1] Gareth Girling,[1] Burcu Anar,[1] Michael A. Quail,[1] Colin Herd,[1] Claudia Pfander,[1] Katarzyna Modrzynska,[1] Julian C. Rayner,[1,*] and Oliver Billker[1,*]
[1]Wellcome Trust Sanger Institute, Hinxton Cambridge CB10 1SA, UK
[2]Co-first author
*Correspondence: julian.rayner@sanger.ac.uk (J.C.R.), oliver.billker@sanger.ac.uk (O.B.)
http://dx.doi.org/10.1016/j.chom.2015.01.014
This is an open access article under the CC BY license (http://creativecommons.org/licenses/by/4.0/).

SUMMARY

The genome-wide identification of gene functions in malaria parasites is hampered by a lack of reverse genetic screening methods. We present a large-scale resource of barcoded vectors with long homology arms for effective modification of the *Plasmodium berghei* genome. Cotransfecting dozens of vectors into the haploid blood stages creates complex pools of barcoded mutants, whose competitive fitness can be measured during infection of a single mouse using barcode sequencing (barseq). To validate the utility of this resource, we rescreen the *P. berghei* kinome, using published kinome screens for comparison. We find that several protein kinases function redundantly in asexual blood stages and confirm the targetability of kinases *cdpk1*, *gsk3*, *tkl3*, and PBANKA_082960 by genotyping cloned mutants. Thus, parallel phenotyping of barcoded mutants unlocks the power of reverse genetic screening for a malaria parasite and will enable the systematic identification of genes essential for in vivo parasite growth and transmission.

INTRODUCTION

The rate at which the genomes of intracellular malaria parasites can be modified has remained largely unchanged since methods for gene targeting by homologous recombination were developed in *Plasmodium* (Crabb and Cowman, 1996; van Dijk et al., 1996; Wu et al., 1995). Some notable advances have recently improved transfection efficiency in *P. falciparum* through the application of zinc finger nucleases (Straimer et al., 2012) and CRISPR-Cas9 (Ghorbal et al., 2014; Wagner et al., 2014). However, no currently available method is efficient enough to enable reverse genetic screens, and transposon mutagenesis in *P. falciparum* is at present well short of genome saturation (Balu and Adams, 2006). As a result, more than half of the protein coding genes in *Plasmodium* genomes still lack functional annotation.

Genome-wide collections of mutants or genetic modification vectors have greatly facilitated the discovery of gene functions in model organisms (Ni et al., 2011; Sarov et al., 2006; Skarnes et al., 2011; Winzeler et al., 1999). In malaria parasites, in contrast, efforts to scale up reverse genetics have suffered from a combination of low rates of homologous recombination and a high content of adenine and thymine (A+T) nucleotides that renders *Plasmodium* DNA difficult to engineer in *E. coli*. A malaria parasite of rodents, *P. berghei*, offers the most robust system for genetic manipulation with relatively high transfection efficiency (Janse et al., 2006a). In this species homologous integration can be boosted further by transfecting linear vectors with long (4–8 kb) homology arms (Pfander et al., 2011). Despite its high A+T content (>77%), *P. berghei* genomic DNA (gDNA) can be propagated efficiently in *E. coli* as large genomic inserts of up to 20 kb using a low-copy bacteriophage N15-derived linear plasmid with covalently closed hairpin telomeres (Godiska et al., 2010). In contrast to high-copy circular plasmids, an N15-based arrayed gDNA library achieved nearly complete genome coverage with sufficient insert size to represent the majority of *P. berghei* genes in their entirety. Clones from this library can be converted into gene targeting and tagging vectors in 96 parallel liquid cultures using robust protocols (Pfander et al., 2011), which exploit highly efficient homologous recombination mediated by the Red/ET recombinase system of *lambda* phage in *E. coli* (Zhang et al., 2000).

To accelerate the functional analysis of all *P. berghei* genes we here present a genome-scale community resource of long-homology genetic modification vectors that are individually quality controlled by sequencing and carry gene-specific molecular barcodes. The availability of more than 2,000 genome modification vectors raises the possibility of generating a large library of cloned and genotyped *P. berghei* mutants of the type that has enabled global genetic screens in yeast (Giaever et al., 2002; Winzeler et al., 1999). However, in *P. berghei* the lack of continuous in vitro culture of blood stages would limit the utility of such a clone collection. Signature-tagged mutagenesis, whereby thousands of mutants are simultaneously screened in a pooled approach (Hensel et al., 1995; Langridge et al., 2009; Mazurkiewicz et al., 2006), therefore offers a more attractive strategy for scaling up reverse genetics in *P. berghei*.

We have used the modification vector resource to enable such systematic screens for a *Plasmodium* parasite. We demonstrate that cotransfecting multiple gene knockout vectors in the same

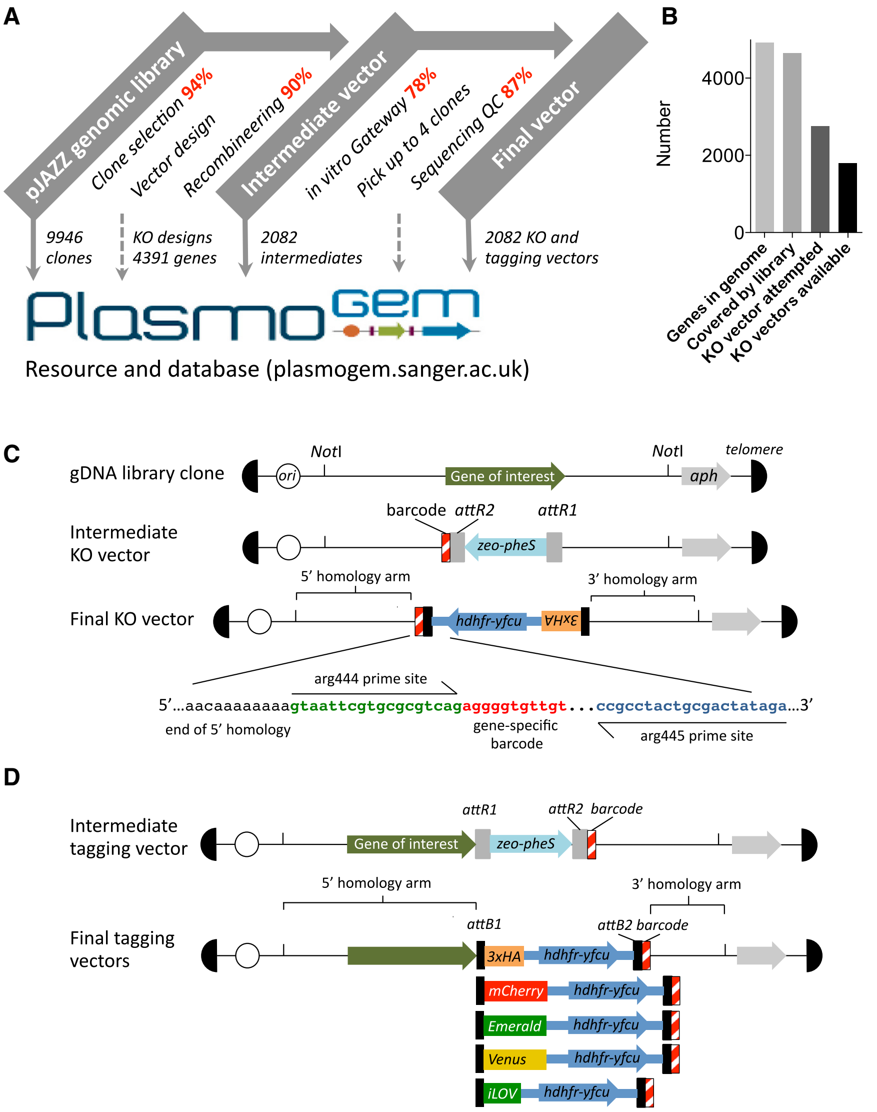

Figure 1. *Plasmo*GEM: A Genome Scale Free Resource of Genetic Modification Vectors for *P. berghei* Reverse Genetics

(A) A diagram of the modular vector production process showing the efficiency at each step (red), as well as resources (gray boxes) and data (dashed lines) submitted to the database.

(B) Genome coverage achieved to date.

(C) Schematic showing knockout vector designs and locations of the gene-specific molecular barcode included in each vector.

(D) Default C-terminal epitope-tagging vector and a panel of alternative fusion tags.

electroporation reproducibly generates complex pools of barcoded *P. berghei* mutants, and develop a barcode sequencing (barseq) approach (Smith et al., 2009) to phenotype the growth rates of all mutants within the pool over the course of an infection. To validate the approach, we compared a barseq knockout screen of protein kinases with the conventional kinome screen by Tewari et al. (2010). This comparison showed high reproducibility with previous data, but the sensitivity and robustness of the barseq approach also identified additional targetable genes. Our analysis demonstrates the power of barseq screening to robustly provide growth-rate phenotypes for dozens of mutants in single mice, and opens up the possibility for large-scale reverse genetic screens for multiple areas of *Plasmodium* biology.

RESULTS

A Resource of Efficient Gene Targeting Vectors for *P. berghei*

To generate a genome-scale resource of gene knockout vectors, we used a modular pipeline for recombinase mediated engineering in *E. coli* (Pfander et al., 2011). The parasite gene of interest was first replaced in appropriately chosen gDNA clones with a marker for positive and negative selection in *E. coli* using Red/ET recombinase-mediated engineering. The bacterial markers were then exchanged under negative selection for a drug resistance cassette for *P. berghei* in a single in vitro Gateway recombinase reaction. When applied to the 2,781 *P. berghei* genes that have any level of functional annotation (57% of the genome), a first pass of the production pipeline yielded gene deletion vectors for 1,868 different protein coding genes of the core nuclear genome (Figures 1A–1C). These vectors form the foundation of the Plasmo*dium* gene*tic* *m*odification resource, *Plasmo*GEM (Figure 1A), which can be viewed and requested through a searchable database at http://plasmogem.sanger.ac.uk (Schwach et al., 2015).

The vector production strategy can also be adapted to generate other modifications, including C-terminal tagging vectors, of which there are currently 278 in the resource. Tagging vectors are constructed with a C-terminal triple HA epitope tag by default. For each final vector we also make uncloned intermediate constructs available, which users can convert into targeting vectors with different functionality. A panel of Gateway entry clones are available which contain fusion tags for epitopes and different fluorescent proteins (Figure 1D). Each *Plasmo*GEM vector carries a gene-specific molecular barcode of 10–11 nucleotides that uniquely identifies genetically modified parasites derived from it (Figures 1C and 1D), and all final *Plasmo*GEM vectors contain the *hdhfr-yfcu* marker that enables positive and negative selection in vivo (Braks et al., 2006).

Manual construct design became rate limiting for vector production, and we therefore created a suite of computational tools to select gDNA library clones; to choose 50 bp homology regions for recombination in *E. coli*; to design oligonucleotides, generate sequence maps, assign barcodes, and track vectors through the production process; and to automate quality control (Schwach et al., 2015). The long homology arms of *Plasmo*GEM vectors

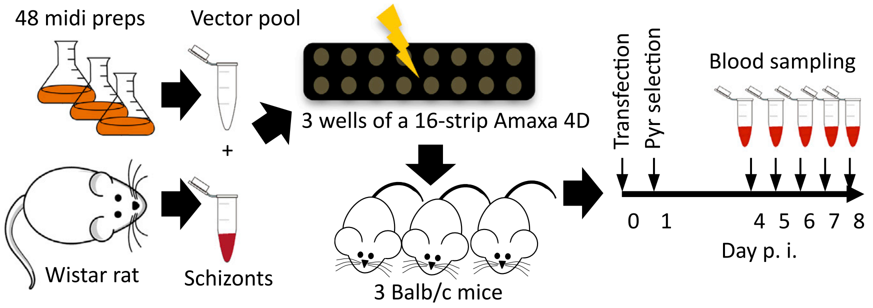

Figure 2. Schematic Representation of a Typical Barcode Sequencing Experiment
For each experiment three inbred mice are infected from a separate transfection of the same vector pool.

(average sum of both arms 7.4 ± 2.9 kb SD) enhance recombination frequency in *P. berghei* compared to conventional designs (Pfander et al., 2011), but they also pose a risk that unintended mutations get inadvertently incorporated into the parasite genome. Homology regions and barcode modules of all targeting vectors are therefore confirmed by next-generation sequencing and pass stringent QC standards before they are used (for details, see https://plasmogem.sanger.ac.uk).

Parallel Phenotyping of Dozens of *Plasmo*GEM Mutants by Barcode Counting

To examine whether the improved integration efficiency of *Plasmo*GEM vectors would permit the reproducible generation and phenotyping of mixed pools of barcoded mutants, we selected from the resource 46 vectors targeting protein kinases that had previously been part of a systematic deletion analysis using conventional vectors (Tewari et al., 2010). Four sexual and mosquito-expressed genes known to be dispensable for asexual growth were also included to serve as reference genes to which growth rates of all other mutants could be compared. This reference set was comprised of knockout vectors for the major surface proteins of the ookinetes, P25 and P28 (Tomas et al., 2001); a secreted ookinete adhesive protein, SOAP (Dessens et al., 2003); and a C-terminal tagging vector for the redundant *p230p* gene (van Dijk et al., 2010). In addition, three further control vectors were included to assess our ability to detect reduced growth rates in blood stages. One of these targeted plasmepsin IV, an aspartic protease involved in hemoglobin degradation whose deletion in *P. berghei* results in attenuated growth (Spaccapelo et al., 2010). We predicted parasite growth would be reduced by a deletion vector for PBANKA_110420, which encodes the E1β subunit of the mitochondrial branched chain α-ketoacid dehydrogenase (BCKDH), given that deleting the E1α subunit of the same complex has a clear growth phenotype (Oppenheim et al., 2014). A third attenuating knockout vector targeted PBANKA_140160, a putative methyl transferase of unknown function, which emerged as a slow-growing mutant from a preliminary screen of metabolic enzymes (data not shown).

Schizonts cotransfected with a cocktail of 48 vectors and injected into mice (Figure 2) gave rise to drug resistant parasites 4 days later. This was indicative of an overall transfection efficiency of $\sim 10^{-4}$ and suggested that roughly 2,500 independent recombination events occurred in a transfection, enough to generate a complex mixture of mutants. Blood samples were subsequently collected exactly every 24 hr from day 4, gDNA was extracted, and vector-specific barcodes were amplified by a polymerase chain reaction (PCR) with a generic primer pair (see Figure S1A available online) and counted on a benchtop next-generation sequencer (Figure 2).

In each of two replicate experiments, the same 22 barcodes from the vector pool were robustly detected and yielded nearly identical growth curves (Figure 3A). Southern hybridization of separated chromosomes showed evidence for vector integration events throughout the genome (Figure S1B). Long-range PCR products confirmed integration events for 17 of the 22 replicating barcodes (Figure S1C).

To assess the accuracy of barcode counting, we analyzed the same infected blood samples using two different methods for turning the initial PCR product after barcode amplification into sequencing libraries. The staged PCR strategy shown in Figure S1A and a conventional adaptor ligation protocol performed equally well, producing highly correlated barcode counts (Figure S1D). The PCR strategy proved faster and more economical and was therefore used in subsequent experiments. Figure S2A illustrates for a typical experiment the relative abundances of barcodes in a pool of transfected vectors and in infected mice 7 days after transfection, as determined by the PCR method.

To analyze growth curves derived from barcode counting we considered two parameters: (1) the relative abundance of each barcode within the pool, and (2) the relative fitness of each mutant, i.e., the rate at which its abundance changed each day. As expected, the four barcodes corresponding to control genes redundant for asexual development replicated rapidly. These were taken to represent wild-type growth (fitness $w = 1$). Relative abundance and growth rates were both highly reproducible for each barcode between technical and biological replicates (Figures 3B and 3C). We propose that the shape of a growth curve provides a quantitative measure for the fitness of a mutant. In contrast, the relative abundance of a mutant within a pool we consider less informative, since it may be influenced by a number of additional factors, such as the abundance of a vector in the transfection cocktail, the length of its homology arms, or any local variation in recombination rates. Plotting day 7 fitness from a ranked list (Figure 4A) showed that while the relative abundance of vectors in the transfection cocktail varied by up to one order of magnitude (Figure 4A, right axis), this was not a predictor of fitness of the resulting mutants. Fitness is therefore driven by growth rate, not by the amount of a given vector in the starting pool. The attenuating control vectors were associated with a measurable reduction in parasite fitness to between 0.60 and 0.73, as expected. The majority of protein kinase mutants either had wild-type fitness or were not detected ($w = 0$). While all reference barcodes robustly replicated close to the average fitness of 1.0 (Figure 4B), the attenuating vectors and some kinase mutants showed statistically significant reductions in fitness that were consistently measured across different days of the infection (Figure 4C). Taken together, these data strongly

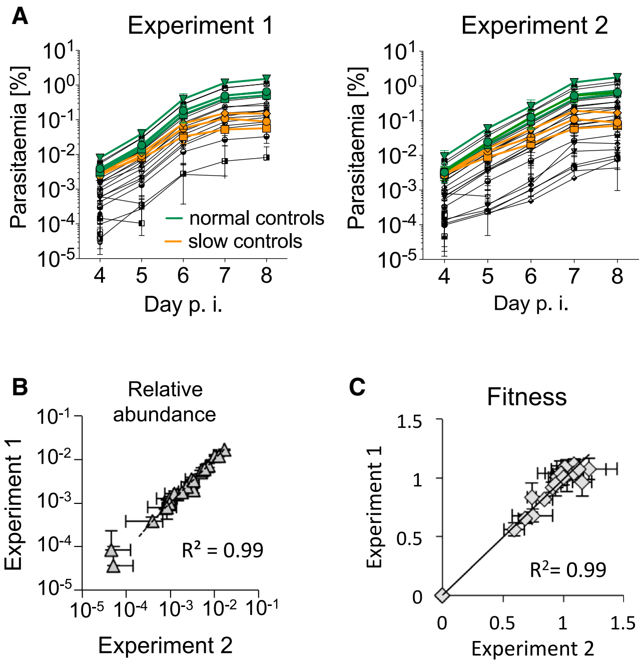

Figure 3. Reproducibility of Independent Barcode Counting Experiment with Respect to the Abundance and Relative Replication Rates of All Barcodes

(A) Each experiment involved three replicate transfections of a different schizont culture performed on a different day and using independently prepared vector pools. Error bars show standard errors (n = 3 per experiment). Green lines, four sexual stage genes (*p25*, *p28*, *p230p* 3xHA tag, and *soap*). Orange lines, three attenuated mutants (plasmepsin IV, PBANKA_110420, PBANKA_140160). Twenty-two mutants are shown in total. See Figure S1 for genotyping data.

(B) Linear regression analysis of mean abundance values for the two experiments shown in (B). All barcodes present until day 8 posttransfection were included. Error bars show standard errors of the mean (n = 3).

(C) Regression analysis of average mean fitness for each barcode between days 5–8 posttransfection for the two biological replicates in (B). Fitness is calculated from the replication rate of the gene-specific barcode relative to the mean of the four sexual stage reference genes. Error bars show standard errors (n = 3). See Table S1 for fitness measurements for individual vectors, and Table S4, illustrating data analysis.

suggest that barcode counting can be used to phenotype large numbers of mutants in parallel.

We hypothesized that in pools some slow-growing mutants may be outcompeted by faster-growing mutants before their barcodes can be detected. To eliminate this potential source of error, we performed a second-pass screening strategy by pooling only the slow or nonreplicating vectors from the previous experiments and transfecting them together with the reference set. This allowed us to measure the fitness of four additional mutants, one whose vector had a very low integration efficiency (PBANKA_040940), and three that were characterized by low growth rates during part or all of the infection (Figure S2B). These data suggest that the size and complexity of a vector pool can be increased only at the expense of losing more slow growing mutants. However, this loss can be compensated in a second-pass screen of vectors that are not detected in the first experiment.

Barcode Counting Reveals Protein Kinase Mutants

To assess the accuracy of barseq screening, we compared the combined results from five barseq experiments with the data from our previous knockout screen, which used conventional vectors in a gene-by-gene approach with careful genotyping of cloned mutants (Tewari et al., 2010). For the majority of genes (76.1%) covered by both studies, the replication of barcodes in the mixed pool of mutants was predicted by previous data for their targetability (Table S1; Figure 5A). Two genes shown previously to be dispensable in blood stages (Tewari et al., 2010) could not be targeted in barseq screens. Failure of these vectors to integrate was not due to low amounts of vector DNA in the transfection cocktail and in the case of *pk7* was reproduced when vectors were transfected individually. Absence of integration may be the result of low recombinogenicity at the target locus, or individual vector designs may interfere with the expression of essential neighboring genes. Although the fraction of technical failures was small, these observations reaffirm the need to confirm genetic essentiality by conditional methods, just as in conventional gene-by-gene studies.

Although some false negatives can be tolerated, genetic screens rely critically on the rate of false positives being very low. In barcode sequencing experiments false positives may result from episomal replication of vector DNA or from nonhomologous integration of targeting vectors. Although the former is commonly observed in *P. berghei* after transfecting vectors that originate from circular plasmids, we have not yet encountered spontaneous circularization of *Plasmo*GEM vectors, which rely on the expression of a phage telomerase to replicate in a linear form in *E. coli* (data not shown). The strongest candidates for false positives in our pilot screen were ten protein kinase genes for which barcode counting revealed evidence for their disruption by *Plasmo*GEM vectors, while previous studies had failed to delete the same genes using conventional vectors (Sebastian et al., 2012; Tewari et al., 2010). We selected six of these genes for validation by targeting them individually.

Two genes, *cdpk1* and *gsk3*, have been considered potential drug targets in *P. falciparum* (Droucheau et al., 2004; Kato et al., 2008), but are shown here to be targetable in *P. berghei* (Figures S3A and S3B). In the case of *cdpk1* our data confirm another recent study (Jebiwott et al., 2013), which also showed that the unconditional disruption of *cdpk1* has no impact on blood stage growth but reproduces the ookinete phenotype we recently described for a stage-specific mutant in the same gene (Sebastian et al., 2012). Generating and genotyping individual knockout clones for *gsk3*, *tkl3*, and PBANKA_082960, a putative protein kinase of unknown function, confirmed the barseq phenotype and established that these genes are indeed dispensable for blood stage growth (Figure S4).

We next turned to the *rio* group of protein kinases. *rio1* and *rio2* encode ancient atypical protein kinases that are highly conserved in most archaea and all eukaryotes, where they perform essential functions in ribosome biogenesis (LaRonde, 2014). In pooled transfections, barcodes for both genes were close to the detection threshold, and only the *rio1* barcode was detected consistently at different time points. We therefore classed *rio1* as targetable and *rio2* as likely essential, although the latter produced a small number of replicating barcodes in some transfections. Targeted disruption of each *rio* gene individually yielded

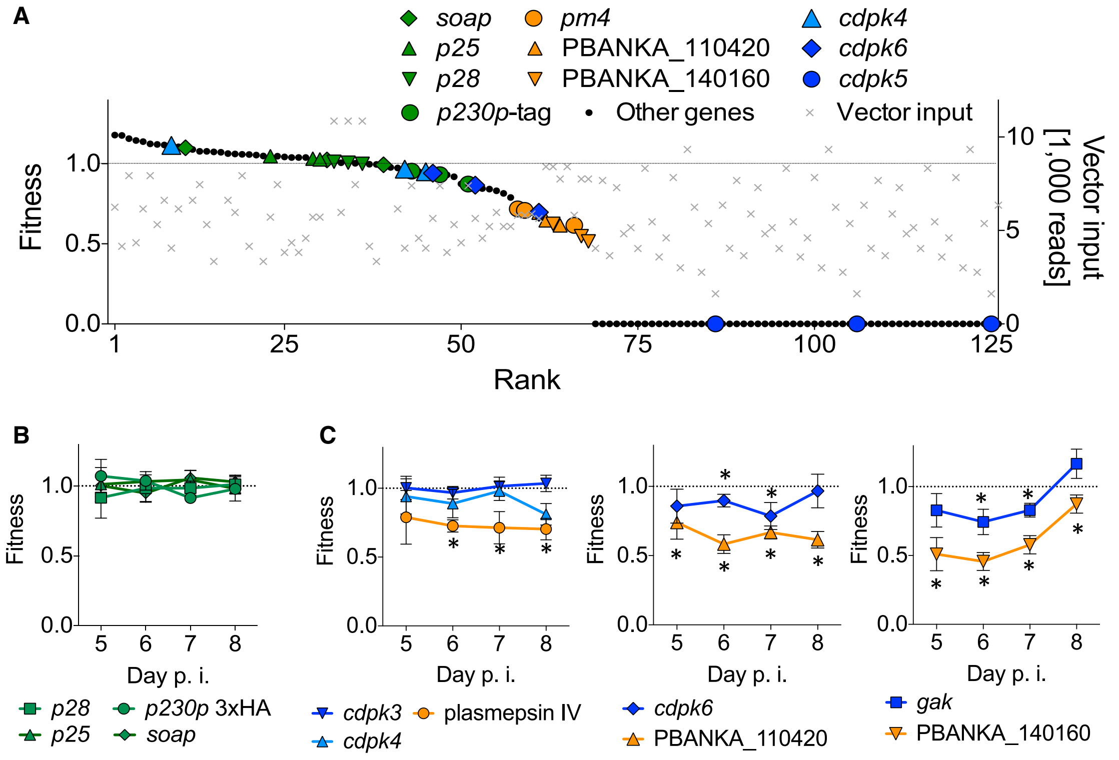

Figure 4. Fitness Measurements Obtained with *Plasmo*GEM Vectors Targeting Protein Kinases

(A) Distribution plot generated from a ranked list of day 7 fitness values measured in triplicate for each of 42 genes in experiment 1 (left axis). The relative abundance of a targeting vector in the electroporation cuvette at the moment of transfection (gray crosses, right axis) did not predict whether a mutant could be obtained. See Figure S2 for relative abundance data of a representative replicate experiment.

(B) Fitness of reference mutants averages 1 by definition. Error bars show standard errors (n = 6).

(C) Fitness of selected mutants. Error bars as in (B). Asterisk, different from reference mutants as determined by a two-sided t test corrected for multiple testing ($p < 0.01$; n = 6).

resistant parasites after a delayed prepatency period of 10–14 days. Resistant parasites grew very slowly under drug pressure and resisted dilution cloning.

Whole-genome sequencing of a putative *rio1*$^{-}$ population confirmed the nearly complete absence of coverage of the target gene, consistent with the uncloned population being strongly enriched in parasites carrying a correctly integrated *rio1* deletion vector (Figure 5B). Targeting of *rio2*, in contrast, selected for a 29.7 kb duplication on chromosome 5 that included the *rio2* locus, together with three other genes (Figure 5B). Read coverage suggested these parasites carried one disrupted and one intact copy of the target gene, which was consistent with PCR evidence showing the presence of both a disrupted and a wild-type *rio2* locus in the mutant population (Figure S5A) and Southern hybridization of separated chromosomes showing integration of the *rio2* targeting vector into chromosome 5, as expected (Figure S5B). These data fit a model in which a pre-existing partial genome duplication can predispose a small proportion of the parasite population to survive integration of a deletion vector for an essential gene. Partial genome duplications of a similar size exist transiently in *P. falciparum*, where they serve as starting points for the evolution of drug resistance (Guler et al., 2013).

Of six kinase genes selected for follow-up as potential false positives, only the least robustly detectable one proved to be a false hit, while the other five revealed targetable genes missed by an earlier screen (Figure 5C). From these data it is highly plausible that replicating barcodes represent correctly integrated vectors in the vast majority of cases. False positives may be due to pre-existing short genome duplications, but such events are rare. In yeast rare events are filtered effectively by disregarding data from the 10% of least abundant mutants in barseq screens (Robinson et al., 2014). Such approaches can be applied to future *P. berghei* screens to reduce the false-positive rate even further.

Finally we asked whether the interpretation of barcode counting data from parallel transfection experiments could be confounded by multiple vectors integrating into the same parasite genome. First we transfected pools of barcoded vectors into three parasite clones, each of which already carried a different barcode from the insertion of a targeting vector and subsequent recycling of the selection marker. We reasoned that if each mutant integrated one new vector, the pre-existing barcode should account for exactly half of all barcode counts after transfection and drug selection. The data were entirely consistent with this model (Figure 6A), suggesting that if multiple integration events existed, they would be too rare to be isolated by limiting dilution cloning. To detect rare double integration events, we chose three genes that are readily disrupted using *Plasmo*GEM

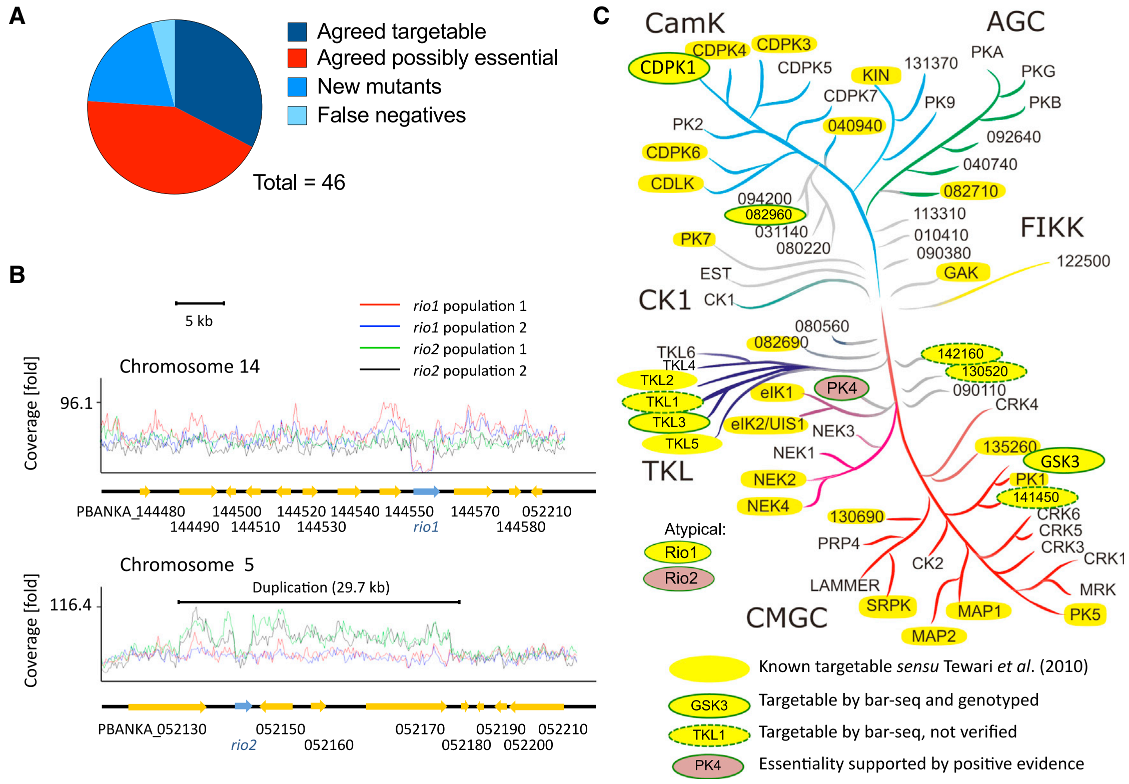

Figure 5. Barcode Sequencing Is Validated by a Comparison with Published Data and Genotyping of Mutants

(A) Barseq screen of 46 *Plasmo*GEM vectors targeting protein kinases compared to the conventional kinome screen by Tewari et al. (2010).

(B) Read coverage from whole-genome sequencing of highly enriched mutant populations showing deletion of *rio1* in a haploid genome (upper panel), and insertion of a *rio2* knockout vector associated with stabilization of a 29.7 kb duplication including *rio2*. See Figure S5 for additional genotyping data.

(C) Updated tree showing targetable and essential *P. berghei* protein kinases. Targetability of *cdpk1* was independently shown by Jebiwott et al. (2013). A role for PK4 in blood stage growth was demonstrated by Zhang et al. (2012). See Figures S3 and S4 for genotype confirmation of cloned mutants for the knockouts.

vectors. For each we transfected the final knockout vector together with a 20-fold excess of the selection marker-free intermediate vectors for the other two genes. We expected to detect replication of the marker-free vectors by PCR, but only if their genomic integration coincided with integration of the selectable construct into the same genome. These experiments failed to generate evidence for multiple integration events into the same genome (Figure 6B). The data indicate that different homologous integration events in *P. berghei* are independent, and suggest that DNA uptake after electroporation is not the factor limiting transfection efficiency.

Taken together these data demonstrate that barcode counting provides a powerful tool to identify targetable *P. berghei* genes and to rapidly and reliably measure the competitive fitness of dozens of mutants during asexual erythrocytic growth in a way that is neither confounded by double integration events nor likely to generate an excessive number of false-positive or false-negative results.

DISCUSSION

The *Plasmo*GEM resource has begun to facilitate conventional gene knockout and tagging experiments in *P. berghei* by providing robust reagents for use in biological studies (see for instance Frénal et al., 2013). While our current emphasis is on generating and distributing the largest possible resource of knockout vectors, the same pipeline can be used to manufacture barcoded constructs that modify the 5′ or 3′ end of a gene. Due to the modular nature of the pipeline, the same intermediates can already be used to generate vectors for fluorescence and epitope tagging, and we anticipate that inducible systems that rely on regulatable promoters, degrons or aptamers (Armstrong and Goldberg, 2007; Goldfless et al., 2014; Pino et al., 2012) can be scaled up using the same approach. Recombinase-mediated engineering has already facilitated the generation of conditional alleles for inducible knockout approaches that rely on Flp recombinase (Suarez et al., 2013), and a similar strategy may be used for the insertion of loxP recognition sites for Cre-mediated recombination, which is becoming an important tool for generating inducible alleles in *Plasmodium* (Collins et al., 2013).

As well as being highly versatile, we demonstrate here that *Plasmo*GEM vectors can be used to perform functional screens. Three lines of evidence support our conclusion that fitness measurements based on barcode counting generally reflect homologous integration events at the target locus: (1) a barseq screen of protein kinases generated data that were in good agreement with the previous conventional knockout study by Tewari et al. (2010); (2) barcodes of essential kinase genes did not replicate

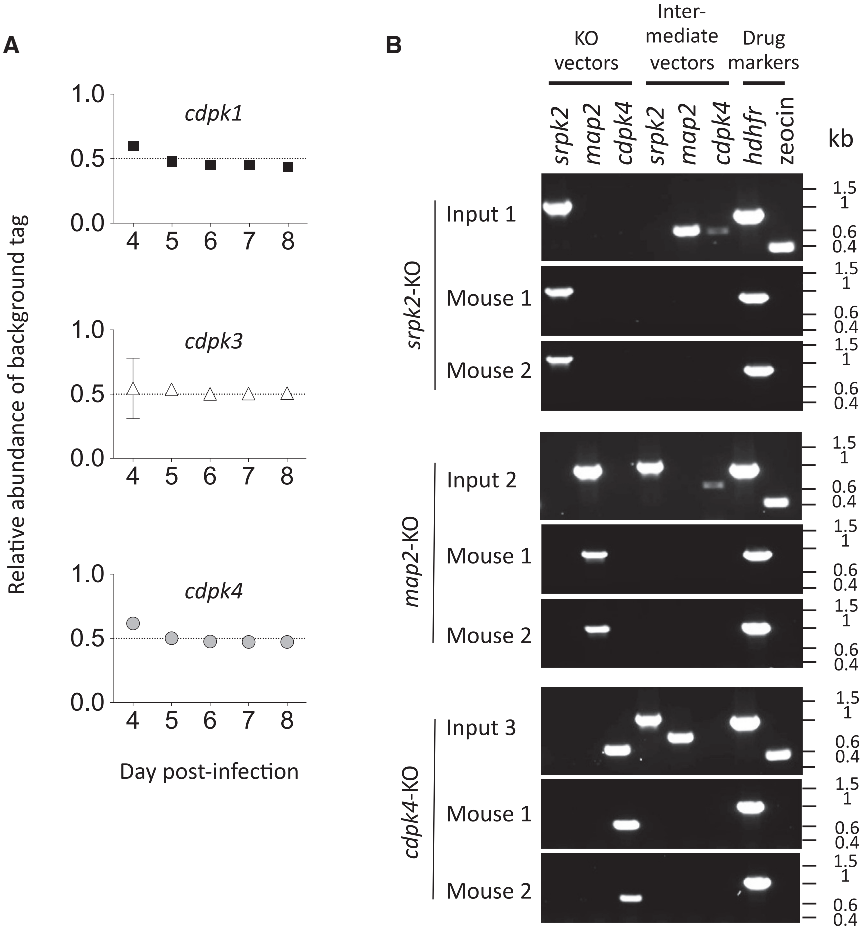

Figure 6. Absence of Evidence for Multiple Integration Events

(A) Vector pools were transfected into marker-free lines with pre-existing barcodes in *cdpk1*, *cdpk3*, or *cdpk4*. New barcodes account for approximately half of the total, as would be expected if each parasite genome carried exactly one new barcode. The slight overrepresentation of background barcodes on day 4 probably comes from parasites that failed to integrate a vector and which were not yet completely eliminated after only 3 days of drug selection. All data points are supported by three experiments, and error bars show standard deviations. See Figure S3 for genotyping of marker-free lines.

(B) PCR genotyping was performed on parasite gDNA from six infected mice, each transfected with one of three final targeting vectors in the presence of a 20-fold excess of intermediate vectors (10 μg total DNA per transfection), which have the same homology arms but a zeocin resistance cassette that cannot be selected in *P. berghei*. Presence of intermediate vectors in the input cocktail but absence in the resistant parasite populations suggests that multiple integration events are rare or absent, since hitchhiking of marker free insertions would otherwise be observed.

in the parasite, showing that nonintegrated vectors are not maintained episomally but are effectively eliminated by day 4 of the infection; (3) unexpected mutants were in genes that we later verified as targetable by transfecting individual vectors and genotyping the resulting mutants. In one instance the inconsistent replication of a barcode raised the unexpected possibility of genetic redundancy of *rio2*, until whole-genome sequencing revealed a 29.7 kb duplication containing a second intact copy of the target gene. Whole-genome sequences of many rodent *Plasmodium* genomes are now available and contain no evidence that genome duplications commonly become fixed (Otto et al., 2014), possibly because of associated fitness costs. False positives of the *rio2* type will therefore always be rare events that can be filtered out, for instance by eliminating the least abundant 10% of mutants from the analysis, as has been recommended for barseq screens of yeast mutants (Robinson et al., 2014).

The use of vector pools to phenotype mixtures of mutants is particularly suited to *P. berghei*, where a library of cloned mutants would be of limited value, because the absence of an in vitro culture system for blood stages means that each cloned mutant has to be generated and propagated in mice. In marked contrast, the human malaria parasite *P. falciparum* has an efficient culture system for blood stages, but low recombination rates require transfection with circular vectors, and our preliminary data suggest that linear vectors of the type used here do not spontaneously integrate into the genome, even when their homology arms are each several kilobases long. The CRISPR-Cas9 system has recently been used successfully to transfect linear DNA in *P. falciparum* (Ghorbal et al., 2014). If this method increases recombination rates significantly it raises the exciting prospect of generating large numbers of barcoded *P. falciparum* mutants that could then be pooled for genetic screens using barseq screening.

The scale of barseq screens in *P. berghei* is currently constricted by the complexity of the parasite pools that can be generated, which in turn is limited both by transfection efficiency and by the relatively large differences in recombination rates of different *Plasmo*GEM vectors. We show that as a result of such heterogeneity, screening pools of mutants poses the risk of losing genotypes that are generated in small numbers due to inefficient recombination rates at a target locus, or because they have a reduced fitness. Such mutants get outcompeted by the faster-growing genotypes in the pool, but our pilot screen suggests that in many cases their presence and growth rates can be measured in a second-pass screen that excludes most of the faster-growing mutants. Once the vector-specific integration rates and fitness measurements are available for the entire vector resource, it will be possible to create more bespoke vector pools that are better matched for integration rates and fitness outcomes. This will enable the creation of larger pools of mutants, which can then be combined into even more complex superpools to empower truly genome-wide screens of parasite growth and differentiation using only a fraction of the number of rodents required today to study much smaller gene sets. We anticipate that these global genetic barseq screens will make it

possible to identify systematically the parasite metabolic pathways required for replication of asexual blood stages. Dropout screens that ask which barcodes are lost from the sexual parasite stages may generate lists of candidate genes required for parasite transmission to the mosquito, and similar screens can be designed to get at genes required for virulence or for efficient replication in normocytes versus reticulocytes. In each case, vectors from the *Plasmo*GEM resource will be available for validation and follow-up investigations.

EXPERIMENTAL PROCEDURES

Recombinase-Mediated Engineering Pipeline

The *Plasmo*GEM vector resource was created by recombinase-mediated engineering in continuous liquid culture on 96-well plates largely as described (Pfander et al., 2013), but with the following modifications. To improve genome coverage, the arrayed *P. berghei* genomic DNA library in the pJAZZ-OK linear plasmid (Lucigen) that provides the starting point for vector production was doubled in size to ~10,000 clones. The PCR product consisting of a *zeo-pheS* marker and 50 bp primer extensions homologous to the gene of interest was purified using the High Pure 96 UF Cleanup Kit (Roche) to improve lambda Red recombination efficiency in *E. coli*. The resulting intermediate vectors were selected in liquid culture containing 30 μg/mL kanamycin and 50 μg/mL zeocin. Kanamycin selection improved the proportion of intact intermediate vectors by selecting for the short arm of the pJAZZ plasmid. After two rounds of antibiotic selection, cultures were reinoculated into fresh selective medium and incubated for no longer than 16 hr at 37°C to favor the correct recombination product. Intermediate vector DNA was obtained using a QIAGEN Plasmid Plus 96 Miniprep kit and eluted into 65 μl Tris-EDTA buffer. DNA purity proved critical for the Gateway reaction, which was set up in 20 μl using 2 μl LR clonase (Invitrogen), 100 ng Gateway entry plasmid (e.g., pR6K-attL1-3xHA-*hdhfr-yfcu*-attL2), LR clonase buffer, and 300 ng purified intermediate vector. Gateway reactions were purified using the High Pure 96 UF Cleanup Kit. Electrocompetent *E. coli* TSA were transformed and plated on YEG-Cl agar containing kanamycin, and four colonies were picked to verify the sequence of their homology arms.

Computational Vector Design

A suite of software tools for automated vector design was created to work with the arrayed *E. coli* library of mapped *P. berghei* ANKA gDNA clones and has been described elsewhere (Schwach et al., 2015). In brief, the *Plasmo*GEM software tools select the most suitable library clone for each gene and type of modification and picks two unique 50 bp regions for lambda Red recombination, designated recUp and recDown, respectively. Together, these sites define the boundaries of the genomic region that is replaced by the selection marker cassette. A set of three PCR primers for quality-control purposes is also generated. For lambda Red recombination two highly unique 50 bp regions (*recUp* and *recDown*) were chosen on each selected library clone. In the case of knockout constructs these were designed to introduce the maximal deletion of the target gene compatible with a homology arm length of ≥1 kb, while leaving at least 1 kb upstream and 0.8 kb downstream of neighboring genes intact. For C-terminal tagging vectors *recUp* and *recDown* were chosen to delete only the stop codon from the gene of interest. *recUp* and *recDown* sequences were included in oligonucleotides for lambda Red recombination, one of which also carried a 10–11 bp gene-specific barcode assigned automatically from a list of optimized sequences, and an 18 bp constant primer annealing site to read out the barcode after integration into the *P. berghei* genome. The software also automatically designed oligonucleotides for quality control during vector production (QCR1 and QCR2) and a unique oligonucleotide annealing to the *P. berghei* genome at least 200 bp outside of the boundary of the shorter homology arm (GT) for PCR genotyping transgenic parasites. Vector designs, primer, and barcode sequences can be viewed in a searchable database at http://plasmogem.sanger.ac.uk (Schwach et al., 2015). For quality control, up to four colonies per final vector were batch sequenced on an Illumina MiSeq instrument. Sequencing libraries were made essentially as described (Quail et al., 2012).

New Tagging and Selection Cassettes

Gateway entry sclones were created for C-terminal tagging with fluorescent proteins by replacing the default 3xHA sequence in plasmid pR6K-attL1-3xHA-hdhfr-yfcu-attL2 (Pfander et al., 2013) with GFP-mu3 (Addgene plasmid 20410) (Franke-Fayard et al., 2004), iLov (Addgene plasmid 26769) (Chapman et al., 2008), mEmerald (a kind gift from J. Liu), mCherry (from RMgmDB plasmid pL0046), and mVenus. Entry clones with R6K origins were maintained in *E. coli* PIR2 (Invitrogen).

Parasites, Animals, Vector Preparation, and Transfection

Transgenic *P. berghei* were generated either in wild-type strain ANKA 2.34 or in a selectable marker-free reporter strain of *P. berghei* ANKA cl15cy1 expressing the mu3 variant of green fluorescent protein clone RMgm-7 (Franke-Fayard et al., 2004). Parasites were routinely propagated in 6- to 8-week-old Theiler's original (TO) outbred mice. Schizonts for barcode counting experiments were produced in female Wistar rats (200–250 g) to achieve maximal transfection efficiency. To generate pools of mutants for phenotyping by barcode counting, equal amounts of each vector were combined and the mixture digested with NotI to release the targeting vectors from the bacterial vector arms. A total of 5-8 μl of the digested vector mix, typically containing 100 ng of DNA for each vector, was used per transfection. Experiments with single vectors used 2 μg of *Not*I-restricted DNA per transfection. *Plasmo*GEM identification numbers for vectors used in this study are listed in Table S2. Transfections were done by electroporation of purified schizonts as described (Janse et al., 2006b), with the following modifications. Parasites for the schizont culture were from female Wistar rats (200–250 g) to achieve maximal transfection efficiency and were cultured for 21 hr before schizonts were isolated on a 55% Nycodenz/PBS cushion. Isolated schizonts were washed in complete media and electroporated using the 4D Nucleofector System (Lonza) in 16-well strips according to the pulse program FI-115 (see Supplemental Information, Protocol 1, for more detail). Growth rate phenotyping of transfected parasites was done in 6- to 8-week-old Balb/c inbred mice. Rodents were from Harlan, UK. All animal research was conducted under licenses from the UK Home Office and used protocols approved by the ethics committee of the Wellcome Trust Sanger Institute.

Growth Rate Phenotyping by Barcode Counting

Three batches of schizonts were transfected with the same vector pool and each injected intravenously into a different Balb/c mouse. Resistant parasites were selected by pyrimethamine (70 mg/L in the drinking water). Infections were monitored daily using Giemsa-stained thin blood films. A total of 30 μl of infected blood was collected from the tail at exactly the same time on days 4–8 posttransfection and diluted in 200 μl of phosphate-buffered saline. Total DNA was extracted from each sample and resuspended in water (50 μl on days 4–6 posttransfection, 100 μl for later days) using Supplemental Protocol 2. To sequence the vector-specific barcodes, 1 μl of each DNA sample served as template for a PCR reaction using Advantage 2 Taq polymerase (Clontech) with primers arg444 and arg445 (1× 95°C/5 min denaturation, 35 × 95°C/30 s, 55°C/20 s, 68°C/8 s, 1 × 10 min at 68°C), which bind to constant annealing sites flanking each barcode. The 167 bp amplicon was further extended in a second PCR reaction using oligonucleotides that in their 5′ extensions introduce Illumina adaptors and sample-specific barcodes (Table S3) for multiplexing up to 32 samples in one run of a MiSeq instrument. For sample-specific indexing, 5 μl of the first amplicon served as template for a further ten amplification cycles (1 × 95°C/2 min, 10 × 95°C/30 s, 68°C/15 s, 1 × 5 min, 68°C) using one generic oligonucleotide (PE1.0) and one of a set of 32 sample-specific indexing oligonucleotide. A total of 100 ng of each sequencing library was pooled and quality controlled by quantitative PCR for the presence of sequencing adaptors. Libraries were sequenced using MiSeq Reagent Kit v2 (300 cycle) from Illumina (MS-102-2002). In some experiments we compared PCR-mediated indexing to adaptor ligation libraries.

Due to their low complexity, PCR amplicon libraries had to be diluted to 4 nM before loading the flow cell of a MiSeq instrument (Illumina) at low cluster density (4×10^5 clusters/mm^2) with 30%–50% of PhiX spike-in. Sequencing of 150 bp paired-ends yielded 1.0–1.5 × 10^5 reads on average for each of the 32 samples. Using a Perl script, barcode sequences were extracted from sequencer output, counted, and the relative abundance of each barcode within the pool determined. The quantitation was considered reliable for

barcodes accounting for at least 0.1% of all counts. Parasitaemia curves for these mutants were inferred from the relative abundance of each barcode and from the observed total parasitaemia as determined using a Giemsa stained thin blood film. The relative fitness (w) of a mutant represented by a barcode on a given day (d) was calculated according to Mani et al. (2008) by comparing the daily change in its relative abundance (A) to that of the reference genes ($A_{Ri} \ldots A_{Rn}$) with normal growth.

$$w_{gene\ d} = \frac{A_d}{A_{d-1}} \times \frac{\sum_{i=1}^{n} \frac{A_{Ri\ d-1}}{A_{Ri\ d}}}{n}$$

Statistical analyses compared each barcode against the normal-growth reference vectors using a two-tailed t test (unequal variance, p values adjusted according to the false discovery rate method). A given mutant was considered viable when consistent growth of its barcode was observed for all time points in at least two of three replicates.

Genotyping

To verify vector integration by diagnostic PCR on parasite genomic DNA, we designed a target gene-specific oligonucleotide to anneals to the chromosome just outside of the vector's homology arm (Table S2) and paired it with a primer annealing to the *hdhfr* cassette within the targeting vector (either arg216 or arg218, depending on the orientation of the selection cassette relative to the first oligonucleotide). Integration of the targeting construct into the correct chromosome was further investigated by Southern hybridization of chromosomes separated by pulsed-field gel electrophoresis (PFGE) as described previously (Pfander et al., 2011).

SUPPLEMENTAL INFORMATION

Supplemental Information includes four tables, five figures, and two Supplemental Protocols and can be found with this article at http://dx.doi.org/10.1016/j.chom.2015.01.014.

AUTHOR CONTRIBUTIONS

O.B. and J.C.R. directed the research. E.B. led the *Plasmo*GEM resource team with additional support from K.M. The development of barcode sequencing was led by A.R.G., who also carried out the validation experiments. F.S. constructed the *Plasmo*GEM software tools and database and provided computational support for the project. G.G., B.A., C.H., and A.R.G. produced *Plasmo*GEM vectors and provided technology development. O.B., J.C.R., E.B., F.S., A.R.G., M.A.Q., and C.P. designed and analyzed experiments. O.B. and A.R.G. wrote the paper with critical input from all authors.

ACKNOWLEDGMENTS

We thank Tom Metcalf for technical assistance, Mandy Sanders for coordinating sequencing experiments, Thomas Dan Otto for guidance on analysing whole-genome sequence data, and the Sanger Institute sequencing teams. A plasmid encoding Emerald fluorescent proteins was a kind gift from J. Liu. A.R.G received a EVIMalaR studentship funded through the European Community's Seventh Framework Programme (FP7/2007-2013) under grant agreement number 242095. Work at the Sanger Institute was funded by grants from the Wellcome Trust (098051) and the Medical Research Council (G0501670).

Received: October 20, 2014
Revised: December 1, 2014
Accepted: January 7, 2015
Published: February 26, 2015

REFERENCES

Armstrong, C.M., and Goldberg, D.E. (2007). An FKBP destabilization domain modulates protein levels in Plasmodium falciparum. Nat. Methods *4*, 1007–1009.

Balu, B., and Adams, J.H. (2006). Functional genomics of Plasmodium falciparum through transposon-mediated mutagenesis. Cell. Microbiol. *8*, 1529–1536.

Braks, J.A., Franke-Fayard, B., Kroeze, H., Janse, C.J., and Waters, A.P. (2006). Development and application of a positive-negative selectable marker system for use in reverse genetics in Plasmodium. Nucleic Acids Res. *34*, e39.

Chapman, S., Faulkner, C., Kaiserli, E., Garcia-Mata, C., Savenkov, E.I., Roberts, A.G., Oparka, K.J., and Christie, J.M. (2008). The photoreversible fluorescent protein iLOV outperforms GFP as a reporter of plant virus infection. Proc. Natl. Acad. Sci. USA *105*, 20038–20043.

Collins, C.R., Das, S., Wong, E.H., Andenmatten, N., Stallmach, R., Hackett, F., Herman, J.P., Müller, S., Meissner, M., and Blackman, M.J. (2013). Robust inducible Cre recombinase activity in the human malaria parasite Plasmodium falciparum enables efficient gene deletion within a single asexual erythrocytic growth cycle. Mol. Microbiol. *88*, 687–701.

Crabb, B.S., and Cowman, A.F. (1996). Characterization of promoters and stable transfection by homologous and nonhomologous recombination in Plasmodium falciparum. Proc. Natl. Acad. Sci. USA *93*, 7289–7294.

Dessens, J.T., Sidén-Kiamos, I., Mendoza, J., Mahairaki, V., Khater, E., Vlachou, D., Xu, X.J., Kafatos, F.C., Louis, C., Dimopoulos, G., and Sinden, R.E. (2003). SOAP, a novel malaria ookinete protein involved in mosquito midgut invasion and oocyst development. Mol. Microbiol. *49*, 319–329.

Droucheau, E., Primot, A., Thomas, V., Mattei, D., Knockaert, M., Richardson, C., Sallicandro, P., Alano, P., Jafarshad, A., Baratte, B., et al. (2004). Plasmodium falciparum glycogen synthase kinase-3: molecular model, expression, intracellular localisation and selective inhibitors. Biochim. Biophys. Acta *1697*, 181–196.

Franke-Fayard, B., Trueman, H., Ramesar, J., Mendoza, J., van der Keur, M., van der Linden, R., Sinden, R.E., Waters, A.P., and Janse, C.J. (2004). A Plasmodium berghei reference line that constitutively expresses GFP at a high level throughout the complete life cycle. Mol. Biochem. Parasitol. *137*, 23–33.

Frénal, K., Tay, C.L., Mueller, C., Bushell, E.S., Jia, Y., Graindorge, A., Billker, O., Rayner, J.C., and Soldati-Favre, D. (2013). Global analysis of apicomplexan protein S-acyl transferases reveals an enzyme essential for invasion. Traffic *14*, 895–911.

Ghorbal, M., Gorman, M., Macpherson, C.R., Martins, R.M., Scherf, A., and Lopez-Rubio, J.J. (2014). Genome editing in the human malaria parasite Plasmodium falciparum using the CRISPR-Cas9 system. Nat. Biotechnol. *32*, 819–821.

Giaever, G., Chu, A.M., Ni, L., Connelly, C., Riles, L., Véronneau, S., Dow, S., Lucau-Danila, A., Anderson, K., André, B., et al. (2002). Functional profiling of the Saccharomyces cerevisiae genome. Nature *418*, 387–391.

Godiska, R., Mead, D., Dhodda, V., Wu, C., Hochstein, R., Karsi, A., Usdin, K., Entezam, A., and Ravin, N. (2010). Linear plasmid vector for cloning of repetitive or unstable sequences in Escherichia coli. Nucleic Acids Res. *38*, e88.

Goldfless, S.J., Wagner, J.C., and Niles, J.C. (2014). Versatile control of Plasmodium falciparum gene expression with an inducible protein-RNA interaction. Nat. Commun. *5*, 5329.

Guler, J.L., Freeman, D.L., Ahyong, V., Patrapuvich, R., White, J., Gujjar, R., Phillips, M.A., DeRisi, J., and Rathod, P.K. (2013). Asexual populations of the human malaria parasite, Plasmodium falciparum, use a two-step genomic strategy to acquire accurate, beneficial DNA amplifications. PLoS Pathog. *9*, e1003375.

Hensel, M., Shea, J.E., Gleeson, C., Jones, M.D., Dalton, E., and Holden, D.W. (1995). Simultaneous identification of bacterial virulence genes by negative selection. Science *269*, 400–403.

Janse, C.J., Franke-Fayard, B., Mair, G.R., Ramesar, J., Thiel, C., Engelmann, S., Matuschewski, K., van Gemert, G.J., Sauerwein, R.W., and Waters, A.P. (2006a). High efficiency transfection of Plasmodium berghei facilitates novel selection procedures. Mol. Biochem. Parasitol. *145*, 60–70.

Janse, C.J., Ramesar, J., and Waters, A.P. (2006b). High-efficiency transfection and drug selection of genetically transformed blood stages of the rodent malaria parasite Plasmodium berghei. Nat. Protoc. *1*, 346–356.

Jebiwott, S., Govindaswamy, K., Mbugua, A., and Bhanot, P. (2013). Plasmodium berghei calcium dependent protein kinase 1 is not required for host cell invasion. PLoS ONE *8*, e79171.

Kato, N., Sakata, T., Breton, G., Le Roch, K.G., Nagle, A., Andersen, C., Bursulaya, B., Henson, K., Johnson, J., Kumar, K.A., et al. (2008). Gene expression signatures and small-molecule compounds link a protein kinase to Plasmodium falciparum motility. Nat. Chem. Biol. *4*, 347–356.

Langridge, G.C., Phan, M.D., Turner, D.J., Perkins, T.T., Parts, L., Haase, J., Charles, I., Maskell, D.J., Peters, S.E., Dougan, G., et al. (2009). Simultaneous assay of every Salmonella Typhi gene using one million transposon mutants. Genome Res. *19*, 2308–2316.

LaRonde, N.A. (2014). The ancient microbial RIO kinases. J. Biol. Chem. *289*, 9488–9492.

Mani, R., St Onge, R.P., Hartman, J.L., 4th, Giaever, G., and Roth, F.P. (2008). Defining genetic interaction. Proc. Natl. Acad. Sci. USA *105*, 3461–3466.

Mazurkiewicz, P., Tang, C.M., Boone, C., and Holden, D.W. (2006). Signature-tagged mutagenesis: barcoding mutants for genome-wide screens. Nat. Rev. Genet. *7*, 929–939.

Ni, J.Q., Zhou, R., Czech, B., Liu, L.P., Holderbaum, L., Yang-Zhou, D., Shim, H.S., Tao, R., Handler, D., Karpowicz, P., et al. (2011). A genome-scale shRNA resource for transgenic RNAi in Drosophila. Nat. Methods *8*, 405–407.

Oppenheim, R.D., Creek, D.J., Macrae, J.I., Modrzynska, K.K., Pino, P., Limenitakis, J., Polonais, V., Seeber, F., Barrett, M.P., Billker, O., et al. (2014). BCKDH: the missing link in apicomplexan mitochondrial metabolism is required for full virulence of Toxoplasma gondii and Plasmodium berghei. PLoS Pathog. *10*, e1004263.

Otto, T.D., Böhme, U., Jackson, A.P., Hunt, M., Franke-Fayard, B., Hoeijmakers, W.A., Religa, A.A., Robertson, L., Sanders, M., Ogun, S.A., et al. (2014). A comprehensive evaluation of rodent malaria parasite genomes and gene expression. BMC Biol. *12*, 86.

Pfander, C., Anar, B., Schwach, F., Otto, T.D., Brochet, M., Volkmann, K., Quail, M.A., Pain, A., Rosen, B., Skarnes, W., et al. (2011). A scalable pipeline for highly effective genetic modification of a malaria parasite. Nat. Methods *8*, 1078–1082.

Pfander, C., Anar, B., Brochet, M., Rayner, J.C., and Billker, O. (2013). Recombination-mediated genetic engineering of Plasmodium berghei DNA. Methods Mol. Biol. *923*, 127–138.

Pino, P., Sebastian, S., Kim, E.A., Bush, E., Brochet, M., Volkmann, K., Kozlowski, E., Llinás, M., Billker, O., and Soldati-Favre, D. (2012). A tetracycline-repressible transactivator system to study essential genes in malaria parasites. Cell Host Microbe *12*, 824–834.

Quail, M.A., Smith, M., Coupland, P., Otto, T.D., Harris, S.R., Connor, T.R., Bertoni, A., Swerdlow, H.P., and Gu, Y. (2012). A tale of three next generation sequencing platforms: comparison of Ion Torrent, Pacific Biosciences and Illumina MiSeq sequencers. BMC Genomics *13*, 341.

Robinson, D.G., Chen, W., Storey, J.D., and Gresham, D. (2014). Design and analysis of Bar-seq experiments. G3 (Bethesda) *4*, 11–18.

Sarov, M., Schneider, S., Pozniakovski, A., Roguev, A., Ernst, S., Zhang, Y., Hyman, A.A., and Stewart, A.F. (2006). A recombineering pipeline for functional genomics applied to Caenorhabditis elegans. Nat. Methods *3*, 839–844.

Schwach, F., Bushell, E., Gomes, A.R., Anar, B., Girling, G., Herd, C., Rayner, J.C., and Billker, O. (2015). PlasmoGEM, a database supporting a community resource for large-scale experimental genetics in malaria parasites. Nucleic Acids Res. *43* (Database issue), D1176–D1182.

Sebastian, S., Brochet, M., Collins, M.O., Schwach, F., Jones, M.L., Goulding, D., Rayner, J.C., Choudhary, J.S., and Billker, O. (2012). A Plasmodium calcium-dependent protein kinase controls zygote development and transmission by translationally activating repressed mRNAs. Cell Host Microbe *12*, 9–19.

Skarnes, W.C., Rosen, B., West, A.P., Koutsourakis, M., Bushell, W., Iyer, V., Mujica, A.O., Thomas, M., Harrow, J., Cox, T., et al. (2011). A conditional knockout resource for the genome-wide study of mouse gene function. Nature *474*, 337–342.

Smith, A.M., Heisler, L.E., Mellor, J., Kaper, F., Thompson, M.J., Chee, M., Roth, F.P., Giaever, G., and Nislow, C. (2009). Quantitative phenotyping via deep barcode sequencing. Genome Res. *19*, 1836–1842.

Spaccapelo, R., Janse, C.J., Caterbi, S., Franke-Fayard, B., Bonilla, J.A., Syphard, L.M., Di Cristina, M., Dottorini, T., Savarino, A., Cassone, A., et al. (2010). Plasmepsin 4-deficient Plasmodium berghei are virulence attenuated and induce protective immunity against experimental malaria. Am. J. Pathol. *176*, 205–217.

Straimer, J., Lee, M.C., Lee, A.H., Zeitler, B., Williams, A.E., Pearl, J.R., Zhang, L., Rebar, E.J., Gregory, P.D., Llinás, M., et al. (2012). Site-specific genome editing in Plasmodium falciparum using engineered zinc-finger nucleases. Nat. Methods *9*, 993–998.

Suarez, C., Volkmann, K., Gomes, A.R., Billker, O., and Blackman, M.J. (2013). The malarial serine protease SUB1 plays an essential role in parasite liver stage development. PLoS Pathog. *9*, e1003811.

Tewari, R., Straschil, U., Bateman, A., Böhme, U., Cherevach, I., Gong, P., Pain, A., and Billker, O. (2010). The systematic functional analysis of Plasmodium protein kinases identifies essential regulators of mosquito transmission. Cell Host Microbe *8*, 377–387.

Tomas, A.M., Margos, G., Dimopoulos, G., van Lin, L.H., de Koning-Ward, T.F., Sinha, R., Lupetti, P., Beetsma, A.L., Rodriguez, M.C., Karras, M., et al. (2001). P25 and P28 proteins of the malaria ookinete surface have multiple and partially redundant functions. EMBO J. *20*, 3975–3983.

van Dijk, M.R., Janse, C.J., and Waters, A.P. (1996). Expression of a Plasmodium gene introduced into subtelomeric regions of Plasmodium berghei chromosomes. Science *271*, 662–665.

van Dijk, M.R., van Schaijk, B.C., Khan, S.M., van Dooren, M.W., Ramesar, J., Kaczanowski, S., van Gemert, G.J., Kroeze, H., Stunnenberg, H.G., Eling, W.M., et al. (2010). Three members of the 6-cys protein family of Plasmodium play a role in gamete fertility. PLoS Pathog. *6*, e1000853.

Wagner, J.C., Platt, R.J., Goldfless, S.J., Zhang, F., and Niles, J.C. (2014). Efficient CRISPR-Cas9-mediated genome editing in Plasmodium falciparum. Nat. Methods *11*, 915–918.

Winzeler, E.A., Shoemaker, D.D., Astromoff, A., Liang, H., Anderson, K., Andre, B., Bangham, R., Benito, R., Boeke, J.D., Bussey, H., et al. (1999). Functional characterization of the S. cerevisiae genome by gene deletion and parallel analysis. Science *285*, 901–906.

Wu, Y., Sifri, C.D., Lei, H.H., Su, X.Z., and Wellems, T.E. (1995). Transfection of Plasmodium falciparum within human red blood cells. Proc. Natl. Acad. Sci. USA *92*, 973–977.

Zhang, Y., Muyrers, J.P., Testa, G., and Stewart, A.F. (2000). DNA cloning by homologous recombination in Escherichia coli. Nat. Biotechnol. *18*, 1314–1317.

Zhang, M., Mishra, S., Sakthivel, R., Rojas, M., Ranjan, R., Sullivan, W.J., Jr., Fontoura, B.M., Ménard, R., Dever, T.E., and Nussenzweig, V. (2012). PK4, a eukaryotic initiation factor 2α(eIF2α) kinase, is essential for the development of the erythrocytic cycle of Plasmodium. Proc. Natl. Acad. Sci. USA *109*, 3956–3961.

Cell Host & Microbe, *17*

Supplemental Information

A Genome Scale Vector Resource Enables High-Throughput Reverse Genetic Screening in a Malaria Parasite

Ana Rita Gomes, Ellen Bushell, Frank Schwach, Gareth Girling, Burcu Anar, Michael A. Quail, Colin Herd, Claudia Pfander, Katarzyna Modrzynska, Julian C. Rayner, and Oliver Billker

Table S1. Targetability and fitness measurements for 40 eukaryotic protein kinase genes as determined by barcode sequencing, compared to data from a previous study by Tewari *et al.* (2010), which used conventional gene targeting. p values are adjusted for multiple testing. The table combines data from 5 different barseq screens. n = 6 for most fitness measurements, except for PBANKA_082960 (n = 3), *gsk3*, *kin* and PBANKA_082710 (n = 4). p. t. = post transfection. Related to Figs. 3 and 5.

				Day 5 p. t.			Day 6 p. t.			Day 7 p. t.			Day 8 p. t.				
P. berghei gene ID	Gene name	Tewari *et al.* 2010	This study	Fitness	SD	p	Fitness	SD	p	Fitness	SD	p	Fitness	SD	p	Average fitness, days 5-8	Assessment
PBANKA_135150	*cdpk5*	Possibly essential	Possibly essential	NA	NA	NA	NA	NA	NA	NA	NA	NA	NA	NA	NA	NA	Possibly essential
PBANKA_092520	*cdpk7*	Possibly essential	Possibly essential	NA	NA	NA	NA	NA	NA	NA	NA	NA	NA	NA	NA	NA	Possibly essential
PBANKA_031140		Possibly essential	Possibly essential	NA	NA	NA	NA	NA	NA	NA	NA	NA	NA	NA	NA	NA	Possibly essential
PBANKA_083560	*pka*	Possibly essential	Possibly essential	NA	NA	NA	NA	NA	NA	NA	NA	NA	NA	NA	NA	NA	Possibly essential
PBANKA_090380		Possibly essential	Possibly essential	NA	NA	NA	NA	NA	NA	NA	NA	NA	NA	NA	NA	NA	Possibly essential
PBANKA_091210	*ck1*	Possibly essential	Possibly essential	NA	NA	NA	NA	NA	NA	NA	NA	NA	NA	NA	NA	NA	Possibly essential
PBANKA_071730	*crk-3*	Possibly essential	Possibly essential	NA	NA	NA	NA	NA	NA	NA	NA	NA	NA	NA	NA	NA	Possibly essential
PBANKA_130920	*lammer/(CLK1)*	Possibly essential	Possibly essential	NA	NA	NA	NA	NA	NA	NA	NA	NA	NA	NA	NA	NA	Possibly essential
PBANKA_093300	*prk4*	Possibly essential	Possibly essential	NA	NA	NA	NA	NA	NA	NA	NA	NA	NA	NA	NA	NA	Possibly essential
PBANKA_080800	*crk-4*	Possibly essential	Possibly essential	NA	NA	NA	NA	NA	NA	NA	NA	NA	NA	NA	NA	NA	Possibly essential
PBANKA_090110		Possibly essential	Possibly essential	NA	NA	NA	NA	NA	NA	NA	NA	NA	NA	NA	NA	NA	Possibly essential
PBANKA_093860	*ck2*	Possibly essential	Possibly essential	NA	NA	NA	NA	NA	NA	NA	NA	NA	NA	NA	NA	NA	Possibly essential
PBANKA_101090	*tkl5*	Possibly essential	Possibly essential	NA	NA	NA	NA	NA	NA	NA	NA	NA	NA	NA	NA	NA	Possibly essential
PBANKA_144300	*nek-1*	Possibly essential	Possibly essential	NA	NA	NA	NA	NA	NA	NA	NA	NA	NA	NA	NA	NA	Possibly essential
PBANKA_135090	*pk6*	Possibly essential	Possibly essential	NA	NA	NA	NA	NA	NA	NA	NA	NA	NA	NA	NA	NA	Possibly essential
PBANKA_080560		Possibly essential	Possibly essential	NA	NA	NA	NA	NA	NA	NA	NA	NA	NA	NA	NA	NA	Possibly essential
PBANKA_122500	*fikk*	Possibly essential	Possibly essential	NA	NA	NA	NA	NA	NA	NA	NA	NA	NA	NA	NA	NA	Possibly essential
PBANKA_100820	*pkg*	Possibly essential	Possibly essential	NA	NA	NA	NA	NA	NA	NA	NA	NA	NA	NA	NA	NA	Possibly essential
PBANKA_112690	*pk4*	Possibly essential	Possibly essential	NA	NA	NA	NA	NA	NA	NA	NA	NA	NA	NA	NA	NA	Possibly essential
PBANKA_052140	*rio2*	Possibly essential	Possibly essential	NA	NA	NA	NA	NA	NA	NA	NA	NA	NA	NA	NA	NA	Possibly essential†□
PBANKA_040110	*srpk*	KO confirmed	Targetable	0.59	0.18	2.5E-02	0.65	0.24	1.3E-01	0.80	0.08	7.8E-02	0.83	0.15	2.1E-01	0.72	Confirmed KO
PBANKA_040820	*cdpk3*	KO confirmed	Targetable	1.00	0.09	1.0E+00	0.97	0.05	2.2E-01	1.01	0.07	7.6E-01	1.04	0.06	2.9E-01	1.01	Confirmed KO
PBANKA_040940	*pkrp*	KO confirmed	Targetable	0.98	0.21	9.2E-01	1.03	0.09	7.4E-01	0.98	0.09	9.0E-01	1.02	0.05	8.5E-01	1.00	Confirmed KO
PBANKA_061520	*cdpk4*	KO confirmed	Targetable	0.94	0.13	5.8E-01	0.89	0.10	4.1E-02	0.98	0.07	7.4E-01	0.81	0.08	1.4E-02	0.90	Confirmed KO
PBANKA_061670	*nek-4*	KO confirmed	Targetable	0.87	0.19	2.0E-01	1.00	0.16	9.7E-01	1.02	0.11	7.4E-01	1.02	0.16	8.5E-01	0.98	Confirmed KO
PBANKA_082710		KO confirmed	Targetable	0.38	0.22	2.5E-03	0.65	0.06	1.7E-04	0.91	0.08	2.9E-02	1.03	0.38	8.4E-01	0.74	Confirmed KO
PBANKA_092550	*cdpk6*	KO confirmed	Targetable	0.86	0.12	8.9E-02	0.90	0.05	8.7E-03	0.79	0.10	8.1E-03	0.97	0.12	6.8E-01	0.88	Confirmed KO
PBANKA_093370	*map-2*	KO confirmed	Targetable	0.99	0.11	9.5E-01	0.95	0.06	1.8E-01	1.05	0.06	2.1E-01	1.03	0.05	4.2E-01	1.00	Confirmed KO
PBANKA_101330	*map-1*	KO confirmed	Targetable	1.04	0.11	5.8E-01	0.92	0.04	4.1E-03	1.06	0.04	4.1E-02	1.04	0.04	8.5E-02	1.02	Confirmed KO
PBANKA_101980	*cdlk*	KO confirmed	Targetable	0.20	0.02	5.0E-06	0.53	0.13	1.7E-02	0.62	0.42	4.2E-01	1.13	0.19	8.0E-01	0.62	Confirmed KO
PBANKA_112270	*tkl4*	KO confirmed	Targetable	0.46	0.33	7.8E-02	0.28	0.11	2.9E-03	0.55	0.16	3.0E-02	0.69	0.11	6.2E-02	0.49	Confirmed KO
PBANKA_130690	*srpk2*	KO confirmed	Targetable	0.83	0.32	5.3E-01	1.00	0.09	9.7E-01	1.01	0.14	9.5E-01	1.05	0.09	3.9E-01	0.97	Confirmed KO
PBANKA_131800	*kin*	KO confirmed	Targetable	1.08	0.29	6.9E-01	0.99	0.10	1.9E-01	0.97	0.07	2.0E-01	1.00	0.11	8.4E-01	1.01	Confirmed KO
PBANKA_135260		KO confirmed	Targetable	0.88	0.27	5.8E-01	0.98	0.07	5.6E-01	1.00	0.09	9.5E-01	1.03	0.04	3.9E-01	0.97	Confirmed KO
PBANKA_146050	*gak*	KO confirmed	Targetable	0.83	0.12	8.9E-02	0.74	0.09	2.6E-03	0.83	0.05	1.4E-04	1.17	0.11	8.5E-02	0.89	Confirmed KO
PBANKA_030850	*tkl1*	Possibly essential	Targetable	0.86	0.28	5.3E-01	0.96	0.21	8.4E-01	1.14	0.18	1.7E-01	1.04	0.06	3.9E-01	1.00	New KO
PBANKA_031420	*cdpk1*	Possibly essential	Targetable §	0.95	0.14	5.5E-01	0.89	0.05	1.4E-03	1.10	0.05	2.7E-03	1.00	0.06	9.5E-01	0.98	New KO Ω
PBANKA_041040	*gsk-3*	Possibly essential	Targetable	0.99	0.03	8.2E-01	1.00	0.04	9.3E-01	1.05	0.05	5.6E-01	1.01	0.03	8.5E-01	1.01	New KO Ω
PBANKA_082960		Possibly essential	Targetable	1.03	0.04	7.5E-01	1.02	0.08	7.5E-01	1.01	0.06	7.5E-01	1.05	0.02	2.5E-02	1.03	New KO Ω
PBANKA_130520		Possibly essential	Targetable	1.02	0.12	9.5E-01	0.98	0.13	8.4E-01	1.01	0.10	8.3E-01	0.99	0.08	9.5E-01	1.00	New KO *
PBANKA_136210	*tkl3*	Possibly essential	Targetable	1.05	0.22	7.7E-01	0.99	0.09	9.6E-01	1.06	0.14	4.6E-01	0.92	0.09	2.0E-01	1.01	New KO Ω
PBANKA_141450		Possibly essential	Targetable	0.91	0.33	7.3E-01	1.00	0.16	9.7E-01	0.94	0.07	1.7E-01	0.99	0.10	8.5E-01	0.96	New KO *
PBANKA_142160		Possibly essential	Targetable	1.01	0.13	1.0E+00	0.93	0.07	3.4E-02	1.10	0.16	2.4E-01	1.01	0.12	9.5E-01	1.01	New KO *
PBANKA_144560	*rio1*	Possibly essential	Targetable	0.23	0.00	2.0E-03	0.67	0.04	1.1E-02	0.91	0.08	2.7E-01	0.94	0.01	5.9E-02	0.69	New KO ◊†
PBANKA_020580	*eik2; uis1*	KO confirmed	No integration	NA	NA	NA	NA	NA	NA	NA	NA	NA	NA	NA	NA	NA	False negative
PBANKA_031030	*pk7*	KO confirmed	No integration	NA	NA	NA	NA	NA	NA	NA	NA	NA	NA	NA	NA	NA	False negative
PBANKA_051490	*p28*		Targetable	0.92	0.15	5.3E-01	0.98	0.10	9.2E-01	0.98	0.05	7.4E-01	1.01	0.06	8.5E-01	0.97	Normal growth reference
PBANKA_051500	*p25*		Targetable	1.01	0.12	9.8E-01	1.03	0.02	1.0E-01	1.04	0.07	2.4E-01	0.98	0.04	3.9E-01	1.02	Normal growth reference
PBANKA_103780	*soap*		Targetable	1.00	0.08	1.0E+00	0.95	0.07	1.1E-01	1.06	0.05	1.1E-01	1.03	0.05	3.8E-01	1.01	Normal growth reference
PBANKA_030600	*p230p*		Taggable	1.07	0.12	5.8E-01	1.04	0.07	4.7E-01	0.91	0.03	8.1E-03	0.98	0.09	8.5E-01	1.00	Normal growth reference
PBANKA_140160			Targetable	0.51	0.12	1.1E-04	0.46	0.06	1.3E-05	0.58	0.07	2.1E-05	0.87	0.07	1.4E-02	0.60	Attenuated reference
PBANKA_110420	*bckdh e1b*		Targetable	0.74	0.12	6.9E-03	0.58	0.07	1.3E-05	0.67	0.05	2.8E-06	0.61	0.06	6.6E-05	0.65	Attenuated reference
PBANKA_103440	*pm4*		Targetable	0.79	0.20	8.9E-02	0.73	0.05	1.3E-05	0.71	0.12	4.3E-03	0.70	0.08	2.2E-03	0.73	Attenuated reference

* PCR genotyping evidence for targetability but no independent clone generated.
§ This study and Jebiwott *et al.*, 2013
† Confirmed by WGS of uncloned population
□ Confirmed by selection for target duplication
Ω Confirmed by genotyped clone
◊ Confirmed by PFGE analysis of uncloned population

Table S2. Gene identification numbers (ID), *Plasmo*GEM vector IDs and gene specific genotyping primers. Related to Figs. 3-4 and Fig. S1.

Gene ID	*Plasmo*GEM ID	Gene name	Gene specific primer	Sequence	Pairs with
p230p-tag	PbGEM-226060	p230p-tag	arg00448	GGAACAATATGGCTGTTCAATG	arg00218
PBANKA_051490	PbGEM-015545	28 kDa ookinete surface protein (P28)	arg00447	GGATTCCGTGAATGATCCCC	arg00216
PBANKA_051500	PbGEM-015561	25 kDa ookinete surface antigen precursor (P25)	arg00449	TGTTCCCGTTGTAACAGTGCA	arg00216
PBANKA_103780	PbGEM-097822	secreted ookinete adhesive protein (SOAP)	arg00446	TTTCCCACTGCGTACCCTTT	arg00218
PBANKA_103440	PbGEM-039254	plasmepsin IV (PM4)	arg00452	AGACAAACTTTGCCCACAACA	arg00216
PBANKA_110420	PbGEM-122074	3-methyl-2-oxobutanoate dehydrogenase (lipoamide), putative	arg00451	AAAGCCAGAAACGACATGAA	arg00218
PBANKA_140160	PbGEM-062476	methyl transferase-like protein, putative	arg00450	CATGGCTATGACCGACAGAG	arg00218
PBANKA_020580	PbGEM-082161	serine/threonine protein kinase, putative (IK2)	arg00471	CGAAGCGCTTTACCATGTGGG	arg00218
PBANKA_030850	PbGEM-009884	protein kinase, putative (TKL1)	arg00472	AGTGCATACGCTTCATGACGCT	arg00216
PBANKA_031030	PbGEM-072470	protein kinase 7 (PK7)	arg00473	AACCGAAGTGCTCTTTGCGA	arg00216
PBANKA_031140	PbGEM-111762	serine/threonine protein kinase, putative	arg00474	TGCTACCTTACGCATTGGACA	arg00218
PBANKA_031420	PbGEM-010677	calcium dependent protein kinase 1 (CDPK1)	arg00371	CCGGTATTTATCATCCAAGAG	arg00216
PBANKA_040110	PbGEM-084034	serine/threonine protein kinase, putative (SRPK1)	arg00475	TGATGCGGATTTGTGTGTGT	arg00218
PBANKA_040820	PbGEM-111826	calcium dependent protein kinase 3 (CDPK3)	arg00386	GGTTATCTATACATTTATTTGTG	arg00218
PBANKA_040940	PbGEM-111754	protein kinase, putative (PKRP)	arg00476	AGCAATGATGTAGGATGTGCA	arg00216
PBANKA_052140	PbGEM-072474	RIO-like serine/threonine kinase, putative	arg00477	TCCAAAGCGTTATGCCAAGTGT	arg00218
PBANKA_061520	PbGEM-087803	calcium dependent protein kinase 4 (CDPK4)	arg00255	GGGGGTTTGTGTGGAGGCG	arg00216
PBANKA_061670	PbGEM-111690	NIMA related kinase 4 (NEK4)	arg00234	GCACACTCACCTGAAATGTCG	arg00216
PBANKA_071730	PbGEM-072538	cdc2-related protein kinase 3 (CRK3)	arg00478	TGGTTCAATTGTTGAGCAAAGT	arg00218
PBANKA_080560	PbGEM-072522	O-sialoglycoprotein endopeptidase, putative	arg00479	TCGGAAAAACCTTGAAAGCGC	arg00218
PBANKA_080800	PbGEM-111786	cdc2-related protein kinase 4 (CRK4)	arg00480	TCGCTAGTTATATATGCTCACGC	arg00216
PBANKA_083560	PbGEM-028140	cAMP-dependent protein kinase catalytic subunit (PKAc)	arg00482	TCAAGTGAAACGGAATAGAAG	arg00218
PBANKA_090110	PbGEM-111746	protein kinase, putative	arg00483	TCAGAAAGGTATACGTCAACGG	arg00216
PBANKA_090380	PbGEM-111794	serine/threonine protein kinase, putative	arg00484	AGCTTGTATGTCGATTCGAGA	arg00218
PBANKA_091210	PbGEM-111738	casein kinase 1 (CK1)	arg00485	ACGATGTGTGCAGCAGGTCT	arg00216
PBANKA_092520	PbGEM-093973	calcium-dependent protein kinase 7 (CDPK7)	arg00486	TGTCTCCCTAAAAGGCATGTGC	arg00218
PBANKA_092550	PbGEM-111850	calcium dependent protein kinase 6 (CDPK6)	arg00381	TGCACTTCACCAAAAGCGCCA	arg00218
PBANKA_093300	PbGEM-072518	serine/threonine protein kinase, puative	arg00487	AGCAGTGCACACAAAAGAAGA	arg00218
PBANKA_093370	PbGEM-111778	mitogen-activated protein kinase 2 (MAP2)	arg00256	ACCATGAGTGCATGCATAGGA	arg00216
PBANKA_101330	PbGEM-036210	mitogen-activated protein kinase 1 (MAP1)	arg00453	CGCGTGGAAAACTGTGGGC	arg00216
PBANKA_101980	PbGEM-111858	serine/threonine protein kinase, putative	arg00488	TGCCCGGAATGCACATATGTTG	arg00216
PBANKA_112270	PbGEM-111714	protein kinase, putative (TKL4)	arg00489	TGGGGAGTACCTTGCCCATGCA	arg00216
PBANKA_112690	PbGEM-099789	protein kinase PK4 (PK4)	arg00490	AGTATTGCCCCATCCATTGCT	arg00218
PBANKA_122500	PbGEM-111674	serine/threonine protein kinase, FIKK family	arg00491	TGTCTGACTCTCCATGGTGTCCC	arg00218
PBANKA_130520	PbGEM-053796	serine/threonine protein kinase, putative	arg00456	GAGTACCTGTTGGTCACGC	arg00216
PBANKA_130690	PbGEM-104812	serine/threonine protein kinase, putative (SRPK2)	arg00225	TGCCCTTTTGATGCCAAGACG	arg00216
PBANKA_130920	PbGEM-104970	serine/threonine kinase-1, putative	arg00492	TCACGCATCGGGGATTTGTCA	arg00216
PBANKA_131800	PbGEM-105530	serine/threonine protein kinase, putative	arg00493	ACGGAGCACAATGTATGCCATG	arg00218
PBANKA_135150	PbGEM-111682	calcium dependent protein kinase 5 (CDPK5)	arg00494	TCGACGGTACTGTCTGACTGGT	arg00216
PBANKA_135260	PbGEM-111802	serine/threonine protein kinase, putative	arg00259	CTGGCGCACGGCAAAACCC	arg00218
PBANKA_136210	PbGEM-108848	protein kinase, putative	arg00262	ACGACAATGTGCATGCCTCA	arg00218
PBANKA_141450	PbGEM-111842	protein kinase, putative	arg00272	CCACAAAGCAATTCCGGTGC	arg00218
PBANKA_142160	PbGEM-065291	calcium/calmodulin-dependent protein kinase, putative	arg00454	TCTAAATCGCGGCTTTCACA	arg00218
PBANKA_144560	PbGEM-111706	protein kinase, putative	arg00495	TGCTCAAGCAACAGCAGGACA	arg00216
PBANKA_146050	PbGEM-072542	serine/threonine protein kinase, putative	arg00173	CCTGGAATTGTTTCCCCACAC	arg00218

Table S3. Oligonucleotide used for barcode sequencing. Related to Fig. 3 and S1.

Name	Sequence
arg00444	TCGGCATTCCTGCTGAACCGCTCTTCCGATCTGTAATTCGTGCGCGTCAG
arg00445	ACACTCTTTCCCTACACGACGCTCTTCCGATCTCCTTCAATTTCGATGGGTAC
PE1.0	AATGATACGGCGACCACCGAGATCTACACTCTTTCCCTACACGACGCTCTTCCGATC*T 1)
iPCRindex1	CAAGCAGAAGACGGCATACGAGATTGCTAATCACTGAGATCGGTCTCGGCATTCCTGCTGAACCGCTCTTCCGATC*T
iPCRindex2	CAAGCAGAAGACGGCATACGAGATTAGGGGGATTCGAGATCGGTCTCGGCATTCCTGCTGAACCGCTCTTCCGATC*T
iPCRindex3	CAAGCAGAAGACGGCATACGAGATAGTTTCCCAGGGAGATCGGTCTCGGCATTCCTGCTGAACCGCTCTTCCGATC*T
iPCRindex4	CAAGCAGAAGACGGCATACGAGATCCTGGGAGGTAGAGATCGGTCTCGGCATTCCTGCTGAACCGCTCTTCCGATC*T
iPCRindex5	CAAGCAGAAGACGGCATACGAGATATACCACAAATGAGATCGGTCTCGGCATTCCTGCTGAACCGCTCTTCCGATC*T
iPCRindex6	CAAGCAGAAGACGGCATACGAGATGATCTCTCGGGGAGATCGGTCTCGGCATTCCTGCTGAACCGCTCTTCCGATC*T
iPCRindex7	CAAGCAGAAGACGGCATACGAGATACCCTATACTCGAGATCGGTCTCGGCATTCCTGCTGAACCGCTCTTCCGATC*T
iPCRindex8	CAAGCAGAAGACGGCATACGAGATCTCAATTAAGAGAGATCGGTCTCGGCATTCCTGCTGAACCGCTCTTCCGATC*T
iPCRindex9	CAAGCAGAAGACGGCATACGAGATCGACAGAACGTGAGATCGGTCTCGGCATTCCTGCTGAACCGCTCTTCCGATC*T
iPCRindex10	CAAGCAGAAGACGGCATACGAGATTCGCCATTATGGAGATCGGTCTCGGCATTCCTGCTGAACCGCTCTTCCGATC*T
iPCRindex11	CAAGCAGAAGACGGCATACGAGATATGTTCCGGCCGAGATCGGTCTCGGCATTCCTGCTGAACCGCTCTTCCGATC*T
iPCRindex12	CAAGCAGAAGACGGCATACGAGATTCTTGAAGTGAGAGATCGGTCTCGGCATTCCTGCTGAACCGCTCTTCCGATC*T
iPCRindex13	CAAGCAGAAGACGGCATACGAGATGAAGGCCAGCTGAGATCGGTCTCGGCATTCCTGCTGAACCGCTCTTCCGATC*T
iPCRindex14	CAAGCAGAAGACGGCATACGAGATCCAATGTGCAGGAGATCGGTCTCGGCATTCCTGCTGAACCGCTCTTCCGATC*T
iPCRindex15	CAAGCAGAAGACGGCATACGAGATATCGAAGGACCGAGATCGGTCTCGGCATTCCTGCTGAACCGCTCTTCCGATC*T
iPCRindex16	CAAGCAGAAGACGGCATACGAGATTCGGGTGCGAAGAGATCGGTCTCGGCATTCCTGCTGAACCGCTCTTCCGATC*T
iPCRindex17	CAAGCAGAAGACGGCATACGAGATGTAATTTACGGGAGATCGGTCTCGGCATTCCTGCTGAACCGCTCTTCCGATC*T
iPCRindex18	CAAGCAGAAGACGGCATACGAGATATATCGACTACGAGATCGGTCTCGGCATTCCTGCTGAACCGCTCTTCCGATC*T
iPCRindex19	CAAGCAGAAGACGGCATACGAGATTGATTCTTACAGAGATCGGTCTCGGCATTCCTGCTGAACCGCTCTTCCGATC*T
iPCRindex20	CAAGCAGAAGACGGCATACGAGATACGGCGGGCCTGAGATCGGTCTCGGCATTCCTGCTGAACCGCTCTTCCGATC*T
iPCRindex21	CAAGCAGAAGACGGCATACGAGATCTTGCGTGGAGGAGATCGGTCTCGGCATTCCTGCTGAACCGCTCTTCCGATC*T
iPCRindex22	CAAGCAGAAGACGGCATACGAGATTAATCAAAGACGAGATCGGTCTCGGCATTCCTGCTGAACCGCTCTTCCGATC*T
iPCRindex23	CAAGCAGAAGACGGCATACGAGATGGCGGGCTCTAGAGATCGGTCTCGGCATTCCTGCTGAACCGCTCTTCCGATC*T
iPCRindex24	CAAGCAGAAGACGGCATACGAGATCCTCCATTTCTGAGATCGGTCTCGGCATTCCTGCTGAACCGCTCTTCCGATC*T
iPCRindex25	CAAGCAGAAGACGGCATACGAGATAACCAGCGCTGGAGATCGGTCTCGGCATTCCTGCTGAACCGCTCTTCCGATC*T
iPCRindex26	CAAGCAGAAGACGGCATACGAGATTATTCGTCAACGAGATCGGTCTCGGCATTCCTGCTGAACCGCTCTTCCGATC*T
iPCRindex27	CAAGCAGAAGACGGCATACGAGATGCGCTGATGCAGAGATCGGTCTCGGCATTCCTGCTGAACCGCTCTTCCGATC*T
iPCRindex28	CAAGCAGAAGACGGCATACGAGATCTCATATGGCTGAGATCGGTCTCGGCATTCCTGCTGAACCGCTCTTCCGATC*T
iPCRindex29	CAAGCAGAAGACGGCATACGAGATACAGGGGCAGGGAGATCGGTCTCGGCATTCCTGCTGAACCGCTCTTCCGATC*T
iPCRindex30	CAAGCAGAAGACGGCATACGAGATGGTTTTATACCGAGATCGGTCTCGGCATTCCTGCTGAACCGCTCTTCCGATC*T
iPCRindex31	CAAGCAGAAGACGGCATACGAGATGCATGACTTTAGAGATCGGTCTCGGCATTCCTGCTGAACCGCTCTTCCGATC*T
iPCRindex32	CAAGCAGAAGACGGCATACGAGATTTCTGAGTTCTGAGATCGGTCTCGGCATTCCTGCTGAACCGCTCTTCCGATC*T

1) © 2006-2008 Illumina, Inc. All rights reserved

Figure S1

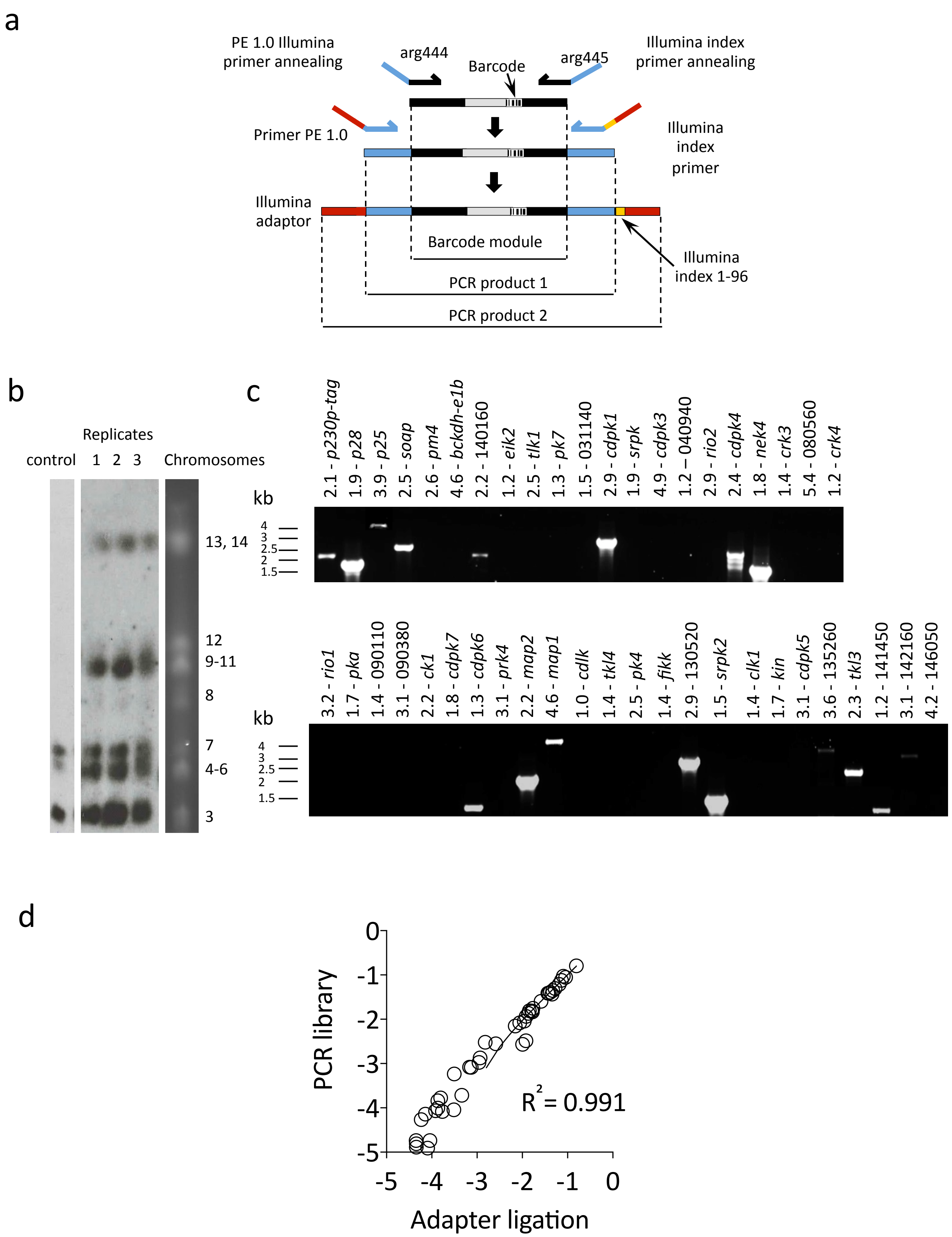
a
PE 1.0 Illumina primer annealing
arg444
Barcode
arg445
Illumina index primer annealing
Primer PE 1.0
Illumina index primer
Illumina adaptor
Barcode module
PCR product 1
PCR product 2
Illumina index 1-96
b
Replicates
control 1 2 3
Chromosomes
13, 14
12
9-11
8
7
4-6
3
c
kb
4
3
2.5
2
1.5
2.1 - p230p-tag
1.9 - p28
3.9 - p25
2.5 - soap
2.6 - pm4
4.6 - bckdh-e1b
2.2 - 140160
1.2 - eik2
2.5 - tlk1
1.3 - pk7
1.5 - 031140
2.9 - cdpk1
1.9 - srpk
4.9 - cdpk3
1.2 – 040940
2.9 - rio2
2.4 - cdpk4
1.8 - nek4
1.4 - crk3
5.4 - 080560
1.2 - crk4
3.2 - rio1
1.7 - pka
1.4 - 090110
3.1 - 090380
2.2 - ck1
1.8 - cdpk7
1.3 - cdpk6
3.1 - prk4
2.2 - map2
4.6 - map1
1.0 - cdlk
1.4 - tkl4
2.5 - pk4
1.4 - fikk
2.9 - 130520
1.5 - srpk2
1.4 - clk1
1.7 - kin
3.1 - cdpk5
3.6 - 135260
2.3 - tkl3
1.2 - 141450
3.1 - 142160
4.2 - 146050
d
PCR library
Adapter ligation
R² = 0.991
0
-1
-2
-3
-4
-5

Figure S2

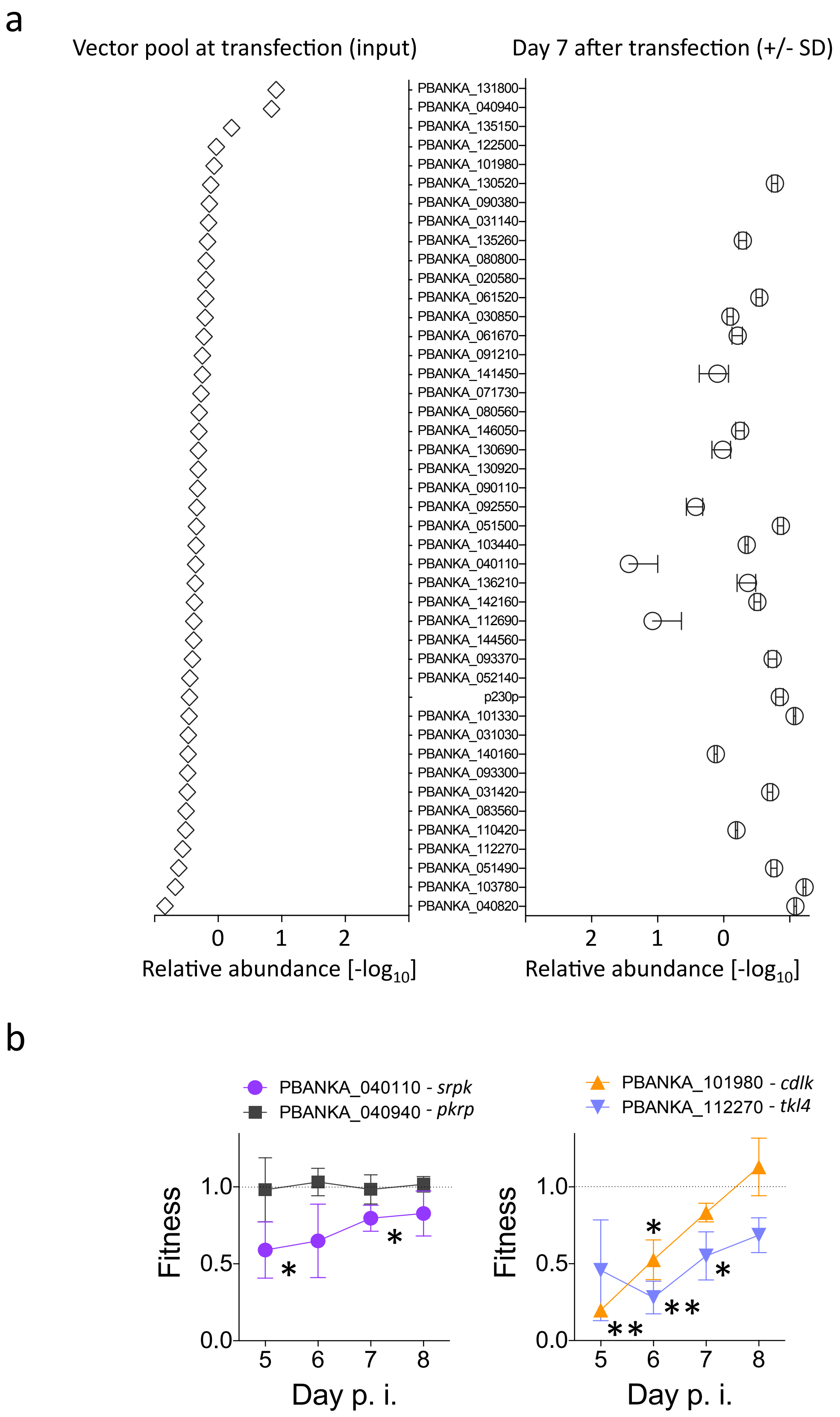
a
Vector pool at transfection (input)
Day 7 after transfection (+/- SD)
PBANKA_131800
PBANKA_040940
PBANKA_135150
PBANKA_122500
PBANKA_101980
PBANKA_130520
PBANKA_090380
PBANKA_031140
PBANKA_135260
PBANKA_080800
PBANKA_020580
PBANKA_061520
PBANKA_030850
PBANKA_061670
PBANKA_091210
PBANKA_141450
PBANKA_071730
PBANKA_080560
PBANKA_146050
PBANKA_130690
PBANKA_130920
PBANKA_090110
PBANKA_092550
PBANKA_051500
PBANKA_103440
PBANKA_040110
PBANKA_136210
PBANKA_142160
PBANKA_112690
PBANKA_144560
PBANKA_093370
PBANKA_052140
p230p
PBANKA_101330
PBANKA_031030
PBANKA_140160
PBANKA_093300
PBANKA_031420
PBANKA_083560
PBANKA_110420
PBANKA_112270
PBANKA_051490
PBANKA_103780
PBANKA_040820
0 1 2
Relative abundance [-log10]
2 1 0
Relative abundance [-log10]
b
PBANKA_040110 - srpk
PBANKA_040940 - pkrp
Fitness
1.0
0.5
0.0
5 6 7 8
Day p. i.
PBANKA_101980 - cdlk
PBANKA_112270 - tkl4
Fitness
1.0
0.5
0.0
5 6 7 8
Day p. i.

Figure S3

a

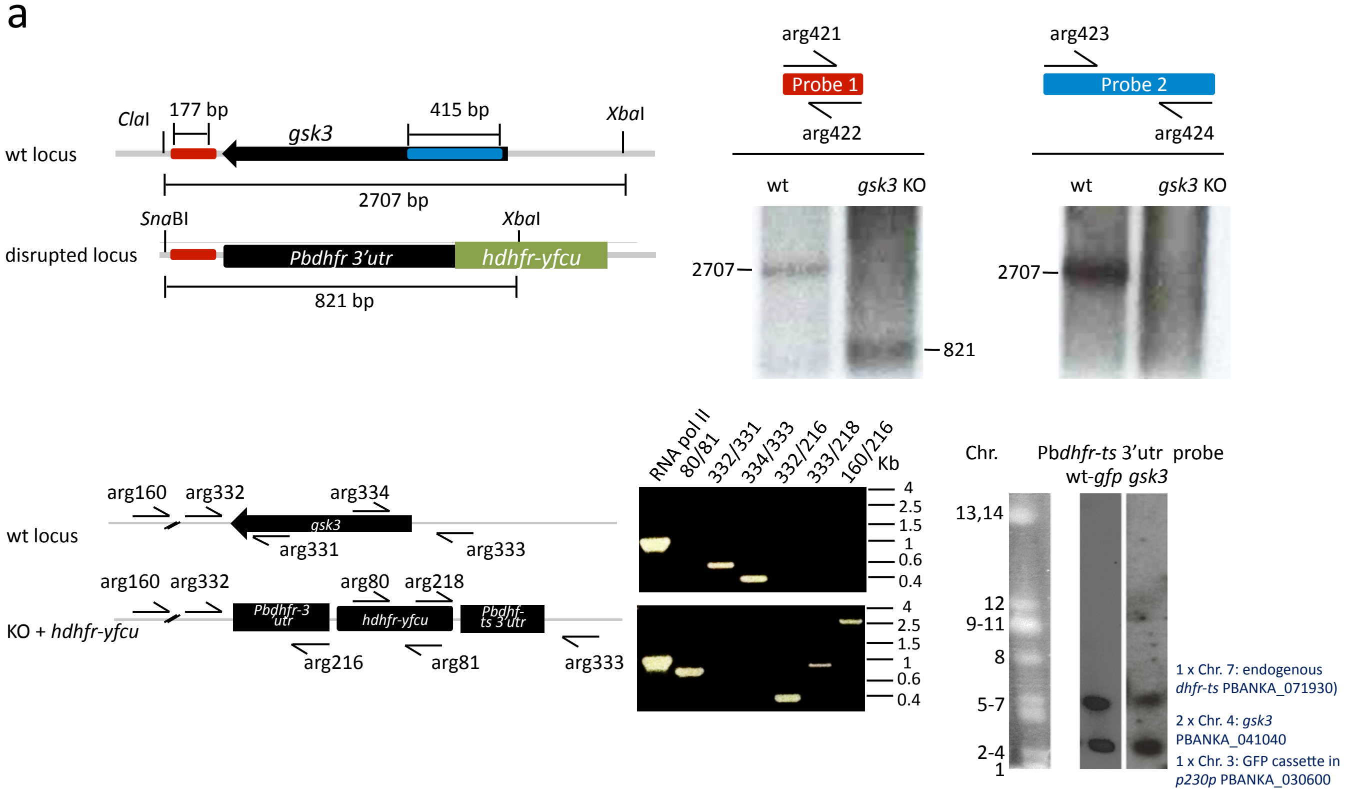

wt locus
ClaI
177 bp
gsk3
415 bp
XbaI
2707 bp
disrupted locus
SnaBI
XbaI
Pbdhfr 3'utr
hdhfr-yfcu
821 bp
arg421
Probe 1
arg422
arg423
Probe 2
arg424
wt
gsk3 KO
2707
821
arg160 arg332
arg334
gsk3
arg331
arg333
arg80 arg218
KO + hdhfr-yfcu
Pbdhfr-3'utr
hdhfr-yfcu
Pbdhfr-ts 3'utr
arg216
arg81
RNA pol II
80/81
332/331
334/333
332/216
333/218
160/216
Kb
4
2.5
1.5
1
0.6
0.4
Chr.
Pbdhfr-ts 3'utr probe
wt-gfp gsk3
13,14
12
9-11
8
5-7
2-4
1
1 x Chr. 7: endogenous dhfr-ts PBANKA_071930)
2 x Chr. 4: gsk3 PBANKA_041040
1 x Chr. 3: GFP cassette in p230p PBANKA_030600

b

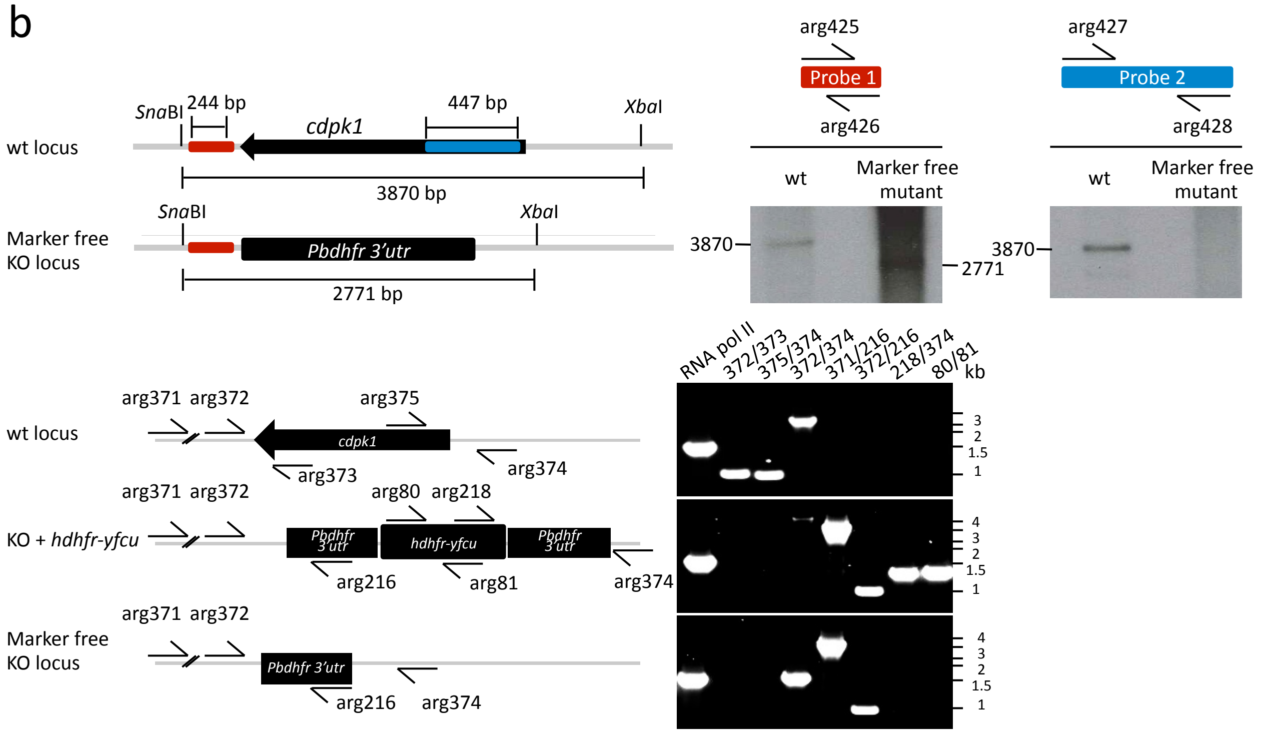

wt locus
SnaBI
244 bp
cdpk1
447 bp
XbaI
3870 bp
Marker free KO locus
SnaBI
XbaI
Pbdhfr 3'utr
2771 bp
arg425
Probe 1
arg426
arg427
Probe 2
arg428
wt
Marker free mutant
3870
2771
arg371 arg372
arg375
cdpk1
arg373
arg374
arg80 arg218
KO + hdhfr-yfcu
Pbdhfr 3'utr
hdhfr-yfcu
arg216
arg81
Marker free KO locus
RNA pol II
372/373
375/374
372/374
371/216
372/216
218/374
80/81
kb
3
2
1.5
1
4

Figure S4

a

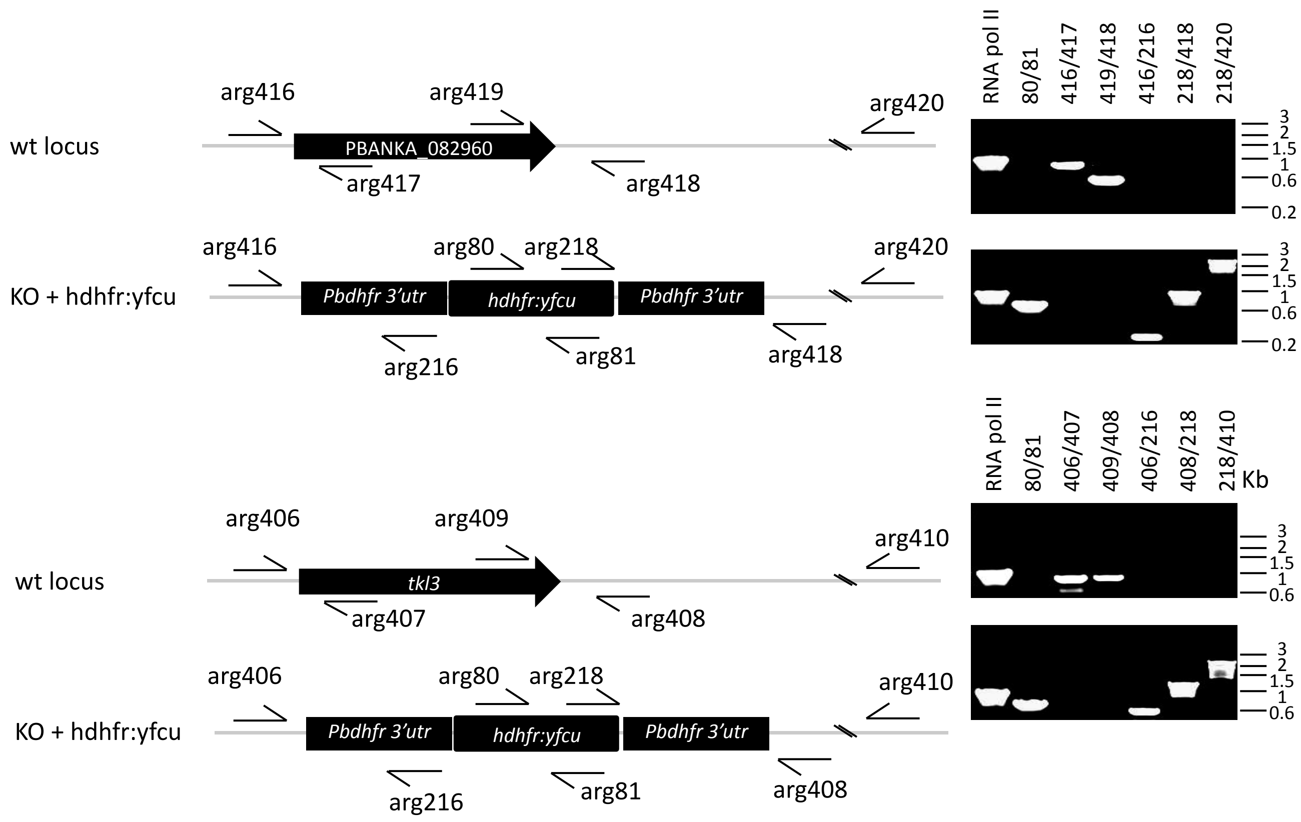

arg416
arg419
arg420
wt locus
PBANKA_082960
arg417
arg418
arg416
arg80
arg218
arg420
KO + hdhfr:yfcu
Pbdhfr 3'utr
hdhfr:yfcu
Pbdhfr 3'utr
arg216
arg81
arg418
RNA pol II
80/81
416/417
419/418
416/216
218/418
218/420
3
2
1.5
1
0.6
0.2
RNA pol II
80/81
406/407
409/408
406/216
408/218
218/410
Kb
arg406
arg409
arg410
wt locus
tkl3
arg407
arg408
arg406
arg80
arg218
arg410
KO + hdhfr:yfcu
Pbdhfr 3'utr
hdhfr:yfcu
Pbdhfr 3'utr
arg216
arg81
arg408

b

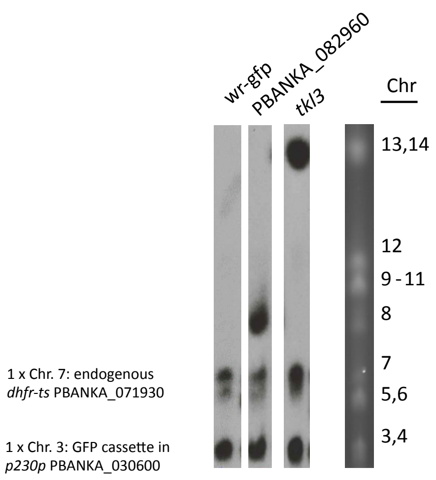

wr-gfp
PBANKA_082960
tkl3
Chr
13,14
12
9 - 11
8
7
5,6
3,4
1 x Chr. 7: endogenous
dhfr-ts PBANKA_071930
1 x Chr. 3: GFP cassette in
p230p PBANKA_030600

Figure S5

a

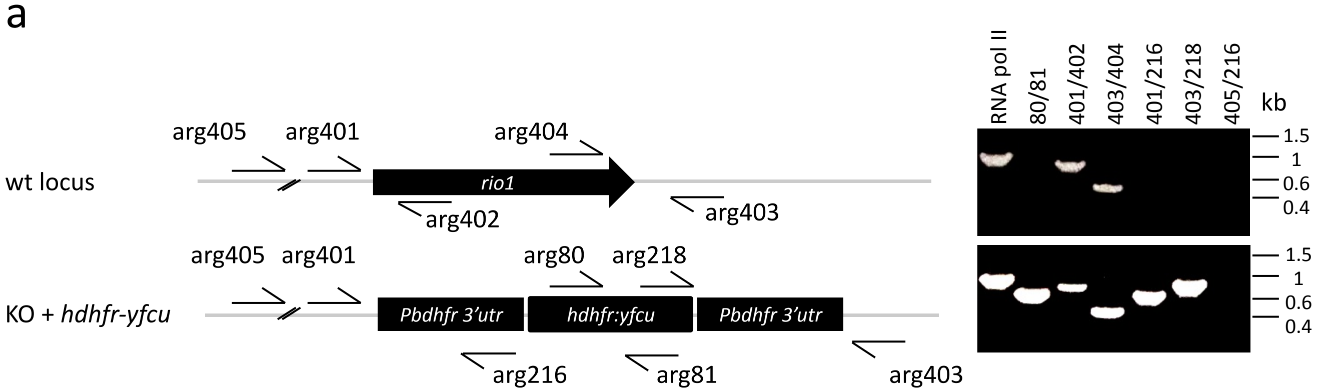

wt locus
KO + hdhfr-yfcu
arg405
arg401
arg404
arg402
arg403
rio1
arg80
arg218
Pbdhfr 3'utr
hdhfr:yfcu
arg216
arg81
RNA pol II
80/81
401/402
403/404
401/216
403/218
405/216
kb
1.5
1
0.6
0.4

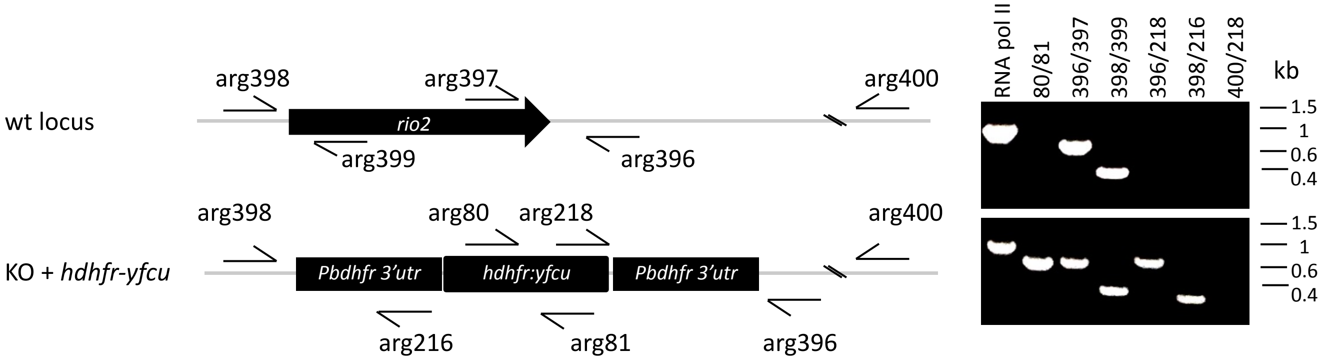

wt locus
KO + hdhfr-yfcu
arg398
arg397
arg400
arg399
arg396
rio2
arg80
arg218
Pbdhfr 3'utr
hdhfr:yfcu
arg216
arg81
RNA pol II
80/81
396/397
398/399
396/218
398/216
400/218
kb
1.5
1
0.6
0.4

b

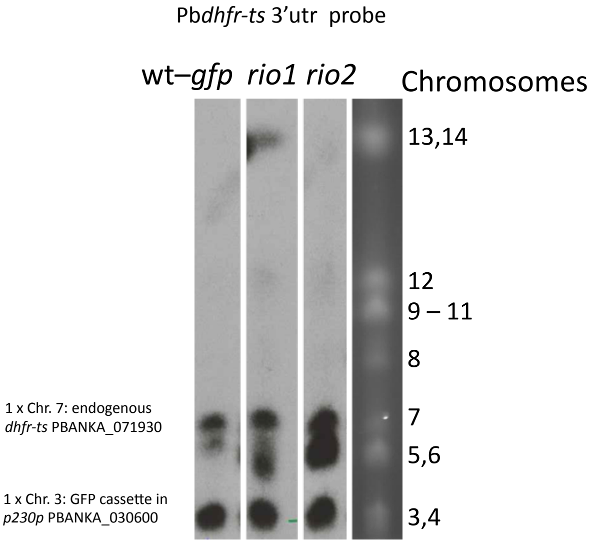

Pbdhfr-ts 3'utr probe
wt–gfp
rio1
rio2
Chromosomes
13,14
12
9 – 11
8
7
5,6
3,4
1 x Chr. 7: endogenous dhfr-ts PBANKA_071930
1 x Chr. 3: GFP cassette in p230p PBANKA_030600

SUPPLEMENTAL FIGURE TITLES AND LEGENDS

Figure S1. Validation of barcode sequencing to phenotype multiple *P. berghei* mutants in the same mouse. Related to Fig. 2 and Fig. 3.

a. Schematic of the barcode module illustrating primer annealing sites and amplification steps. See supplemental information for oligonucleotide sequences.

b. Southern hybridization of chromosomes separated by pulsed-field gel electrophoresis with a ~500 bp probe corresponding to the *dhfr-ts* 3' UTR that is present as two copies in each *Plasmo*GEM vector. Samples were from day 8 post transfection of the experiment shown in Fig. 2b (left panel). In the background strain the probe hybridizes to the *dhfr-ts* locus in chromosome 7, which appears to be polymorphic and to a GFP cassette integrated in the p230p locus of chromosome 3.

c. Agarose gel electrophoresis of PCR products across the predicted integration sites from the same experiment as **b**, supporting genomic integration for 17 of the 22 vectors whose barcodes are detected in Fig. 2b. Oligonucleotides were designed to amplify from the selection cassette (arg218 or arg216) across the shorter homology arm into the flanking genomic sequence. For each gene the predicted product size is stated in kb before the gene name. Note that the long homology arms of *Plasmo*GEM vectors can give rise to false negative results from PCR genotyping for some genes.

d. Comparison of sequencing libraries prepared from the same sample either by nested PCR (as shown in panel a), or by conventional adapter ligation. Barcode counts are expressed as $\log_{10}$ of relative abundance. The dynamic range of the method spans five orders of magnitude, but correlation between methods was highest during the upper two decades. The latter therefore defines the range in which we consider barseq counts reliable. The data shown are representative of six samples analysed in this way.

Figure S2. Additional data illustrating the output of barseq screens. Related to Fig. 3.

a. Relative abundance of barcodes in the input vector pools of a typical experiment (left bar chart) is compared to barcodes counted after transfecting the same vector pool and applying drug selection for 7 days. Error bars (right chart only) give standard deviations from three transfections with the same vector pool. Read counts were normalized to the total number of relevant barcode counts for a given sample. Raw data for this experiment are shown in the Supplemental Spreadsheet.

b. **Fitness of four protein kinase mutants that were detected only in a second pass barseq experiment from which fast growing mutants except the reference genes had been excluded.** Error bars show standard deviations from four independent transfections. Asterisks indicate significant differences determined by a two sided T-test corrected for multiple testing (p < 0.01**; p < 0.05*).

Figure S3. Genotype confirmation for *gsk3* and *cdpk1* knock out clones. Related to Figs. 5 and 6.

a. Southern blot of restricted gDNA, PCR genotyping and hybridization of separated chromosomes showing disruptions of *gsk3*.

b. Genotyping by PCR and Southern blot of a *cdpk1* deletion mutant after removal of the resistance cassette under negative selection.

Figure S4. Genotype confirmation for PBANKA_082960 and *tkl3* knock out clones. Related to Fig. 5.

a. PCR genotyping strategy and PCR products analysed by agarose gel electrophoresis.

b. Southern hybridization of separated chromosomes before cloning showing integration of a targeting vector for PBANKA_082960 and *tkl3* into the target chromosomes 8 and 13, respectively.

Figure S5. Additional genotype confirmation of *rio* kinase mutants. Related to Fig. 5.

a. Genotyping by PCR of *rio1* and *rio2* mutants after population enrichment by drug selection and two consecutive cloning attempts..

b. Southern hybridization showing integration of the targeting vector for *rio1* and *rio2* into the target chromosomes 14 and 5, respectively.

Supplemental Protocol 1 – Transfection of *P. berghei* parasites

Day 1:
Inject a naïve mouse intraperitoneally with 10^6 *P. berghei* parasites and monitor parasitaemia on Giemsa stained thin blood smears.

Day 3 (in the evening):

Infect a young (200-250 g) Wistar rat by intraperitoneal injection of mouse blood containing 10^6 infected red blood cells diluted to a final volume of 500 µL in phosphate buffered saline (PBS).

Usually day 8 (before 12 pm):

1. When parasitaemia has reached 1-3 %, bleed the rat under isofluorane anaesthesia using a 10 mL syringe loaded with 100 µL of heparin (300 U mL^{-1}) with a 20 G needle.
2. Set up a schizont culture by adding 7 mL of infected blood to 200 mL of schizont culture medium in a sterile 500 mL round bottom culture flask. Gas the culture with malaria gas (5 % CO_2, 5 % O_2, and 90 % N_2) for one minute before closing it tightly. Incubate the culture at 36.5 °C with gentle rotary shaking (just enough to prevent blood from settling).

Schizont culture medium (200 mL): To 144 mL RPMI1640 containing L-glutamine and 25 mM HEPES, without $NaHCO_3$ (Sigma-Aldrich) add 4 mL of 0.5 M $NaHCO_3$ (filter sterilized), 2 mL penicillin/streptomycin (10,000 U mL^{-1}) and 50 mL fetal calf serum (Gibco).

Day 9:

1. At 10 am check schizont maturation on a Giemsa stained thin blood smear. Once schizonts contain individualized merozoites proceed to purification.
2. In each of four 15 mL Falcon tubes prepare a 55 % Nycodenz/PBS cushions (v/v) by mixing 2.75 mL Nycodenz stock-solution (Janse et al., 2006) with 2.25 mL PBS.
3. Pellet the blood containing the schizonts in 50 mL Falcon tubes for 14 min at 300 g with slow acceleration/deceleration. It is important that the centrifuge is at room temperature at all times.
4. Keep 30 mL of supernatant to wash/dilute purified schizonts later on. Remove the supernatant apart from 3-4 mL at the bottom of the tube which will be used to resuspend the cells.
5. Using a plastic Pasteur pipette, gently layer the cells on top of the Nycodenz cushion prepared in step 3.
6. Centrifuge 20 min at 300 g in a swing out rotor again at room temperature (slow acceleration and break, so as not to disturb the gradient).
7. Collect the brown schizont layer at the interface from all gradients into a 15 mL Falcon tube (1 mL from each gradient) and fill it up to the top with the supernatant kept in step 5.

8. Pellet the schizonts by centrifugation (450 g, 3 min, medium acceleration/ deceleration) and discard supernatant.
9. To use schizonts from one rat for 3 replicate transfections of a barcode sequencing experiment add 54 µL (18 µL/transfection) of P3 Primary Cell 4D-Nucleofector (Lonza) to the pelleted schizonts and resuspend them gently.
10. Mix the schizont suspension with 5 µL of digested DNA and transfer 25 µL of the mix into each of three individual wells of a 16-well Nucleocuvette Strip and pulse using programme FI-115 of the 4D-Nucleofector device.
11. Aspirate schizonts using 1 mL insulin syringe, previously loaded with 100 µL of pre-warmed incomplete RPMI.
12. Inject immediately intra-venously into BALB/c mice.

Day 10:
Early in the morning take thin blood smears to calculate transfection efficiency before starting drug selection with drinking water containing pyrimethamine (0.07 mg/mL).

Supplemental Protocol 2 – Genomic DNA isolation (phenol-chloroform method) for barcode sequencing

1. Lyse the red blood cells by adding 1 mL of ammonium chloride solution (0.15 M NH_4Cl, 0.01 M $KHCO_3$, 0.001 M EDTA) to the collected blood. Incubate for 2 min on ice.
2. Pellet the parasites (3000 g) and resuspend them in 500 µL of TNE buffer (10 mM Tris pH 8.0, 5 mM EDTA pH 8.0, 100 mM NaCl).
Add 2 µL of RNase A (20 mg/mL), 55 µL of 10 % SDS and incubate for 10 min at 37 °C.
3. Add 10 µL of proteinase K (20 mg/mL), vortex and incubate at 37 °C for 30 min.
Add 500 µL of buffered phenol:chloroform:isoamylalcohol (25:24:1), invert several times and centrifuge for 5 min at 10,000 g.
4. Transfer the aqueous upper phase into a new tube. Add 500 µL of chloroform:isoamylalcohol (24:1), invert samples several times and centrifuge for 5 min at 10,000 g.
5. Transfer the new aqueous upper phase to a new tube, and precipitate DNA overnight at -20 °C with 500 µL of isopropanol.

OLIGONUCLEOTIDE SEQUENCES

Primer name	Sequence	Target/Comment
arg00080	GACTTCTGTAGCCATGATAGC	hdhfr::yfcu
arg00081	CGCCACACTACATGGTGAG	hdhfr::yfcu
arg00084	AAAGAATTCTGATGGTTTACAATCACC	RNApol II
arg00085	AAAGCGGCCGCTTTCTTCCTGCATCTCCTC	RNApol II
arg00160	AGCGAGTTCCCGTGCACTCA	
arg00331	TTGAACATTTGCGCATATATTGG	
arg00332	GTGCCAAATTATTATGGTATACC	
arg00333	TGATCTAGAGATAAAAGAGGAC	
arg00334	CTTCATATTTTACCTGTATGCC	
arg00421	GTGCCAAATTATTATGGTATAC	
arg00422	CCAGAATAATTTTGTTAGAATATAG	
arg00423	GTACAGTTTGTGGTATATATTCC	
arg00424	GGTGATAATAATGCATGCCAAG	
arg00371	CCGGTATTTATCATCCAAGAG	
arg00372	CATTTGTTCATGCAATCATTCG	
arg00373	CCCACTACCTAATTTCCGAAC	
arg00374	GCATCATTGACACGAACTCG	
arg00375	GTGAAGAGAGGCTAAGGAGG	
arg00425	GGTTCTATCTGTTTCATGTAC	
arg00426	CCTCAGAAAATGAATGGCAG	
arg00427	GGTGTAATCAAAGTAAAAGTGC	
arg00428	CTCCACCTTCATAAAATTCGG	
arg00396	GGCATGCCGTATTTTCCATG	
arg00397	ATGCATATTCTTTTGTTGCAGC	
arg00398	CACCCATCCAAACATATAAAAG	
arg00399	CTAGTAATAGTCAGTCTGGGG	
arg00400	TCCAAAGCGTTATGCCAAGTGT	
arg00401	GCAACTCGTTATATATTTCCG	
arg00402	CTTTTCCAGAACTAACTACTCC	
arg00403	CTTTTCTTGTAACACCCTCAG	
arg00404	TGGAACTACAAAATAGATTTCG	
arg00405	GCTCAAGCAACAGCAGGAC	
arg00496	CGTTTTTCTTACTTATATATTTATACCAATTGATTGT	PFGE probe
arg00497	TCGAAATTGAAGGAAAAAACATCATTTG	PFGE probe
arg00216	CGGGGCCCTTATGCATAATC	
arg00218	CTTTGGTGACAGATACTACTG	
arg00406	ATCCAAATATGGTATTTTGAGC	
arg00407	CTGGAGATTCGTTTTGTTCAC	
arg00408	TTGTGTTTGCCGCATGTTGC	
arg00409	GATATTCCTAAAGATCTATCTG	
arg00410	ACGACAATGTGCATGCCTCA	
arg00416	CGTACTTGAATAGCTGTCTAC	
arg00417	CCCCAATATAATGAATATTCTG	
arg00418	GAGCATTCCGCAAAGTATGTC	
arg00419	GGGAACACATCCTTTTAGTTC	
arg00420	AGCCATTACCCGTTGTTTCG	
arg00215	TCATTCTTCGAAAACGATCT	zeocin
arg00083	CCGCCTACTGCGACTATAGA	zeocin
arg00218	CTTTGGTGACAGATACTACTG	
arg00057	GAAACACAATGTTGAAATTC	*map2*
arg00102	GTGTAGAAGTAAATTCATACCC	*srpk2*
arg00370	GGTTATGAGAAGTTAAACTACG	*cdpk4*

SUPPLEMENTAL REFERENCE

Janse, C.J., Ramesar, J., and Waters, A.P. (2006). High-efficiency transfection and drug selection of genetically transformed blood stages of the rodent malaria parasite Plasmodium berghei. Nature protocols *1*, 346-356.
